# Gut microbiome and stages of diabetes in middle-aged adults: CARDIA microbiome study

**DOI:** 10.1186/s12986-022-00721-0

**Published:** 2023-01-05

**Authors:** Yi-Han Hu, Katie Meyer, Anju Lulla, Cora E. Lewis, Mercedes R. Carnethon, Pamela J. Schreiner, Stephen Sidney, James M. Shikany, Osorio Meirelles, Lenore J. Launer

**Affiliations:** 1 Laboratory of Epidemiology and Population Sciences, National Institute On Aging, 251 Bayview Blvd, Baltimore, MD 21224 USA; 2 Nutrition Research Institute, University of North Carolina at Chapel Hill, Kannapolis, NC USA; 3 Department of Nutrition, University of North Carolina at Chapel Hill, Chapel Hill, NC USA; 4 Department of Epidemiology, University of Alabama at Birmingham, Birmingham, AL USA; 5 Department of Preventive Medicine, Feinberg School of Medicine, Northwestern University, Chicago, IL USA; 6 Division of Epidemiology and Community Health, University of Minnesota, Minneapolis, MN USA; 7 Kaiser Permanente Medical Center Program, Oakland, CA USA; 8 Division of Preventive Medicine, School of Medicine, University of Alabama at Birmingham, Birmingham, AL USA

**Keywords:** Diabetes, Insulin resistance, Diabetes duration, Gut microbiota composition, Population-based

## Abstract

**Background:**

Animal and human studies suggest the gut microbiome is linked to diabetes but additional data are needed on the associations of the gut microbiome to specific diabetes characteristics. The aim of this study was to examine the associations of gut microbiome composition to insulin resistance [Homeostatic Model Assessment of Insulin Resistance (HOMA-IR)], duration of diabetes, and 4 stages of diabetes [normoglycemia, pre-diabetes, and diabetes with (+) and without (−) medication for diabetes].

**Methods:**

Data are from a sub-sample (n = 605) of Black and White participants from the 30-year follow-up exam of the prospectively followed community-based Coronary Artery Risk Development in Young Adults cohort (2015–2016; aged 48–60 years). Stool samples were collected and sequenced using the 16S ribosomal RNA method. Microbial measures included: α diversity (within-person), β diversity (between-person), and taxonomies. All analyses were adjusted for demographic, clinical, lifestyle factors, and use of relevant medications (full adjustment). Multivariate linear regression models were used to assess the association of diabetes characteristics with α diversity and genera abundance, while the association with β diversity was analyzed using permutational multivariate analysis of variance. Statistical significance was set to *p*-value < 0.05 for α and β diversity analyses and to q-value < 0.1 for genera abundance analyses.

**Results:**

There were 16.7% of participants with pre-diabetes, and 14.4% with diabetes (9% diabetes+) with median (interquartile range) diabetes duration of 5 (5–10) years. In the fully adjusted models, compared to those with no diabetes, longer diabetes duration and the diabetes + group had a lower α diversity. There were significant differences in β diversity across diabetes-related characteristics. A significantly reduced abundance of butyrate-producing genera was associated with higher HOMA-IR (ex., *Anaerostipes* and *Lachnospiraceae_UCG.004*), longer diabetes duration (ex., *Agathobacter* and *Ruminococcus*), and diabetes + (ex., *Faecalibacterium* and *Romboutsia*).

**Conclusions:**

Our results suggest that an adverse alteration of gut microbiome composition is related to higher insulin resistance, longer diabetes duration, and is present in those persons with diabetes using medications. These diabetes-related characteristics were also associated with lower levels of certain butyrate-producing bacteria that produce health-promoting short‐chain fatty acids. Understanding the role of gut microbiota in glucose regulation may provide new strategies to reduce the burden of diabetes.

**Supplementary Information:**

The online version contains supplementary material available at 10.1186/s12986-022-00721-0.

## Background

The diverse species of bacteria in the gut microbiome have been suggested to play a role in a variety of metabolic disorders including type 2 diabetes (T2D) [1, 2]. The microbiome, together with dietary-derived microbial metabolites, bears the potential to affect adiposity, glucose metabolism, and insulin sensitivity [3–5]. A growing number of animal models support mechanisms through which the gut microbiome may influence the development of insulin resistance and T2D [6, 7]. However, there is also evidence that the host metabolic state, as well as pharmacologic treatments, may impact the gut microbiota [5, 8, 9]. Understanding the role of gut microbiota in insulin resistance and glucose regulation may provide new and more individualized strategies for clinical prevention and management of T2D [10].

Community-based studies with a wide range of microbiome profiles and diabetes characteristics can contribute further insight into possible clinical implications of how microbiota may be influenced by, or influence T2D. The majority of previous human observational studies were conducted in patient populations or using case–control designs or inclusion of participants with a narrow range of diabetes stages, thereby narrowing the range of T2D and microbiome exposure [8, 11–14]. Further, extant studies do not account for duration of diabetes, which may be a factor in the strength of associations between diabetes and the microbiome [2]. Additionally, it is important to understand the association of diabetes to the microbiome, controlling for the socio-demographically diverse population with T2D, as other factors may influence both T2D and the microbiota.

The current study is based on a population-based prospective cohort of Black and White men and women followed for 30 years as a part of the Coronary Artery Risk Development in Young Adults (CARDIA) study. We investigate associations of diabetes-related characteristics with gut microbial diversity and taxonomic composition.

## Research design and methods

### Study design

The CARDIA study is a multicenter, longitudinal cohort study of 5115 White and Black men and women from four US metropolitan areas: Birmingham, AL; Chicago, IL; Minneapolis, MN; and Oakland, CA. The details of its design are described elsewhere [15]. Participants were aged 18 to 30 years at baseline in 1985–1986 (Y0) and attended follow-up exams in years 2, 5, 7, 10, 15, 20, 25, and 30 (Y2–Y30) after baseline, with 71% retention among the surviving cohort at Y30. As part of the ongoing cohort study, 615 participants were recruited into a microbiome sub-study at Y30, described briefly below and in detail elsewhere [16]. The comparison in sample characteristics between those included and not included in the microbiome study is presented in Additional file 1: Table S1. All CARDIA field centers received their respective institutional review board approvals, and participants provided written informed consent to all study components at each exam.

### Gut microbiome data collection, assay, and preprocessing

Briefly, we followed standard protocols for collection and processing of stool samples [17, 18], as previously described [16]. Participants completed the stool collection in their home using collection tubes pre-filled with RNAlater, along with a short survey pertaining to covariates relevant for the microbiome study, and shipped their samples with provided ice packs and insulated shipping containers and completed questionnaire overnight to the study lab at the Nutrition Research Institute at the University of North Carolina, Chapel Hill, where samples were stored at − 80 °C until processing.

DNA was extracted from 0.2 g of stool using the MoBio PowerSoil kit (or Qiagen DNeasy PowerSoil after the purchase of MoBio by Qiagen). The V3–V4 hypervariable regions were amplified and sequenced using the Illumina MiSeq platform (2 × 300). Forward sequences were processed (quality trimming, denoising, and chimera-removal) through the divisive amplicon denoising algorithm (DADA2) package in R14. The DADA2-formatted Silva database (silva_nr_v138_train_set.fa.gz) was used to assign taxonomy [19].

### Assessment of diabetes-related characteristics

Insulin resistance, diabetes duration, and stages of diabetes were the primary diabetes-related characteristics in the present study (Additional file 1: Fig. S1). We used the Homeostatic Model Assessment of Insulin Resistance (HOMA-IR) as a surrogate measure for insulin resistance. HOMA-IR was calculated as follow: [fasting insulin (uU/mL) × fasting glucose (mmol/L)]/22.5 [20]. The average of Y25 and Y30 HOMA-IR was used in the analyses.

Pre-diabetes and T2D were identified according to American Diabetes Association (ADA) criteria [21]. T2D was determined based on the presence of any of the following: a fasting serum glucose (FSG) ≥ 126 mg/dL (available at Y0, Y7 and afterward), or a 2-h (2 h) post-load glucose (2 h-PG) ≥ 200 mg/dL during a 75-g oral glucose tolerance test (available at Y10, Y20 and Y25), or a hemoglobin A1C (HbA1c) ≥ 6.5% (available at Y20 and Y25), or self-report of diabetes medications (e.g., oral hypoglycemic medications or insulin) use [22]. Similarly, pre-diabetes was defined as having a FSG of 100–125 mg/dL, or a 2 h-PG 140–199 mg/dL, or an HbA1c 5.7–6.4% in both Y25 and Y30 and no report of diabetes and no use of diabetes medications across 9 exams.

The duration of diabetes was indicated by the number of years of diabetes and calculated based upon the presence of diabetes at each exam beginning at Y2 [22]. For example, a participant who developed diabetes at Y10 was assigned a total of 20 years as the cumulative duration of diabetes, while a half of year was assigned to participants who developed diabetes at Y30. We characterized diabetes stages into four groups (1) normal; (2) pre-diabetes, (3) diabetes patients without treatment (T2D−, who did not receive treatment at both Y25 and Y30), and (4) diabetes with treatment (T2D+, who received treatment at either Y25 or Y30).

### Covariates and confounders

Possible confounders of the associations between gut microbiome and aforementioned diabetes characteristics were identified from the literature [2, 12]. The majority of covariate measures were collected at the Y30 exam, with missing values replaced by Y25 covariates. Age, sex, race, highest educational attainment (high school or less, college, or graduate school), current smoking status (yes/no), current alcohol use (yes/no), and medication use (yes/no), including proton pump inhibitor (PPIs), antihypertensive and lipid-lowering, were assessed through self-reported questionnaires. Body mass index (BMI), resting systolic blood pressure (SBP), and resting diastolic blood pressure (DBP) were collected by trained staff according to a standard protocol. A total physical activity score was calculated based on the Physical Activity Questionnaire [23]. Diet quality score was derived from the interviewer-administered Diet History at the Y20 Exam, as previously described [24].

### Participants in the current analyses

In the microbiome sub-cohort of the 615 participants, 607 had viable DNA samples for sequencing. From these 607 participants, we excluded one participant who had diabetes at baseline and one participant with missing smoking status at both Y25 and Y30 exams, resulting in 605 participants for analyses on gut microbiome and diabetes duration and stages of diabetes. For analyses of insulin resistance, we additionally excluded one participant without data on insulin resistance at Y25 or Y30, yielding an analytic sample of 604 (Additional file 1: Fig. S2).

### Statistical analyses

We examined associations between gut microbiome composition, measured by within-person α diversity and between-person β diversity, and insulin resistance, diabetes duration, and stages of diabetes and specific taxa with the set of diabetes-related characteristics. We focused our primary analysis on genera, the lowest level of taxonomy from our data.

The α diversity (Shannon index and richness) and β diversity (Bray–Curtis index) at the genus level were calculated using the R package vegan [25]. The α diversity represents the complexity of composition within members of a group. In general, high α diversity is favorable to our health. We calculated α diversity measures using raw genera counts. The β diversity represents the similarity of microbial composition between groups of interest, with high β diversity indicating low similarity. For β diversity analysis, raw genera counts were transformed as log10[(*RC*/n)(x/N) + 1], where RC is the total raw count for a participant, n is the total count across all genera for a participant, x is the total across all taxa and participants, and N is the total number of participants, as previously described [16, 26]. To investigate difference in β diversity between groups, HOMA-IR was reclassified into two groups based on the median (i.e., ≤ median [2.19], and > median), while diabetes duration was reclassified into three groups (i.e., normal/pre-diabetes, newly diagnosed diabetes [duration < 5 years], and established diabetes [duration ≥ 5 years]).

The associations of α diversity measures with insulin resistance, diabetes duration, and stages of diabetes were assessed by linear regression, adjusting for four sets of covariates sequentially. In model 1, we adjusted for the sequencing run. In model 2, age, sex, race, education level, and field center were added. In model 3, we additionally adjusted for smoking, alcohol use, BMI, physical activity, and diet quality score. Last, in model 4 (the fully adjusted model), we additionally adjusted for the use of PPIs and lipid-lowering drugs. We analyzed associations of β diversity with newly categorized insulin resistance, diabetes duration, and stages using permutational multivariate analysis of variance (PERMANOVA) with covariate adjustment; a *p*-value was generated through 1000 permutations. To examine post hoc pairwise comparisons, we conducted additional PERMANOVA tests for each pair within categorized diabetes duration and stages. For visualization, principal coordinates analysis (PCoA) based on the Bray–Curtis dissimilarity matrix was applied. We present the first two dimensions from the PCoA according to two groups of HOMA-IR, three categories of diabetes duration, and four diabetes stages. In both α and β diversity analyses, statistical significance was set at a two-tailed *p* < 0.05.

To limit the possibility of spurious findings due to rare taxa, we restricted analyses to those individual taxa with non-zero counts in at least 75% of participants [16]. As a result, the taxa-specific analysis was based on 107 out of initially 375 genera. The log-transformed genera counts (described above) were used for the analyses. Multivariable linear regression models with the same sets of covariates (described above), were conducted to examine the association of diabetes-related characteristics with microbial taxa abundance. To adjust the *p*-value for multiple comparisons, we used the Benjamini–Hochberg method for false discovery rate (FDR). In the taxa-specific analysis, statistical significance was set to FDR-adjusted *p*-value (q-value) < 0.1 [27]. Data analysis was conducted in RStudio version 1.3.959 with R version 4.1.0 (http://www.r-project.org) and SAS version 9.4 (SAS Institute Inc, Cary, NC).

### Sensitivity analyses

We conducted two sets of sensitivity analyses based on model 4. First, we added SBP, DBP, and antihypertensive medication use (binary) (model 5). For insulin resistance and diabetes duration analyses, we also investigated whether diabetes medication use (binary) attenuated the main associations of interest by additionally adjusting for use of diabetes medicines (model 6); while for diabetes stages analysis, we further adjusted for diabetes duration (model 6).

## Results

### Baseline characteristics

Among 605 participants, 417 (68.9%) had no diabetes (normoglycemic), 101 (16.7%) had pre-diabetes, 56 (9.3%) persons with diabetes who were on diabetes treatment (T2D+), and 31 (5.1%) persons with diabetes not on diabetes treatment (T2D−). Fasting glucose concentrations and HOMA-IR scores increased with diabetes stage, with the lowest level in the normoglycemic group {means [standard deviations] (SDs) = 91.9 (7.7) and 2.2 (1.5) for fasting glucose and HOMA-IR, respectively} and the highest level in diabetes + [means (SDs) = 131.7 (53.5) and 5.4 (3.8), respectively]. Participants with treated diabetes had a longer diabetes duration, with a mean (SD) of 8.3 (5.8) years versus 7.5 (6.8) years in T2D−; 50% of T2D+ had been diagnosed with diabetes for 10 years or longer (upper quartile). Characteristics of the study population are shown in Table 1.

**Table 1:** Descriptive statistics of analytic sample by stages of diabetes—CARDIA cohort: Year 30 Exam

	Overall (n = 605) n(%)/M(SD)	Stages of diabetes (n = 605)				*p*^g^
		Normal	Pre-diabetes	Diabetes without treatment	Diabetes with treatment	
		(n = 417, 68.9%)	(n = 101, 16.7%)	(n = 31, 5.1%)	(n = 56, 9.3%)	
		n (%)/M(SD)	n(%)/M(SD)	n(%)/M(SD)	n(%)/M(SD)	
1. Diabetes-related variables						
Fasting glucose (mg/dL)^a^	100.2 (24.8)	91.9 (7.7)	107.5 (6.1)	130.5 (47.1)	131.7 (53.5)	< 0.01
HOMA-insulin resistance^a,b^	2.9 (2.3)	2.2 (1.5)	3.8 (2.4)	5.0 (2.9)	5.4 (3.8)	< 0.01
Diabetes duration, median (IQR)^c^	5 (5–10)	N/A	N/A	5 (5–10)	10 (5–10)	N/A
Metformin use (%)	43 (7.1)	N/A	N/A	N/A	43 (76.8)	N/A
2. Socio-demographics						
Age^a^	55.2 (3.5)	55.1 (3.4)	55.4 (3.5)	54.9 (4.4)	55.8 (3.6)	0.32
Male (%)	272 (45.0)	168 (40.3)	63 (62.4)	18 (58.1)	23 (41.1)	0.30
Black race (%)	275 (45.5)	167 (40.0)	50 (49.5)	16 (51.6)	42 (75.0)	< 0.01
Highest education (%)						0.36
High school or less	206 (34.1)	132 (31.7)	39 (38.6)	14 (45.2)	21 (37.5)	
College	261 (43.1)	181 (43.4)	40 (39.6)	14 (45.2)	26 (46.4)	
Graduate school	138 (22.8)	104 (24.9)	22 (21.8)	3 (9.6)	9 (16.1)	
Field center (%)						0.72
Birmingham, AL	98 (16.2)	69 (16.5)	17 (16.8)	4 (12.9)	8 (14.3)	
Chicago, IL	295 (48.8)	194 (46.5)	51 (50.5)	16 (51.6)	34 (60.7)	
Minneapolis, MN	110 (18.2)	77 (18.5)	18 (17.8)	6 (19.4)	9 (16.1)	
Oakland, CA	102 (16.8)	77 (18.5)	15 (14.9)	5 (16.1)	5 (8.9)	
3. Clinical measures^a^						
BMI	29.4 (6.2)	28.1 (5.8)	31.6 (6.0)	31.9 (3.6)	33.4 (7.3)	< 0.01
Systolic blood pressure (mmHg)	119.3 (16.1)	117.2 (15.7)	124.1 (14.5)	124.8 (16.4)	123.5 (18.5)	< 0.01
Diastolic blood pressure (mmHg)	72.9 (11.0)	71.5 (11.0)	76.8 (9.6)	77.3 (10.9)	74.2 (11.2)	< 0.01
4. Health behavior						
Current smoker (%)	81 (13.4)	42 (10.1)	18 (17.8)	12 (38.7)	9 (16.1)	< 0.01
Alcohol use (%)	469 (77.5)	318 (76.3)	86 (85.1)	26 (83.9)	39 (69.6)	0.29
Physical activity, median (IQR)^d^	267 (128–504)	280 (144–536)	276 (116–510)	300 (120–426)	170 (82.5–332.5)	< 0.01
5. Medication use (%)						
Proton pump inhibitor	47 (7.8)	23 (5.5)	12 (11.9)	4 (12.9)	8 (14.3)	< 0.05
Lipid-lowering	123 (20.3)	67 (16.1)	19 (18.8)	6 (19.4)	31 (55.4)	< 0.01
High blood pressure	180 (29.8)	91 (21.8)	37 (36.6)	15 (48.4)	37 (66.1)	< 0.01
6. Diet quality score (standardized), median (IQR)^e^	− 0.07 (− 0.70 to 0.70)	− 0.06 (− 0.68 to 0.85)	− 0.37 (− 0.99 to 0.39)	− 0.62 (− 0.99 to 0.16)	− 0.37 (− 0.84 to 0.24)	< 0.01

*Data source*: The Coronary Artery Risk Development in Young Adults (CARDIA), 1985–2016

All covariates were collected at the Y30 Exam. Missing covariates were updated using Y25 Exam information

N/A, not applicable; HOMA-Insulin resistance, Homeostatic Model Assessment for Insulin Resistance; IQR, interquartile range

^a^Data is reported as mean and SD

^b^Valid sample size for insulin resistance was 604

^c^Statistics were calculated among participants with diabetes

^d^Total activity intensity in the past year

^e^Derived variable from the diet history at the Y20 Exam

^f^*p*-value was based on the chi-square test for male, race, education level, field center, smoking, alcohol use, or medication use; analysis of variance was used to estimate *p*-value for the remaining variables

### Insulin resistance and gut microbiota diversity and composition

**Fig. 1:**
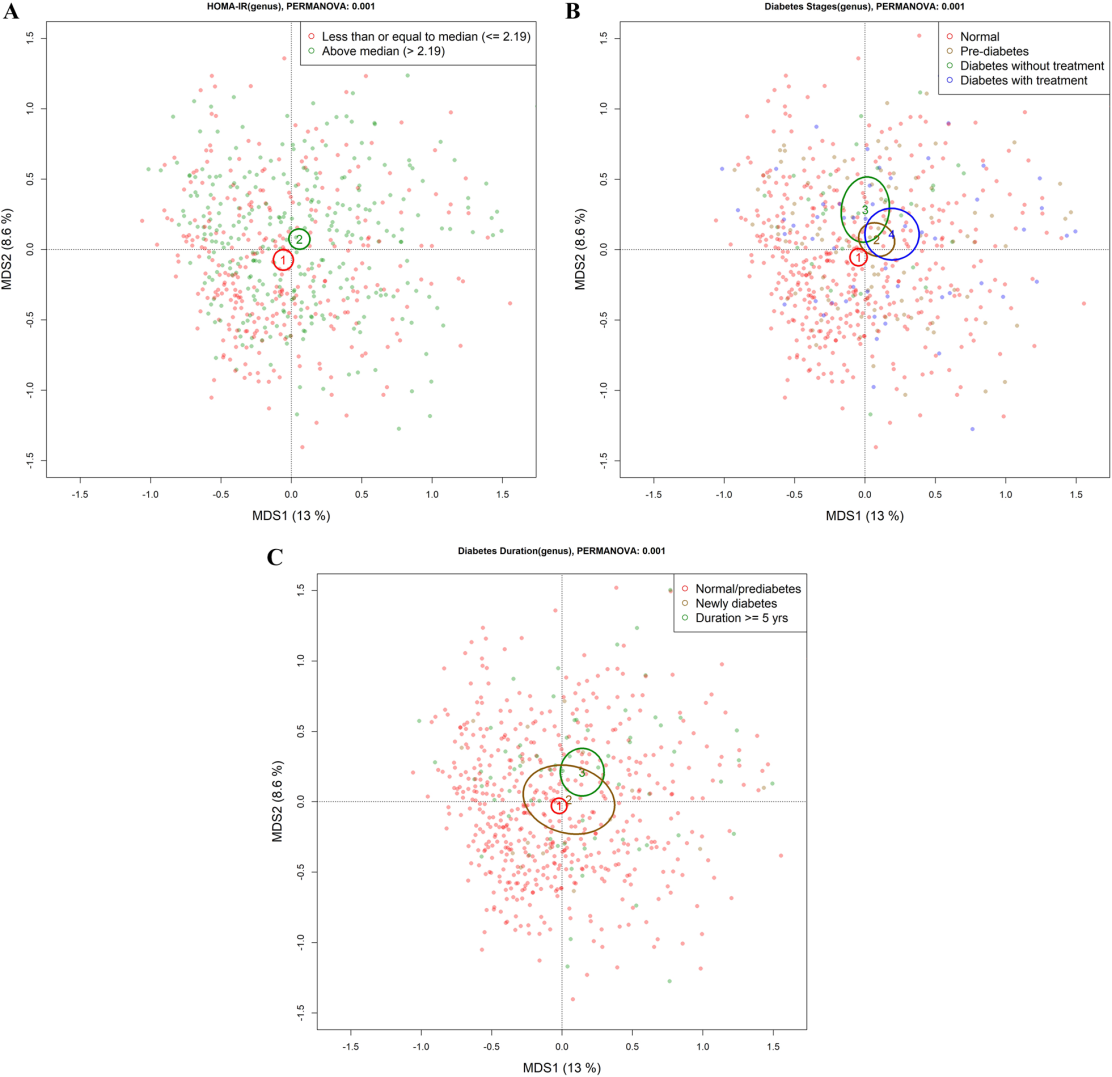
Principal coordinates analysis (PCoA) biplots of associations of microbial dissimilarity with insulin resistance (less than or equal to median, above median), diabetes duration (Normal/pre-diabetes, newly diagnosed diabetes [< 5 years, median], and established diabetes [≥ 5 years]), and stages of diabetes. **A** Homeostatic Model Assessment for Insulin Resistance (HOMA-IR); **B** Stages of diabetes (4 stages including normal, pre-diabetes, diabetes without treatment, and diabetes with treatment); **C** Diabetes duration. PCoA, principal coordinates analysis; MDS, multidimensional scaling. The circles and error bars indicate the centroid and standard errors. The log-transformed genera counts were used for the analyses and visualizations. *P*-values for the comparison of gut microbiota composition were estimated from permutation multivariate analysis of variance (PERMANOVA) with 1000 permutations. All *p*-value were less than 0.05 for all diabetes-related characteristics in the multivariable-adjusted models. *Data source*: The Coronary Artery Risk Development in Young Adults (CARDIA), 1985–2016

Higher HOMA-IR was associated with lower genus richness {β [95% confidence interval (CI)] =  − 0.04 [− 0.08, − 0.01]} (Table 2, Model 1). The inverse association between insulin resistance and richness attenuated to non-significant after adjusting for behavioral factors (Table 2, Model 3). Insulin resistance was significantly associated with β diversity based on PERMANOVA tests (*p*-values < 0.001) at all levels of multivariable adjustment. The PCoA of gut microbiota plot also showed that the gut microbiota community composition differed between HOMA-IR groups (dichotomized by median) (Fig. 1A; Additional file 1: Table S3).

**Table 2:** Multivariable-adjusted associations of α diversity measures with insulin resistance, diabetes duration, and stages of diabetes

	HOMA-IR		Diabetes duration		Stages of diabetes (reference: normal, n = 417)					
					Pre-diabetes		Diabetes without treatment		Diabetes with treatment	
	(n = 604)		(n = 605)		(n = 101)		(n = 31)		(n = 56)	
	β	95% CI	β	95% CI	β	95% CI	β	95% CI	β	95% CI
*Shannon index*										
Model 1	− 0.02	− 0.05, 0.01	− 0.02*	− 0.05, − 0.003	− 0.18	− 0.40, 0.04	− 0.18	− 0.54, 0.19	− 0.28*	− 0.56, − 0.003
Model 2	− 0.02	− 0.05, 0.02	− 0.03*	− 0.05, − 0.004	− 0.18	− 0.40, 0.05	− 0.16	− 0.52, 0.21	− 0.28	− 0.56, 0.01
Model 3	0.003	− 0.04, 0.04	− 0.02*	− 0.05, − 0.0004	− 0.13	− 0.35, 0.10	− 0.04	− 0.42, 0.33	− 0.23	− 0.52, 0.07
Model 4	− 0.003	− 0.05, 0.04	− 0.03*	− 0.05, − 0.005	− 0.15	− 0.37, 0.08	− 0.06	− 0.44, 0.31	− 0.30*	− 0.60, − 0.01
*Richness*										
Model 1	− 0.04*	− 0.08, − 0.01	− 0.04*	− 0.06, − 0.02	− 0.26*	− 0.48, − 0.05	− 0.43*	− 0.79, − 0.07	− 0.51*	− 0.78, − 0.23
Model 2	− 0.04*	− 0.08, − 0.01	− 0.04*	− 0.07, − 0.02	− 0.28*	− 0.50, − 0.07	− 0.42*	− 0.78, − 0.06	− 0.53*	− 0.82, − 0.25
Model 3	− 0.004	− 0.05, 0.04	− 0.04*	− 0.06, − 0.02	− 0.21	− 0.43, 0.01	− 0.26	− 0.62, 0.11	− 0.44*	− 0.72, − 0.15
Model 4	− 0.01	− 0.05, 0.03	− 0.04*	− 0.06, − 0.02	− 0.22	− 0.44, 0.004	− 0.27	− 0.63, 0.10	− 0.50*	− 0.79, − 0.20

*Data source*: The Coronary Artery Risk Development in Young Adults (CARDIA), 1985–2016

HOMA-IR and diabetes duration were treated as continuous variables

Model 1adjusted for sequencing run. Model 2 additionally adjusted for age, sex, race, field center, and education. Model 3 additionally adjusted for physical activity, smoking status, alcohol use, and diet quality score. In Model 4, medication use, such as proton pump inhibitor and lipid-lowering, was added

HOMA-IR, Homeostatic Model Assessment for Insulin Resistance

*Significance based on *p*-value < 0.05

**Fig. 2:**
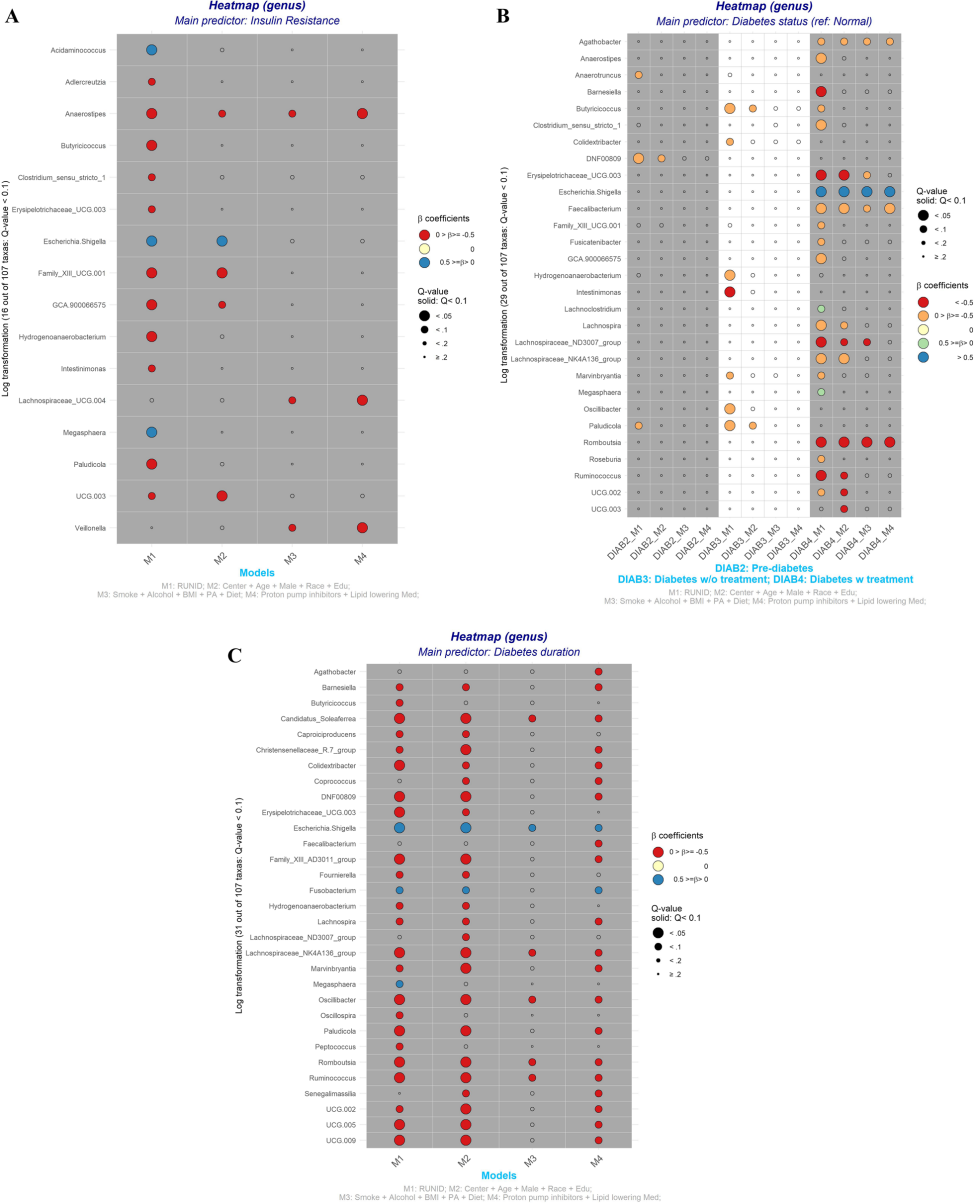
Heat maps show the associations of 107 gut microbial genus (log-transformed counts) with insulin resistance, stages of diabetes, and diabetes duration. **A** Homeostatic Model Assessment for Insulin Resistance (continuous); **B** Stages of diabetes (4 stages including normal, pre-diabetes, diabetes without treatment, and diabetes with treatment); **C** Diabetes duration (continuous). Data source: The Coronary Artery Risk Development in Young Adults (CARDIA), 1985–2016. Only genus with significant association (q-value < 0.1) in at least one of the 4 multivariable-adjusted linear regression models were displayed. The log-transformed genera counts were used for the analyses. Positive beta coefficients indicate greater abundance and vice versa. The q-value was calculated using the Benjamini–Hochberg method. Model 1 adjusted for sequencing run. Model 2 additionally adjusted for age, sex, race, field center, and education. Model 3 additionally adjusted for physical activity, smoking status, alcohol use, and diet quality score. In Model 4, medication use, such as proton pump inhibitor and lipid-lowering, was added

For taxa-specific analysis, we observed three genera (*Anaerostipes, Lachnospiraceae_UCG.004,* and *Veillonella*) inversely associated with HOMA-IR after adjusting for the complete set of covariates and multiple comparisons (Fig. 2A, M4). However, these associations became non-significant after adjusting for diabetes medication use (Additional file 1: Table S2a, M6).

### Diabetes stages and gut microbiota diversity and composition

Compared with normoglycemic participants, T2D + had lower genera Shannon diversity index (β [95% CI] =  − 0.3 [− 0.6, − 0.01]) and richness (β [95% CI] =  − 0.5 [− 0.06, − 0.02]) (Table 2, Model 4); while lower richness levels were found in pre-diabetes and T2D−, these associations were attenuated after adjusting for behavioral factors (Table 2, Model 3; Additional file 1: Table S4). There were significant differences in β diversity over the four stages of diabetes (Fig. 1B; Additional file 1: Table S3).

In the fully adjusted model, we observed three genera (*Agathobacter, Faecalibacterium,* and *Romboutsia*) significantly negatively associated with diabetes + (Fig. 2B, DIAB4_M4), compared with normoglycemic participants. The number and type of taxa significantly associated with pre-diabetes and diabetes was modulated after adjusting for behavioral factors. When we relaxed our FDR cut-off from < 0.1 to FDR < 0.2, with full adjustment, we found one genus (*Eggerthellaceae* [*DNF00809*]) (Fig. 2B, DIAB2) was associated with pre-diabetes and two genera (*Butyricicoccus* and *Colidextribacter*) were associated with diabetes (Fig. 2B, DIAB3). Further adjustment for SBP, DBP, and antihypertensive medication did not change the conclusions for α and β diversity or taxa abundance (Additional file 1: Table S4 and Additional file 1: Table S2c, Model 5).

### Diabetes duration and gut microbiota diversity and composition

Compared those with normoglycemic or prediabetes levels, we found duration (as continuous with 0 including normal and prediabetes) with the disease was negatively associated with both α diversity measures (βs [95% CIs] =  − 0.03 [− 0.05, − 0.005] and − 0.04 [− 0.06, − 0.02] for Shannon index and richness, respectively) (Table 2, Model 4), which was strongest for the comparison of normoglycemic/prediabetes group compared to those with diabetes ≥ 5 yrs (Additional file 1: Table S4). The association remained statistically significant after adjustment for SBP, DBP, and the use of antihypertensive and diabetes medications (Additional file 1: Table S3). PERMANOVA test showed differences in β diversity across diabetes duration groups (Fig. 1C).

After multivariable and FDR adjustment, we observed 22 genera were significantly associated (including two positive associations) with diabetes duration (Fig. 2C, M4). This finding survived adjustment for SBP, DBP, and antihypertensive medication (Additional file 1: Table S2b, Model 4 and 5), but was attenuated to not significant after further adjusting for diabetes medication use (Additional file 1: Table S2b, Model 6).

## Discussion

In this community-based study of middle-aged Black and White Americans, our findings indicate significant associations of diabetes duration and diabetes stages with microbial diversity measures, but in several analyses, conclusions were sensitive to adjustment for socio-demographics, health behaviors, clinical risk factors and use of diabetes-regulating medications. Compared to the normoglycemic group, we found insulin resistance and those with T2D had less unique genus of the gut microbiome (alpha diversity) and a different gut microbial composition (beta diversity), although the results of alpha diversity and HOMA-IR were attenuated with adjustment for covariates. We also found a lower abundance of butyrate-producing gut bacteria in T2D with longer duration, particularly in those who were receiving diabetes treatment, compared with the normoglycemic group.

Previous community-based studies have shown that gut microbiota diversity was associated with diabetes [11, 12]. Similar to our study, Wu et al. found significant differences in β diversity in pre-diabetes or newly diagnosed T2D (assuming diabetes-treatment-naïve in the study), compared with the normal glycemic group [11]. Our genera-specific results are generally consistent with findings from animal models and human studies [2, 5, 11, 12]. However, our results might not be directly comparable to previously published human studies due to the research design (i.e., case–control vs. cohort or cross-sectional, heterogeneity of race/ethnicity groups and health status), sample size of individuals with diabetes, the inclusion of persons with diabetes who were at different stages in the disease, and the inconsistent adjustment for potential confounders [2, 11, 12].

Our findings suggest there is a decrease in abundance of butyrate-producing gut bacteria among those with T2D+ (*Agathobacter* [28]*, Faecalibacterium* [29]*,* and *Romboutsia* [12]), but not between those with pre-diabetes and T2D−after adjusting for behavioral factors. When FDR was set to < 0.2, a few genera showed a lower abundance in pre-diabetes (*Eggerthellaceae* [*DNF00809*], not butyrate-producing gut bacteria) or diabetes without treatment (*Butyricicoccus* [30] and *Colidextribacter* [31]) relative to normoglycemia. Butyrate, is a short‐chain fatty acid that has been suggested to induce beneficial metabolic effects in both mice and humans [32–34], and lower levels of butyrate-producing bacteria have been linked to diabetes [11, 12]. It should be noted that the use of diabetes medication is associated with diabetes duration. In a sensitivity analysis, we found that adjusting for diabetes duration in diabetes stages model attenuated most of the significant association. Only *Romboutsia* and *Escherichia* remained statistically significant with diabetes + after adjustment for diabetes duration (Additional file 1: Table S2e, M6). Likewise, no genera remained statistically significant with diabetes duration after adjustment for diabetes treatment (Additional file 1: Table S2b, M6).

Previous studies have reported inconsistent results on the effect of diabetes medication on microbiota [2]. Taking antihyperglycemic medications improves human physiology, which may have potential positive effects on gut microbiota [2]. However, metformin has also been shown to adversely alter gut microbiome composition [9, 35], which is consistent with our finding of relatively higher abundance *Escherichia* among T2D+. We also found that two butyrate-producing bacteria (i.e., *Clostridium *sensu stricto* 1* [36] and *Lachnospria* [37]) showed lower abundance in T2D+, compared to T2D− (Additional file 1: Fig. S3). In addition, taking anti-hyperglycemic medications attenuated the associations of genus abundance with insulin resistance (*Anaerostipes*, *Lachnospiraceae* [*UCG.004*], and *Veillonella*) and diabetes duration (*Candidatus Soleaferrea*, *Colidextribacter*, *Lachnospiraceae* [*NK4A136* group], *Oscillibacter*, and *Romboutsia*) to non-significant. Anti-hypertension use also modulated diabetes duration results. This suggests that taking blood pressure or glycemic control medications may change the gut microbiota abundance induced by hyperglycemia [2, 16].

### Strengths

Our study has several strengths. CARDIA has collected objective assessment of diabetes-related characteristics variables and extensive covariates over 30 years using standardized protocols and validated methods. Therefore, we had an extensive medical history and could examine the impact of different sets of covariates on the gut microbiota with respect to these diabetes-related characteristics. In addition, a population-based, socio-demographically-diverse cohort strongly supports our findings’ external generalizability compared to clinical studies of patients. However, it should be noted that our sample was middle-aged and gut microbiota will change with age.

### Limitations

Even though the diabetes-related characteristics were measured over time, gut microbiota measures were based on a single stool sample collected at Y30, the latest available data in CARDIA. Therefore, we cannot study transitions from pre-diabetes to clinical diabetes in relation to shifts in microbiome, which would be an important follow-up to our findings. Cases of diabetes may be missed because we did not have available HbA1c or OGTT for the total sample. However, the diabetes status was ascertained by participant’s clinical measures and disease history over a 30-year follow-up period, which may minimize the misidentification issue. Likewise, we defined participants as having pre-diabetes who met ADA criteria at both the Y25 and the Y30 exams (Additional file 1: Fig. S1). This definition of pre-diabetes is more stable than one measure which does not account for the known probability of pre-diabetes reversing to normal glucose regulation [38]. Our cohort represents a relatively young population (mean age: 55.2 at the time of stool sample collected) in terms of T2D development [39]. Given the prevalence of diabetes increases with age, we will have more clinically heterogeneous diabetes patients and power to detect the associations between diabetes characteristics and microbiome composition in the next follow-up. We used 16S ribosomal RNA sequencing to evaluate changes in the gut microbiota composition and taxonomic differences at the genus level. Future analysis based on whole-metagenomics sequencing would enable the assessment of association of functional profiles of gut microbiome and taxonomic association at lower levels.

## Conclusions

In summary, our findings from a Black and White middle-aged population-based cohort showed significantly lower microbial diversity and butyrate-producing genera in those with treated diabetes and those with longer diabetes duration than in the normal glucose group. Our data are consistent with the hypothesis that the gut microbiome is linked to insulin resistance as well as an individual’s history of diabetes. However, the results are sensitive to lifestyle and risk factor levels, suggesting the gut microbiota is potentially modifiable through health behaviors. Our study also highlights the need to take health behavior differences into account when comparing studies based on diverse samples. We also show associations are modified by the use of medications, which is a long-recognized modifier of the gut microbiome [2]. Understanding the role of gut microbiota in glucose regulation may provide new strategies to reduce the burden of diabetes. Longitudinal studies are required to assess temporality of gut microbiota changes and the subsequent effect on glucose metabolism. Future studies of the microbiome and diabetes should consider the disease duration, and differences in associations within and across demographic sub-groups.

## Supplementary Information

**Additional file 1: Figure S1**. Research design. **Figure S2**. Sample flowchart. **Figure S3**. Heat maps show the associations of 107 gut microbial genus (log-transformed counts) with diabetes duration (using diabetes without treatment as the reference group). **Table S1**. Descriptive statistics of analytic and non-analytic sample—CARDIA cohort: Year 30 exam. **Table S2a**. The genus-level association between HOMA-IR and specific taxa (n = 604). **Table S2b**. The genus-level association between diabetes duration and specific taxa (n = 605). **Table S2c**. The genus-level association between stages of diabetes (normal vs. prediabetes) and specific taxa (n = 605, reference = normal). **Table S2d**. The genus-level association between stages of diabetes (normal vs. diabetes without treatment) and specific taxa (n = 605, reference = normal). **Table S2e**. The genus-level association between stages of diabetes (normal vs. diabetes with treatment) and specific taxa (n = 605, reference = normal). **Table S3**. Associations of gut microbial beta-diversity with insulin resistance, diabetes duration, and stages of diabetes. **Table S4**. Multivariable-adjusted associations of α diversity measures with insulin resistance, diabetes duration, and stages of diabetes

## Data Availability

The datasets used and/or analyzed during the current study are available from the corresponding author on reasonable request.

## Abbreviations

T2D: Type 2 diabetes
CARDIA: Coronary Artery Risk Development in Young Adults
HOMA-IR: Homeostatic Model Assessment of Insulin Resistance
ADA: American Diabetes Association
OGTT: Oral glucose tolerance test
FSG: Fasting serum glucose
2 h-PG: 2-Hour post-load glucose
HbA1c: Hemoglobin A1C
T2D+: Persons with type 2 diabetes who were on diabetes treatment
T2D−: Persons with type 2 diabetes not on diabetes treatment
BMI: Body mass index
SBP: Systolic blood pressure
DBP: Diastolic blood pressure
PERMANOVA: Permutational multivariate analysis of variance
PCoA: Principal coordinates analysis
CI: Confidence interval
SD: Standard deviation
FDR: False discovery rate
