# Supplementary material for: Gut microbiome and stages of diabetes in middle-aged adults: CARDIA microbiome study
Source: Nutr Metab (Lond). 2023 Jan 5;20:3. doi: 10.1186/s12986-022-00721-0 (PMC9817375; doi:10.1186/s12986-022-00721-0)
Supplement: Supplementary file 1 — Additional file 1: Figure S1. Research design. Figure S2. Sample flowchart. Figure S3. Heat maps show the associations of 107 gut microbial genus (log-transformed counts) with diabetes duration (using diabetes without treatment as the reference group). Table S1. Descriptive statistics of analytic and non-analytic sample—CARDIA cohort: Year 30 exam. Table S2a. The genus-level association between HOMA-IR and specific taxa (n = 604). Table S2b. The genus-level association between diabetes duration and specific taxa (n = 605). Table S2c. The genus-level association between stages of diabetes (normal vs. prediabetes) and specific taxa (n = 605, reference = normal). Table S2d. The genus-level association between stages of diabetes (normal vs. diabetes without treatment) and specific taxa (n = 605, reference = normal). Table S2e. The genus-level association between stages of diabetes (normal vs. diabetes with treatment) and specific taxa (n = 605, reference = normal). Table S3. Associations of gut microbial beta-diversity with insulin resistance, diabetes duration, and stages of diabetes. Table S4. Multivariable-adjusted associations of α diversity measures with insulin resistance, diabetes duration, and stages of diabetes [file 12986_2022_721_MOESM1_ESM.docx]

**Additional file 1**

**Title:** Gut Microbiome and Stages of Diabetes in Middle-aged Adults: CARDIA Microbiome Study

Yi-Han Hu, PhD,^1^ Katie Meyer, ScD^2,3^ Anju Lulla, PhD,^2^ Cora E. Lewis, MD, MSPH,^4^ Mercedes R Carnethon, PhD,^5^ Pamela J. Schreiner, PhD,^6^ Stephen Sidney, MD, MPH,^7^ James M Shikany, DrPH,^8^ Osorio Meirelles, PhD,^1^ Lenore J. Launer, PhD^1^

^1^ Laboratory of Epidemiology and Population Sciences, National Institute on Aging, Baltimore, MD, USA

^2^ Nutrition Research Institute, University of North Carolina at Chapel Hill, Kannapolis, NC, USA

^3^ Department of Nutrition, University of North Carolina at Chapel Hill, Chapel Hill, NC, USA

^4^ Department of Epidemiology, University of Alabama at Birmingham, Birmingham, AL, USA

^5^ Department of Preventive Medicine, Northwestern University, Feinberg School of Medicine, Chicago, IL, USA.

^6^ Division of Epidemiology and Community Health, University of Minnesota, Minneapolis, MN, USA

^7^ Kaiser Permanente Medical Center Program, Oakland, CA, USA

^8^ Division of Preventive Medicine, School of Medicine, University of Alabama at Birmingham, Birmingham, AL, USA

**Table of Contents**

Additional file Figure and Table 3

Additional file 1: Figure S1. Research design 3

Additional file 1: Figure S2. Sample flowchart 4

Additional file 1: Figure S3. Heat maps show the associations of 107 gut microbial genus (log-transformed counts) with diabetes duration (using diabetes without treatment as the reference group) 5

Additional file 1: Table S1. Descriptive statistics of analytic and non-analytic sample – CARDIA cohort: Year 30 exam^a^ 7

Additional file 1: Table S2a. The genus-level association between HOMA-IR and specific taxa (n = 604) 9

Additional file 1: Table S2b. The genus-level association between diabetes duration and specific taxa (n = 605) 16

Additional file 1: Table S2c. The genus-level association between stages of diabetes (normal vs. prediabetes) and specific taxa (n = 605, reference = normal) 23

Additional file 1: Table S2d. The genus-level association between stages of diabetes (normal vs. diabetes without treatment) and specific taxa (n = 605, reference = normal) 30

Additional file 1: Table S2e. The genus-level association between stages of diabetes (normal vs. diabetes with treatment) and specific taxa (n = 605, reference = normal) 37

Additional file 1: Table S3. Associations of gut microbial beta-diversity with insulin resistance, diabetes duration, and stages of diabetes^a,b^ 44

Additional file 1: Table S4. Multivariable-adjusted associations of α diversity measures with insulin resistance, diabetes duration, and stages of diabetes 46

Additional file Figure and Table

| 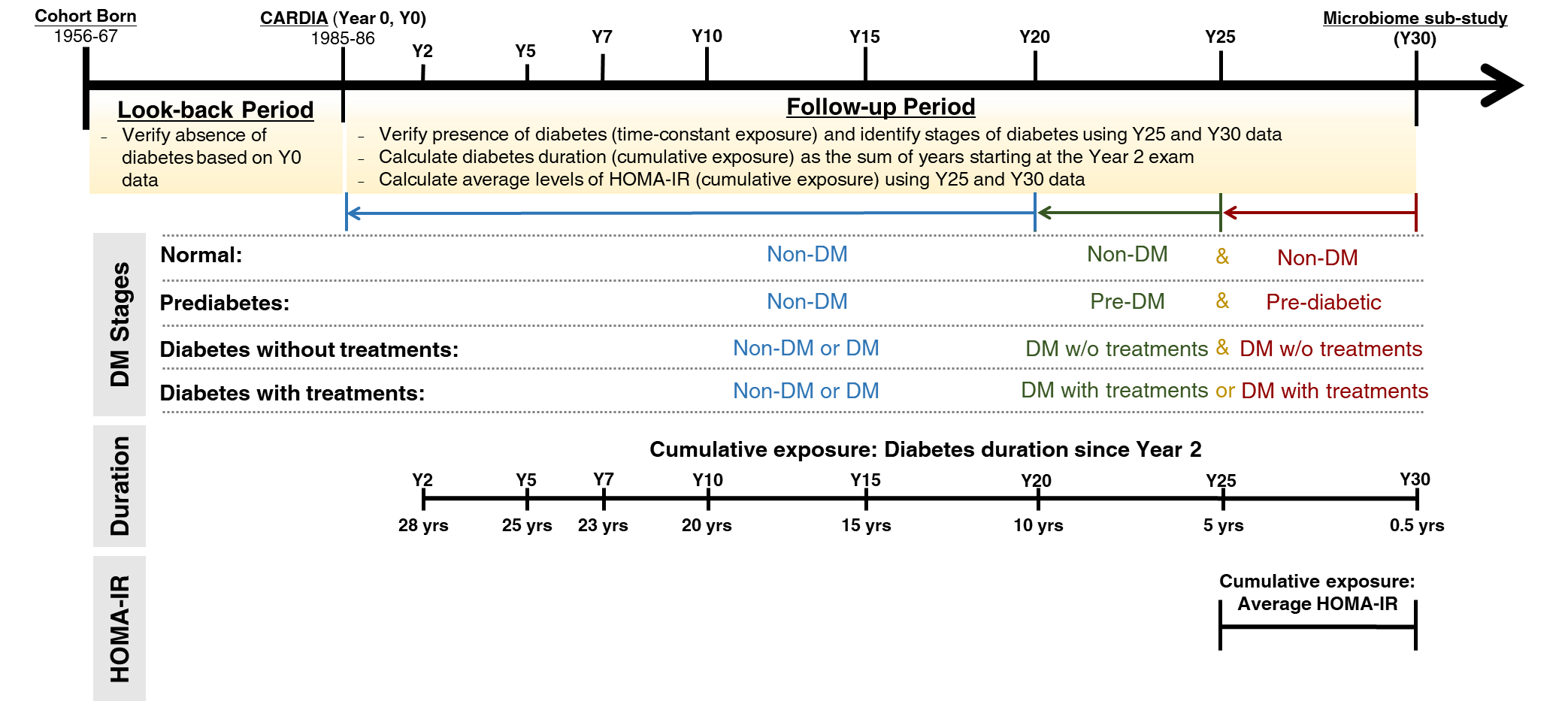 |
| --- |
| Additional file 1: Figure S1. Research design  ^1^ Diabetes was determined based on: a fasting serum glucose (FSG) level ≥126 mg/dL (available at Y0, Y7 and afterward), a 2-hour post-load glucose (2h-PG) ≥ 200 mg/dL during a 75-g oral glucose tolerance test (available at Y10, Y20 and Y25), a Hemoglobin A1C (HbA1c) ≥ 6.5% (available at Y20 and Y25), or self-report of diabetes medications (e.g., oral hypoglycemic medications or insulin) use.  ^2^ Prediabetes was defined based on: a FSG of 100-125 mg/dL, a 2h-PG of 140-199 mg/dL, or an HbA1c 5.7-6.4%.  ^3^ HOMA-IR: Homeostatic Model Assessment of Insulin Resistance was calculated as follow: [fasting insulin (uU/mL) × fasting glucose (mmol/L)]/22.5. |

| 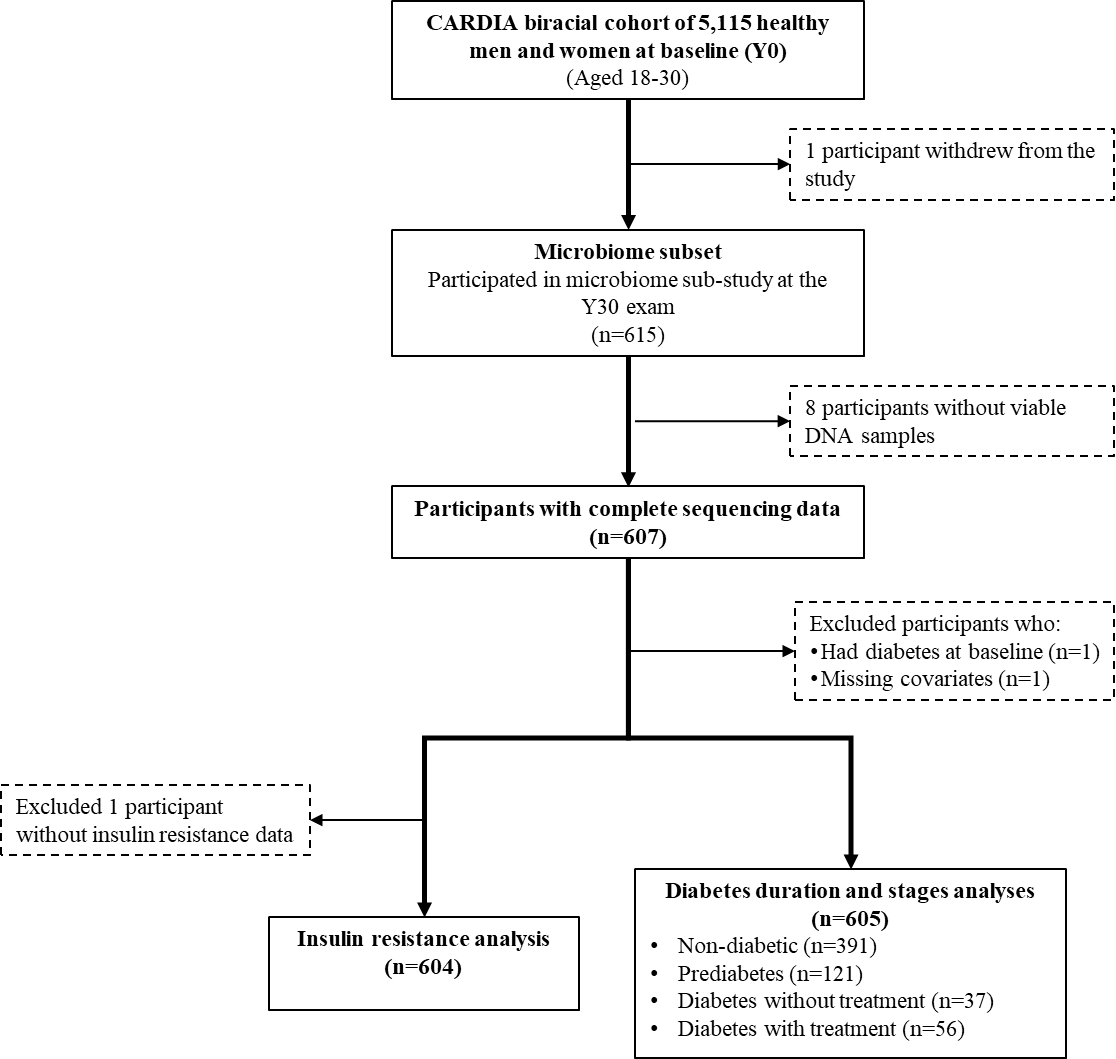 |
| --- |
| Additional file 1: Figure S2. Sample flowchart |

| 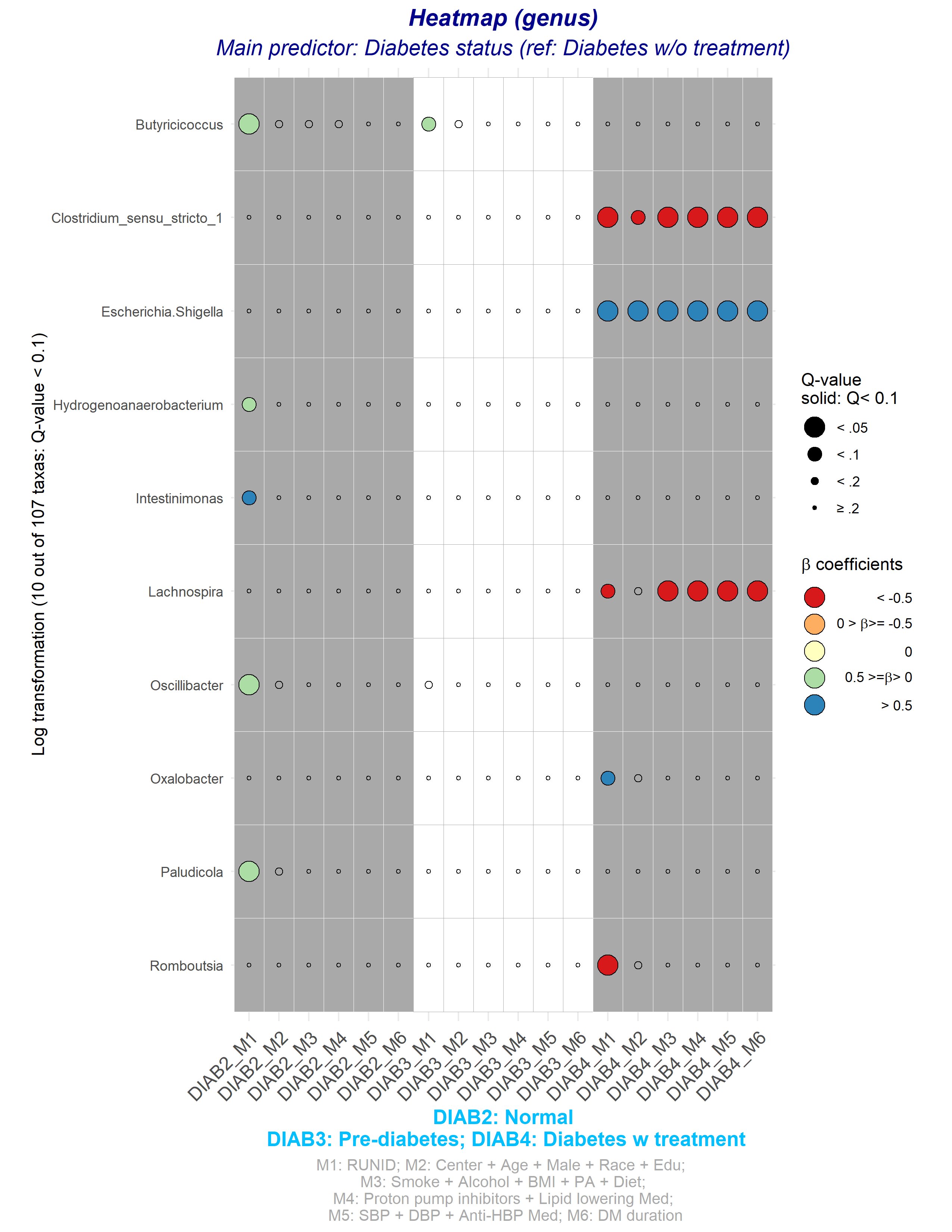 |
| --- |
| Additional file 1: Figure S3. Heat maps show the associations of 107 gut microbial genus (log-transformed counts) with diabetes duration (using diabetes without treatment as the reference group)  Data source: The Coronary Artery Risk Development in Young Adults (CARDIA), 1985-2016  Only genus with significant association (q-value < .1) in at least one of the 4 multivariable-adjusted linear regression models were displayed.  The log-transformed genera counts were used for the analyses. Positive beta coefficients indicate greater abundance and vice versa. The q-value was calculated using the Benjamini-Hochberg method.  Model 1 adjusted for sequencing run. Model 2 additionally adjusted for age, sex, race, field center, and education. Model 3 additionally adjusted for physical activity, smoking status, alcohol use, and diet quality score. In Model 4, medication use, such as proton pump inhibitor and lipid-lowering, was added. Model 5 included all covariates in Model 4 and added systolic blood pressure, diastolic blood pressure, and antihypertensive medication (Y/N). Model 6 included all covariates in Model 4 and further adjusted for diabetes duration. |

| Additional file 1: Table S1. Descriptive statistics of analytic and non-analytic sample – CARDIA cohort: Year 30 exam^a^ | | | | | |
| --- | --- | --- | --- | --- | --- |
|  | **Analytic sample** |  | **Non-analytic sample** |  | **p-value^e^** |
|  | (n=605) |  | (n=2,753) |  |  |
|  | n(%) / M(SD) |  | n(%) / M(SD) |  |  |
| **0.** **Enrolled in the microbiome study (%)** | 605 (100) |  | 2 (0.07) |  | N/A |
| **1. Diabetes-related variables** |  |  |  |  |  |
| **Fasting glucose (mg/dL)**^b^ | 100.2 (24.8) |  | 103.0 (33.1) |  | <.05 |
| Not available | 0 |  | 12 |  |  |
| **HOMA-Insurance resistance**^b^ | 2.9 (2.3) |  | 3.3 (3.2) |  | <.01 |
| Not available | 1 |  | 14 |  |  |
| **Diabetes duration, median (IQR)^c^** | 5 (5-10) |  | 5 (0.5-10) |  | 0.78 |
| Not available | 0 |  | 21 |  |  |
| **Stages of diabetes (%)** |  |  |  |  | 0.06 |
| Normal | 417 (68.9) |  | 1918 (69.7) |  |  |
| Prediabetes | 101 (16.7) |  | 363 (13.2) |  |  |
| Diabetes without treatment | 31 (5.1) |  | 144 (5.2) |  |  |
| Diabetes with treatment | 56 (9.3) |  | 328 (11.9) |  |  |
| **Metformin use (%)** | 43 (7.1) |  | 232 (8.4) |  | 0.32 |
| Not available | 0 |  | 1 |  |  |
| **2. Socio-demographics** |  |  |  |  |  |
| **Age**^b^ | 55.2 (3.5) |  | 55.1 (3.6) |  | 0.40 |
| **Male (%)** | 272 (45.0) |  | 1,172 (42.6) |  | 0.30 |
| **Black (%)** | 275 (45.5) |  | 1,330 (48.3) |  | 0.22 |
| **Highest education (%)** |  |  |  |  | <.05 |
| High school or less | 206 (34.1) |  | 1,095 (39.8) |  |  |
| College | 261 (43.1) |  | 1,087 (39.5) |  |  |
| Graduate school | 138 (22.8) |  | 571 (20.7) |  |  |
| **Field center (%)** |  |  |  |  | <.01 |
| Birmingham, AL | 98 (16.2) |  | 656 (23.8) |  |  |
| Chicago, IL | 295 (48.7) |  | 450 (16.4) |  |  |
| Minneapolis, MN | 110 (18.2) |  | 754 (27.4) |  |  |
| Oakland, CA | 102 (16.9) |  | 893 (32.4) |  |  |
| **3. Clinical measures**^b^ |  |  |  |  |  |
| **BMI** | 29.4 (6.2) |  | 30.8 (7.4) |  | <.01 |
| Not available | 0 |  | 1 |  |  |
| **Systolic blood pressure (mmHg)** | 119.3 (16.1) |  | 119.8 (16.0) |  | 0.48 |
| **Diastolic blood pressure (mmHg)** | 72.9 (11.0) |  | 73.2 (10.7) |  | 0.57 |
| **4. Health behavior** |  |  |  |  |  |
| **Current smoker (%)** | 81 (13.4) |  | 400 (14.6) |  | 0.49 |
| Not available | 0 |  | 10 |  |  |
| **Alcohol use (%)** | 469 (77.5) |  | 2,116 (77.4) |  | 0.98 |
| Not available | 0 |  | 18 |  |  |
| **Physical activity, median (IQR)**^d^ | 267 (128-504) |  | 253 (116-456) |  | <.05 |
| Not available | 0 |  | 18 |  |  |
| **5. Medication use (%)** |  |  |  |  |  |
| Proton pump inhibitor | 47 (7.8) |  | 227 (8.2) |  | 0.57 |
| Lipid-lowering | 123 (20.3) |  | 551 (20.1) |  | 0.92 |
| Not available | 0 |  | 6 |  |  |
| Anti-hypertensive | 180 (29.8) |  | 940 (34.2) |  | <.05 |
| Not available | 0 |  | 4 |  |  |
| Data source: The Coronary Artery Risk Development in Young Adults (CARDIA), 1985-2016  N/A = not applicable; HOMA-Insulin resistance = Homeostatic Model Assessment for Insulin Resistance; IQR = Interquartile range.  a: All covariates were collected at the Y30 exam. Missing covariates were updated using Y25 information.  b: Data is reported as mean and SD.  c: Statistics were calculated among participants with diabetes.  d: Total activity intensity in the past year.  e: p-value was based on the chi-square test for stages of diabetes, metformin use, male, race, education level, field center, smoking, alcohol use, or medication use; analysis of variance was used to estimate p-value for the remaining variables. | | | | | |

| Additional file 1: Table S2a. The genus-level association between HOMA-IR and specific taxa (n = 604) | | | | | | | | | | | | | | | | | | | | | | | | |
| --- | --- | --- | --- | --- | --- | --- | --- | --- | --- | --- | --- | --- | --- | --- | --- | --- | --- | --- | --- | --- | --- | --- | --- | --- |
|  | Model 1 | | |  | Model 2 | | |  | Model 3 | | |  | Model 4 | | |  | Model 5 | | |  | Model 6 | | | |
|  | β | p | FDR |  | β | p | FDR |  | β | p | FDR |  | β | p | FDR |  | β | p | FDR |  | β | p | FDR |  |
| Acetanaerobacterium | 0.003 | 0.71 | 0.81 |  | 0.01 | 0.44 | 0.73 |  | 0.02 | 0.10 | 0.36 |  | 0.02 | 0.10 | 0.35 |  | 0.02 | 0.10 | 0.33 |  | 0.02 | 0.05 | 0.34 |  |
| Acidaminococcus | 0.09 | 0.001 | 0.01 |  | 0.07 | 0.01 | 0.12 |  | 0.03 | 0.32 | 0.65 |  | 0.03 | 0.36 | 0.65 |  | 0.03 | 0.40 | 0.68 |  | 0.03 | 0.38 | 0.66 |  |
| Actinomyces | 0.02 | 0.03 | 0.15 |  | 0.01 | 0.22 | 0.51 |  | 0.003 | 0.82 | 0.91 |  | -0.002 | 0.85 | 0.90 |  | -0.002 | 0.85 | 0.92 |  | -0.002 | 0.86 | 0.94 |  |
| Adlercreutzia | -0.04 | 0.01 | 0.07 |  | -0.03 | 0.08 | 0.31 |  | -0.01 | 0.46 | 0.70 |  | -0.02 | 0.36 | 0.65 |  | -0.01 | 0.43 | 0.70 |  | -0.02 | 0.36 | 0.66 |  |
| Agathobacter | 0.01 | 0.48 | 0.67 |  | 0.003 | 0.81 | 0.94 |  | -0.01 | 0.50 | 0.70 |  | -0.01 | 0.51 | 0.73 |  | -0.01 | 0.50 | 0.71 |  | 0.004 | 0.81 | 0.93 |  |
| Akkermansia | -0.01 | 0.75 | 0.83 |  | 0.02 | 0.36 | 0.63 |  | 0.04 | 0.17 | 0.48 |  | 0.05 | 0.12 | 0.35 |  | 0.05 | 0.08 | 0.33 |  | 0.05 | 0.10 | 0.41 |  |
| Alistipes | -0.02 | 0.13 | 0.31 |  | -0.02 | 0.27 | 0.57 |  | -0.003 | 0.86 | 0.91 |  | -0.004 | 0.80 | 0.89 |  | -0.004 | 0.81 | 0.90 |  | -0.004 | 0.85 | 0.94 |  |
| Allisonella | 0.01 | 0.32 | 0.55 |  | -0.005 | 0.71 | 0.89 |  | -0.01 | 0.38 | 0.65 |  | -0.01 | 0.32 | 0.64 |  | -0.02 | 0.31 | 0.63 |  | -0.02 | 0.22 | 0.63 |  |
| Anaerofilum | -0.01 | 0.29 | 0.52 |  | -0.01 | 0.61 | 0.80 |  | 0.01 | 0.26 | 0.61 |  | 0.01 | 0.26 | 0.61 |  | 0.01 | 0.29 | 0.62 |  | 0.02 | 0.17 | 0.57 |  |
| Anaerostipes | -0.04 | <.001 | 0.004 |  | -0.03 | 0.002 | 0.05 |  | -0.04 | 0.001 | 0.07 |  | -0.04 | 0.001 | 0.04 |  | -0.04 | 0.002 | 0.07 |  | -0.04 | 0.01 | 0.18 |  |
| Anaerotruncus | -0.01 | 0.47 | 0.67 |  | -0.002 | 0.85 | 0.94 |  | 0.03 | 0.07 | 0.31 |  | 0.02 | 0.09 | 0.35 |  | 0.03 | 0.08 | 0.33 |  | 0.02 | 0.14 | 0.51 |  |
| Bacteroides | -0.01 | 0.14 | 0.31 |  | -0.005 | 0.51 | 0.75 |  | -0.02 | 0.06 | 0.29 |  | -0.02 | 0.07 | 0.32 |  | -0.02 | 0.06 | 0.28 |  | -0.02 | 0.04 | 0.34 |  |
| Barnesiella | -0.05 | 0.05 | 0.19 |  | -0.04 | 0.10 | 0.32 |  | -0.03 | 0.36 | 0.65 |  | -0.03 | 0.33 | 0.65 |  | -0.03 | 0.35 | 0.67 |  | -0.01 | 0.67 | 0.85 |  |
| Bifidobacterium | 0.03 | 0.09 | 0.26 |  | 0.03 | 0.17 | 0.44 |  | 0.01 | 0.56 | 0.77 |  | 0.01 | 0.62 | 0.82 |  | 0.01 | 0.67 | 0.85 |  | 0.03 | 0.31 | 0.63 |  |
| Bilophila | -0.01 | 0.54 | 0.69 |  | -0.01 | 0.49 | 0.74 |  | -0.02 | 0.42 | 0.66 |  | -0.02 | 0.39 | 0.66 |  | -0.02 | 0.35 | 0.67 |  | -0.02 | 0.41 | 0.67 |  |
| Blautia | -0.003 | 0.54 | 0.69 |  | 0.0003 | 0.96 | 0.97 |  | 0.001 | 0.83 | 0.91 |  | 0.001 | 0.93 | 0.94 |  | 0.001 | 0.90 | 0.94 |  | 0.0002 | 0.98 | 0.98 |  |
| Butyricicoccus | -0.02 | 0.004 | 0.05 |  | -0.02 | 0.04 | 0.22 |  | -0.02 | 0.05 | 0.25 |  | -0.02 | 0.06 | 0.32 |  | -0.02 | 0.08 | 0.33 |  | -0.02 | 0.12 | 0.48 |  |
| Butyricimonas | 0.01 | 0.52 | 0.69 |  | 0.002 | 0.91 | 0.95 |  | 0.01 | 0.75 | 0.88 |  | 0.01 | 0.85 | 0.90 |  | 0.01 | 0.79 | 0.90 |  | 0.001 | 0.98 | 0.98 |  |
| CAG.56 | -0.03 | 0.18 | 0.37 |  | -0.02 | 0.25 | 0.56 |  | -0.02 | 0.30 | 0.63 |  | -0.03 | 0.26 | 0.61 |  | -0.03 | 0.24 | 0.58 |  | -0.03 | 0.30 | 0.63 |  |
| Candidatus_Soleaferrea | -0.004 | 0.67 | 0.79 |  | 0.002 | 0.80 | 0.94 |  | 0.01 | 0.43 | 0.67 |  | 0.01 | 0.41 | 0.66 |  | 0.01 | 0.37 | 0.67 |  | 0.01 | 0.27 | 0.63 |  |
| Caproiciproducens | -0.0003 | 0.98 | 0.98 |  | 0.01 | 0.62 | 0.80 |  | 0.02 | 0.23 | 0.58 |  | 0.02 | 0.24 | 0.61 |  | 0.02 | 0.19 | 0.50 |  | 0.02 | 0.14 | 0.51 |  |
| Christensenellaceae_R.7_group | -0.05 | 0.02 | 0.11 |  | -0.05 | 0.03 | 0.18 |  | 0.002 | 0.94 | 0.96 |  | 0.001 | 0.96 | 0.97 |  | 0.01 | 0.83 | 0.90 |  | 0.01 | 0.61 | 0.82 |  |
| Clostridium_sensu_stricto_1 | -0.05 | 0.01 | 0.08 |  | -0.04 | 0.02 | 0.18 |  | -0.06 | 0.01 | 0.12 |  | -0.05 | 0.01 | 0.17 |  | -0.06 | 0.01 | 0.17 |  | -0.04 | 0.06 | 0.34 |  |
| Colidextribacter | -0.001 | 0.92 | 0.97 |  | 0.01 | 0.56 | 0.78 |  | 0.02 | 0.13 | 0.40 |  | 0.02 | 0.16 | 0.46 |  | 0.01 | 0.18 | 0.48 |  | 0.02 | 0.09 | 0.41 |  |
| Collinsella | 0.03 | 0.20 | 0.41 |  | 0.02 | 0.45 | 0.73 |  | -0.01 | 0.75 | 0.88 |  | -0.01 | 0.74 | 0.88 |  | -0.01 | 0.72 | 0.87 |  | -0.001 | 0.97 | 0.98 |  |
| Coprobacter | -0.04 | 0.02 | 0.14 |  | -0.04 | 0.05 | 0.22 |  | -0.02 | 0.51 | 0.70 |  | -0.01 | 0.58 | 0.79 |  | -0.01 | 0.67 | 0.85 |  | -0.01 | 0.69 | 0.86 |  |
| Coprococcus | -0.01 | 0.40 | 0.65 |  | -0.02 | 0.27 | 0.57 |  | -0.02 | 0.33 | 0.65 |  | -0.02 | 0.36 | 0.65 |  | -0.02 | 0.34 | 0.67 |  | -0.01 | 0.58 | 0.82 |  |
| Defluviitaleaceae_UCG.011 | -0.01 | 0.60 | 0.72 |  | 0.01 | 0.56 | 0.78 |  | 0.04 | 0.01 | 0.12 |  | 0.04 | 0.01 | 0.16 |  | 0.04 | 0.004 | 0.10 |  | 0.04 | 0.01 | 0.18 |  |
| Desulfovibrio | 0.01 | 0.80 | 0.87 |  | -0.01 | 0.51 | 0.75 |  | 0.03 | 0.29 | 0.63 |  | 0.02 | 0.36 | 0.65 |  | 0.03 | 0.27 | 0.61 |  | 0.01 | 0.60 | 0.82 |  |
| Dialister | 0.004 | 0.87 | 0.93 |  | 0.01 | 0.67 | 0.85 |  | -0.01 | 0.65 | 0.83 |  | -0.02 | 0.62 | 0.82 |  | -0.02 | 0.51 | 0.71 |  | -0.01 | 0.72 | 0.88 |  |
| DNF00809 | -0.02 | 0.10 | 0.27 |  | -0.01 | 0.43 | 0.72 |  | 0.01 | 0.47 | 0.70 |  | 0.01 | 0.50 | 0.72 |  | 0.01 | 0.44 | 0.70 |  | 0.01 | 0.38 | 0.66 |  |
| Dorea | -0.01 | 0.45 | 0.67 |  | -0.01 | 0.31 | 0.62 |  | -0.03 | 0.07 | 0.31 |  | -0.03 | 0.07 | 0.32 |  | -0.03 | 0.06 | 0.28 |  | -0.03 | 0.05 | 0.34 |  |
| DTU089 | -0.001 | 0.93 | 0.97 |  | 0.01 | 0.47 | 0.74 |  | 0.03 | 0.06 | 0.29 |  | 0.03 | 0.05 | 0.32 |  | 0.03 | 0.05 | 0.28 |  | 0.03 | 0.07 | 0.34 |  |
| Eggerthella | -0.001 | 0.97 | 0.98 |  | 0.01 | 0.54 | 0.78 |  | 0.02 | 0.29 | 0.63 |  | 0.02 | 0.27 | 0.61 |  | 0.02 | 0.32 | 0.64 |  | 0.02 | 0.32 | 0.63 |  |
| Eisenbergiella | -0.001 | 0.94 | 0.97 |  | 0.01 | 0.59 | 0.80 |  | 0.04 | 0.01 | 0.12 |  | 0.04 | 0.02 | 0.17 |  | 0.04 | 0.02 | 0.18 |  | 0.04 | 0.02 | 0.30 |  |
| Erysipelatoclostridium | -0.01 | 0.43 | 0.66 |  | -0.002 | 0.89 | 0.95 |  | 0.01 | 0.34 | 0.65 |  | 0.02 | 0.29 | 0.63 |  | 0.02 | 0.29 | 0.62 |  | 0.02 | 0.23 | 0.63 |  |
| Erysipelotrichaceae_UCG.003 | -0.05 | 0.01 | 0.07 |  | -0.03 | 0.07 | 0.26 |  | -0.02 | 0.47 | 0.70 |  | -0.01 | 0.69 | 0.87 |  | -0.01 | 0.77 | 0.90 |  | 0.01 | 0.71 | 0.88 |  |
| Escherichia.Shigella | 0.08 | <.001 | 0.003 |  | 0.07 | <.001 | 0.01 |  | 0.06 | 0.01 | 0.12 |  | 0.06 | 0.01 | 0.17 |  | 0.05 | 0.02 | 0.18 |  | 0.03 | 0.20 | 0.61 |  |
| Faecalibacterium | -0.02 | 0.06 | 0.20 |  | -0.03 | 0.02 | 0.18 |  | -0.03 | 0.05 | 0.25 |  | -0.03 | 0.03 | 0.22 |  | -0.03 | 0.04 | 0.26 |  | -0.02 | 0.25 | 0.63 |  |
| Family_XIII_AD3011_group | -0.03 | 0.02 | 0.11 |  | -0.03 | 0.04 | 0.22 |  | 0.01 | 0.69 | 0.85 |  | 0.01 | 0.74 | 0.88 |  | 0.01 | 0.61 | 0.79 |  | 0.01 | 0.50 | 0.77 |  |
| Family_XIII_UCG.001 | -0.05 | <.001 | 0.003 |  | -0.04 | <.001 | 0.02 |  | -0.03 | 0.04 | 0.25 |  | -0.03 | 0.04 | 0.24 |  | -0.03 | 0.06 | 0.28 |  | -0.03 | 0.07 | 0.34 |  |
| FD2005 | 0.01 | 0.47 | 0.67 |  | 0.003 | 0.82 | 0.94 |  | -0.004 | 0.81 | 0.91 |  | -0.003 | 0.82 | 0.90 |  | -0.004 | 0.78 | 0.90 |  | -0.004 | 0.81 | 0.93 |  |
| Flavonifractor | 0.01 | 0.61 | 0.72 |  | 0.02 | 0.22 | 0.51 |  | 0.02 | 0.30 | 0.63 |  | 0.02 | 0.30 | 0.64 |  | 0.01 | 0.40 | 0.68 |  | 0.02 | 0.29 | 0.63 |  |
| Fournierella | -0.02 | 0.04 | 0.17 |  | -0.03 | 0.02 | 0.18 |  | -0.02 | 0.14 | 0.42 |  | -0.02 | 0.11 | 0.35 |  | -0.02 | 0.16 | 0.45 |  | -0.02 | 0.25 | 0.63 |  |
| Frisingicoccus | -0.01 | 0.71 | 0.81 |  | 0.01 | 0.66 | 0.85 |  | 0.04 | 0.06 | 0.29 |  | 0.03 | 0.11 | 0.35 |  | 0.03 | 0.09 | 0.33 |  | 0.03 | 0.19 | 0.60 |  |
| Fusicatenibacter | 0.005 | 0.79 | 0.87 |  | 0.01 | 0.73 | 0.89 |  | -0.01 | 0.79 | 0.90 |  | -0.003 | 0.89 | 0.93 |  | -0.003 | 0.90 | 0.94 |  | 0.01 | 0.57 | 0.82 |  |
| Fusobacterium | 0.005 | 0.69 | 0.80 |  | 0.001 | 0.95 | 0.97 |  | 0.001 | 0.96 | 0.96 |  | 0.0004 | 0.98 | 0.98 |  | -0.001 | 0.93 | 0.95 |  | -0.01 | 0.58 | 0.82 |  |
| GCA.900066575 | -0.04 | <.001 | 0.01 |  | -0.04 | 0.003 | 0.06 |  | -0.01 | 0.38 | 0.65 |  | -0.01 | 0.47 | 0.70 |  | -0.01 | 0.47 | 0.71 |  | -0.002 | 0.89 | 0.96 |  |
| Gordonibacter | -0.02 | 0.15 | 0.33 |  | -0.002 | 0.90 | 0.95 |  | 0.03 | 0.03 | 0.25 |  | 0.03 | 0.02 | 0.19 |  | 0.04 | 0.02 | 0.18 |  | 0.03 | 0.03 | 0.34 |  |
| Granulicatella | 0.01 | 0.54 | 0.69 |  | -0.0001 | 0.99 | 0.99 |  | -0.01 | 0.47 | 0.70 |  | -0.01 | 0.31 | 0.64 |  | -0.01 | 0.29 | 0.62 |  | -0.01 | 0.33 | 0.63 |  |
| Haemophilus | -0.03 | 0.05 | 0.19 |  | -0.04 | 0.02 | 0.18 |  | -0.05 | 0.02 | 0.21 |  | -0.06 | 0.01 | 0.16 |  | -0.05 | 0.01 | 0.15 |  | -0.05 | 0.02 | 0.30 |  |
| Harryflintia | 0.02 | 0.13 | 0.31 |  | 0.02 | 0.09 | 0.32 |  | 0.03 | 0.02 | 0.21 |  | 0.03 | 0.02 | 0.19 |  | 0.03 | 0.02 | 0.20 |  | 0.03 | 0.04 | 0.34 |  |
| Holdemanella | 0.03 | 0.05 | 0.19 |  | 0.02 | 0.36 | 0.63 |  | 0.01 | 0.49 | 0.70 |  | 0.01 | 0.53 | 0.75 |  | 0.01 | 0.56 | 0.76 |  | 0.01 | 0.64 | 0.84 |  |
| Holdemania | -0.01 | 0.08 | 0.24 |  | -0.01 | 0.34 | 0.63 |  | 0.004 | 0.62 | 0.83 |  | 0.01 | 0.55 | 0.76 |  | 0.01 | 0.55 | 0.76 |  | 0.01 | 0.38 | 0.66 |  |
| Hungatella | 0.01 | 0.37 | 0.62 |  | 0.02 | 0.15 | 0.40 |  | 0.01 | 0.40 | 0.65 |  | 0.01 | 0.46 | 0.70 |  | 0.01 | 0.49 | 0.71 |  | 0.01 | 0.73 | 0.88 |  |
| Hydrogenoanaerobacterium | -0.04 | 0.004 | 0.05 |  | -0.03 | 0.02 | 0.18 |  | -0.01 | 0.65 | 0.83 |  | -0.01 | 0.68 | 0.87 |  | -0.004 | 0.81 | 0.90 |  | -0.003 | 0.84 | 0.94 |  |
| Incertae_Sedis | 0.01 | 0.14 | 0.32 |  | 0.02 | 0.06 | 0.26 |  | 0.02 | 0.01 | 0.14 |  | 0.02 | 0.02 | 0.17 |  | 0.02 | 0.02 | 0.18 |  | 0.02 | 0.02 | 0.30 |  |
| Intestinibacter | -0.01 | 0.52 | 0.69 |  | -0.002 | 0.86 | 0.94 |  | -0.04 | 0.04 | 0.25 |  | -0.04 | 0.04 | 0.26 |  | -0.03 | 0.05 | 0.28 |  | -0.03 | 0.13 | 0.48 |  |
| Intestinimonas | -0.04 | 0.01 | 0.10 |  | -0.02 | 0.10 | 0.32 |  | 0.002 | 0.89 | 0.92 |  | 0.004 | 0.81 | 0.89 |  | 0.01 | 0.76 | 0.90 |  | -0.001 | 0.95 | 0.98 |  |
| Lachnoclostridium | 0.02 | 0.01 | 0.10 |  | 0.02 | 0.03 | 0.18 |  | 0.002 | 0.86 | 0.91 |  | 0.001 | 0.91 | 0.94 |  | -0.0005 | 0.95 | 0.96 |  | -0.003 | 0.75 | 0.89 |  |
| Lachnospira | -0.02 | 0.25 | 0.49 |  | -0.01 | 0.47 | 0.74 |  | -0.02 | 0.35 | 0.65 |  | -0.02 | 0.35 | 0.65 |  | -0.01 | 0.44 | 0.70 |  | -0.001 | 0.96 | 0.98 |  |
| Lachnospiraceae_FCS020_group | -0.03 | 0.03 | 0.15 |  | -0.03 | 0.03 | 0.18 |  | -0.03 | 0.10 | 0.36 |  | -0.03 | 0.10 | 0.35 |  | -0.03 | 0.11 | 0.35 |  | -0.02 | 0.20 | 0.61 |  |
| Lachnospiraceae_ND3007_group | -0.02 | 0.46 | 0.67 |  | -0.02 | 0.47 | 0.74 |  | -0.03 | 0.18 | 0.48 |  | -0.03 | 0.23 | 0.59 |  | -0.03 | 0.24 | 0.58 |  | -0.01 | 0.63 | 0.84 |  |
| Lachnospiraceae_NK4A136_group | -0.03 | 0.05 | 0.19 |  | -0.02 | 0.13 | 0.36 |  | 0.003 | 0.87 | 0.91 |  | 0.01 | 0.75 | 0.88 |  | 0.01 | 0.69 | 0.87 |  | 0.02 | 0.29 | 0.63 |  |
| Lachnospiraceae_UCG.001 | -0.03 | 0.07 | 0.21 |  | -0.03 | 0.10 | 0.32 |  | -0.02 | 0.49 | 0.70 |  | -0.02 | 0.43 | 0.68 |  | -0.02 | 0.45 | 0.70 |  | -0.01 | 0.72 | 0.88 |  |
| Lachnospiraceae_UCG.004 | -0.04 | 0.02 | 0.14 |  | -0.05 | 0.01 | 0.12 |  | -0.07 | 0.001 | 0.07 |  | -0.07 | 0.001 | 0.04 |  | -0.07 | 0.001 | 0.05 |  | -0.07 | 0.001 | 0.13 |  |
| Lachnospiraceae_UCG.010 | -0.01 | 0.42 | 0.66 |  | -0.01 | 0.27 | 0.57 |  | 0.003 | 0.83 | 0.91 |  | 0.004 | 0.78 | 0.89 |  | 0.003 | 0.86 | 0.92 |  | 0.01 | 0.61 | 0.82 |  |
| Lactobacillus | 0.02 | 0.14 | 0.31 |  | 0.01 | 0.35 | 0.63 |  | -0.02 | 0.22 | 0.56 |  | -0.03 | 0.09 | 0.35 |  | -0.03 | 0.10 | 0.33 |  | -0.03 | 0.16 | 0.55 |  |
| Lactococcus | -0.01 | 0.41 | 0.66 |  | -0.01 | 0.51 | 0.75 |  | -0.01 | 0.71 | 0.86 |  | -0.01 | 0.55 | 0.76 |  | -0.01 | 0.49 | 0.71 |  | -0.01 | 0.65 | 0.84 |  |
| Marvinbryantia | -0.04 | 0.04 | 0.17 |  | -0.04 | 0.05 | 0.22 |  | -0.03 | 0.17 | 0.48 |  | -0.03 | 0.18 | 0.51 |  | -0.03 | 0.18 | 0.48 |  | -0.02 | 0.41 | 0.67 |  |
| Megasphaera | 0.07 | 0.001 | 0.02 |  | 0.04 | 0.05 | 0.22 |  | 0.01 | 0.60 | 0.81 |  | 0.01 | 0.74 | 0.88 |  | 0.004 | 0.87 | 0.92 |  | 0.001 | 0.96 | 0.98 |  |
| Monoglobus | -0.01 | 0.48 | 0.67 |  | 0.005 | 0.74 | 0.89 |  | 0.03 | 0.13 | 0.40 |  | 0.03 | 0.11 | 0.35 |  | 0.03 | 0.10 | 0.33 |  | 0.03 | 0.06 | 0.34 |  |
| Moryella | -0.02 | 0.09 | 0.26 |  | -0.02 | 0.11 | 0.32 |  | -0.01 | 0.37 | 0.65 |  | -0.01 | 0.47 | 0.70 |  | -0.01 | 0.48 | 0.71 |  | -0.01 | 0.58 | 0.82 |  |
| Negativibacillus | 0.03 | 0.13 | 0.31 |  | 0.03 | 0.11 | 0.33 |  | 0.04 | 0.04 | 0.25 |  | 0.04 | 0.08 | 0.35 |  | 0.04 | 0.09 | 0.33 |  | 0.04 | 0.08 | 0.36 |  |
| NK4A214_group | -0.05 | 0.03 | 0.15 |  | -0.05 | 0.03 | 0.18 |  | -0.01 | 0.63 | 0.83 |  | -0.02 | 0.48 | 0.71 |  | -0.02 | 0.56 | 0.76 |  | -0.01 | 0.76 | 0.89 |  |
| Odoribacter | -0.03 | 0.16 | 0.34 |  | -0.04 | 0.10 | 0.32 |  | -0.01 | 0.77 | 0.89 |  | -0.01 | 0.80 | 0.89 |  | -0.01 | 0.79 | 0.90 |  | -0.02 | 0.55 | 0.82 |  |
| Oscillibacter | -0.03 | 0.03 | 0.15 |  | -0.02 | 0.22 | 0.51 |  | 0.01 | 0.66 | 0.83 |  | 0.01 | 0.69 | 0.87 |  | 0.01 | 0.70 | 0.87 |  | 0.01 | 0.50 | 0.77 |  |
| Oscillospira | -0.01 | 0.46 | 0.67 |  | 0.003 | 0.72 | 0.89 |  | 0.01 | 0.40 | 0.65 |  | 0.01 | 0.41 | 0.66 |  | 0.01 | 0.44 | 0.70 |  | 0.01 | 0.36 | 0.66 |  |
| Oxalobacter | 0.01 | 0.57 | 0.72 |  | 0.01 | 0.52 | 0.75 |  | 0.03 | 0.08 | 0.32 |  | 0.03 | 0.10 | 0.35 |  | 0.03 | 0.06 | 0.28 |  | 0.02 | 0.26 | 0.63 |  |
| Paludicola | -0.05 | <.001 | 0.004 |  | -0.03 | 0.01 | 0.12 |  | -0.001 | 0.96 | 0.96 |  | -0.003 | 0.85 | 0.90 |  | -0.00004 | 1.00 | 1.00 |  | -0.003 | 0.85 | 0.94 |  |
| Parabacteroides | -0.03 | 0.12 | 0.31 |  | -0.03 | 0.14 | 0.39 |  | -0.04 | 0.04 | 0.25 |  | -0.05 | 0.03 | 0.22 |  | -0.05 | 0.03 | 0.22 |  | -0.05 | 0.04 | 0.34 |  |
| Paraprevotella | 0.02 | 0.49 | 0.67 |  | 0.001 | 0.95 | 0.97 |  | 0.03 | 0.30 | 0.63 |  | 0.03 | 0.38 | 0.66 |  | 0.02 | 0.46 | 0.70 |  | 0.03 | 0.32 | 0.63 |  |
| Parasutterella | -0.03 | 0.24 | 0.46 |  | -0.03 | 0.28 | 0.58 |  | -0.04 | 0.21 | 0.56 |  | -0.03 | 0.27 | 0.61 |  | -0.03 | 0.37 | 0.67 |  | -0.03 | 0.28 | 0.63 |  |
| Peptococcus | -0.01 | 0.27 | 0.52 |  | -0.01 | 0.34 | 0.63 |  | 0.01 | 0.65 | 0.83 |  | 0.01 | 0.63 | 0.82 |  | 0.01 | 0.60 | 0.79 |  | 0.01 | 0.43 | 0.69 |  |
| Phascolarctobacterium | -0.01 | 0.81 | 0.88 |  | -0.01 | 0.60 | 0.80 |  | 0.03 | 0.30 | 0.63 |  | 0.03 | 0.32 | 0.64 |  | 0.04 | 0.24 | 0.58 |  | 0.03 | 0.28 | 0.63 |  |
| Phocea | 0.01 | 0.30 | 0.52 |  | 0.01 | 0.34 | 0.63 |  | 0.002 | 0.87 | 0.91 |  | 0.002 | 0.84 | 0.90 |  | 0.001 | 0.93 | 0.95 |  | 0.01 | 0.65 | 0.84 |  |
| Prevotella | 0.03 | 0.30 | 0.52 |  | 0.003 | 0.91 | 0.95 |  | 0.01 | 0.72 | 0.86 |  | 0.01 | 0.80 | 0.89 |  | 0.01 | 0.76 | 0.90 |  | 0.003 | 0.94 | 0.98 |  |
| Romboutsia | -0.03 | 0.06 | 0.19 |  | -0.02 | 0.12 | 0.36 |  | -0.04 | 0.04 | 0.25 |  | -0.03 | 0.06 | 0.32 |  | -0.04 | 0.06 | 0.28 |  | -0.01 | 0.52 | 0.78 |  |
| Roseburia | -0.02 | 0.13 | 0.31 |  | -0.02 | 0.24 | 0.55 |  | -0.03 | 0.09 | 0.33 |  | -0.03 | 0.09 | 0.35 |  | -0.03 | 0.11 | 0.35 |  | -0.02 | 0.27 | 0.63 |  |
| Ruminococcus | -0.02 | 0.44 | 0.67 |  | -0.004 | 0.85 | 0.94 |  | 0.03 | 0.25 | 0.60 |  | 0.03 | 0.24 | 0.60 |  | 0.03 | 0.17 | 0.48 |  | 0.05 | 0.05 | 0.34 |  |
| Sellimonas | -0.02 | 0.29 | 0.52 |  | -0.01 | 0.34 | 0.63 |  | 0.02 | 0.34 | 0.65 |  | 0.02 | 0.36 | 0.65 |  | 0.02 | 0.37 | 0.67 |  | 0.02 | 0.24 | 0.63 |  |
| Senegalimassilia | 0.01 | 0.54 | 0.69 |  | -0.004 | 0.80 | 0.94 |  | -0.003 | 0.87 | 0.91 |  | -0.005 | 0.80 | 0.89 |  | -0.004 | 0.81 | 0.90 |  | -0.001 | 0.97 | 0.98 |  |
| Shuttleworthia | -0.02 | 0.21 | 0.43 |  | 0.002 | 0.86 | 0.94 |  | 0.03 | 0.04 | 0.25 |  | 0.03 | 0.06 | 0.32 |  | 0.03 | 0.06 | 0.28 |  | 0.04 | 0.03 | 0.34 |  |
| Slackia | 0.01 | 0.58 | 0.72 |  | -0.01 | 0.60 | 0.80 |  | -0.02 | 0.42 | 0.66 |  | -0.02 | 0.44 | 0.68 |  | -0.02 | 0.46 | 0.70 |  | -0.02 | 0.41 | 0.67 |  |
| Streptococcus | -0.01 | 0.40 | 0.65 |  | -0.02 | 0.20 | 0.50 |  | -0.02 | 0.12 | 0.40 |  | -0.03 | 0.02 | 0.17 |  | -0.03 | 0.02 | 0.18 |  | -0.03 | 0.06 | 0.34 |  |
| Subdoligranulum | -0.03 | 0.05 | 0.19 |  | -0.03 | 0.05 | 0.22 |  | -0.02 | 0.39 | 0.65 |  | -0.02 | 0.39 | 0.66 |  | -0.02 | 0.38 | 0.67 |  | -0.02 | 0.40 | 0.67 |  |
| Sutterella | -0.03 | 0.29 | 0.52 |  | -0.05 | 0.07 | 0.26 |  | -0.05 | 0.12 | 0.40 |  | -0.04 | 0.19 | 0.52 |  | -0.05 | 0.15 | 0.45 |  | -0.03 | 0.32 | 0.63 |  |
| TM7x | -0.01 | 0.23 | 0.45 |  | -0.01 | 0.31 | 0.62 |  | -0.02 | 0.24 | 0.58 |  | -0.02 | 0.21 | 0.57 |  | -0.02 | 0.25 | 0.58 |  | -0.01 | 0.32 | 0.63 |  |
| Turicibacter | -0.001 | 0.91 | 0.97 |  | 0.002 | 0.89 | 0.95 |  | -0.005 | 0.73 | 0.87 |  | -0.005 | 0.72 | 0.88 |  | -0.01 | 0.70 | 0.87 |  | -0.003 | 0.86 | 0.94 |  |
| Tuzzerella | 0.01 | 0.72 | 0.82 |  | 0.02 | 0.36 | 0.63 |  | 0.02 | 0.22 | 0.56 |  | 0.02 | 0.25 | 0.61 |  | 0.03 | 0.21 | 0.55 |  | 0.02 | 0.32 | 0.63 |  |
| UBA1819 | -0.01 | 0.58 | 0.72 |  | 0.002 | 0.86 | 0.94 |  | 0.02 | 0.07 | 0.31 |  | 0.02 | 0.07 | 0.32 |  | 0.02 | 0.09 | 0.33 |  | 0.02 | 0.13 | 0.48 |  |
| UCG.002 | -0.04 | 0.07 | 0.21 |  | -0.04 | 0.05 | 0.22 |  | 0.01 | 0.69 | 0.85 |  | 0.01 | 0.72 | 0.88 |  | 0.01 | 0.59 | 0.79 |  | 0.03 | 0.33 | 0.63 |  |
| UCG.003 | -0.05 | 0.01 | 0.09 |  | -0.06 | 0.001 | 0.04 |  | -0.06 | 0.01 | 0.12 |  | -0.06 | 0.01 | 0.17 |  | -0.06 | 0.01 | 0.17 |  | -0.05 | 0.05 | 0.34 |  |
| UCG.005 | -0.03 | 0.06 | 0.19 |  | -0.03 | 0.13 | 0.36 |  | 0.02 | 0.39 | 0.65 |  | 0.02 | 0.41 | 0.66 |  | 0.02 | 0.31 | 0.63 |  | 0.03 | 0.20 | 0.61 |  |
| UCG.009 | 0.0003 | 0.98 | 0.98 |  | 0.004 | 0.71 | 0.89 |  | 0.02 | 0.13 | 0.40 |  | 0.02 | 0.13 | 0.38 |  | 0.02 | 0.12 | 0.37 |  | 0.03 | 0.08 | 0.36 |  |
| Veillonella | -0.03 | 0.12 | 0.31 |  | -0.04 | 0.03 | 0.18 |  | -0.06 | 0.003 | 0.09 |  | -0.07 | 0.001 | 0.04 |  | -0.07 | 0.001 | 0.05 |  | -0.06 | 0.005 | 0.18 |  |
| Victivallis | -0.01 | 0.61 | 0.72 |  | -0.01 | 0.42 | 0.72 |  | 0.02 | 0.36 | 0.65 |  | 0.02 | 0.38 | 0.66 |  | 0.02 | 0.38 | 0.67 |  | 0.02 | 0.44 | 0.70 |  |
| Data source: The Coronary Artery Risk Development in Young Adults (CARDIA), 1985-2016  HOMA-Insulin resistance = Homeostatic Model Assessment for Insulin Resistance.  The false discovery rate (FDR) was adjusted using Benjamini-Hochberg method.  Model 1adjusted for sequencing run. Model 2 additionally adjusted for age, sex, race, field center, and education. Model 3 additionally adjusted for physical activity, smoking status, alcohol use, and diet quality score. In Model 4, medication use, such as proton pump inhibitor and lipid-lowering, was added. Model 5 included all covariates in Model 4 and added systolic blood pressure, diastolic blood pressure, and antihypertensive medication (Y/N). Model 6 included all covariates in Model 4 and further adjusted for diabetes medication use (Y/N). | | | | | | | | | | | | | | | | | | | | | | | | |

| Additional file 1: Table S2b. The genus-level association between diabetes duration and specific taxa (n = 605) | | | | | | | | | | | | | | | | | | | | | | | | |
| --- | --- | --- | --- | --- | --- | --- | --- | --- | --- | --- | --- | --- | --- | --- | --- | --- | --- | --- | --- | --- | --- | --- | --- | --- |
|  | Model 1 | | |  | Model 2 | | |  | Model 3 | | |  | Model 4 | | |  | Model 5 | | |  | Model 6 | | | |
|  | β | p | FDR |  | β | p | FDR |  | β | p | FDR |  | β | p | FDR |  | β | p | FDR |  | β | p | FDR |  |
| Acetanaerobacterium | -0.01 | 0.33 | 0.53 |  | -0.01 | 0.33 | 0.54 |  | -0.004 | 0.42 | 0.66 |  | -0.004 | 0.40 | 0.63 |  | -0.005 | 0.40 | 0.62 |  | -0.003 | 0.66 | 0.84 |  |
| Acidaminococcus | 0.01 | 0.58 | 0.73 |  | 0.004 | 0.78 | 0.89 |  | -0.003 | 0.85 | 0.93 |  | -0.01 | 0.75 | 0.86 |  | -0.01 | 0.61 | 0.73 |  | -0.01 | 0.58 | 0.81 |  |
| Actinomyces | 0.002 | 0.71 | 0.81 |  | -0.001 | 0.89 | 0.95 |  | -0.002 | 0.77 | 0.88 |  | -0.01 | 0.40 | 0.63 |  | -0.01 | 0.37 | 0.62 |  | -0.01 | 0.34 | 0.62 |  |
| Adlercreutzia | 0.005 | 0.63 | 0.76 |  | 0.01 | 0.24 | 0.44 |  | 0.02 | 0.13 | 0.33 |  | 0.01 | 0.17 | 0.41 |  | 0.02 | 0.09 | 0.27 |  | 0.02 | 0.09 | 0.37 |  |
| Agathobacter | -0.02 | 0.04 | 0.14 |  | -0.02 | 0.03 | 0.11 |  | -0.02 | 0.01 | 0.12 |  | -0.02 | 0.01 | 0.10 |  | -0.02 | 0.01 | 0.15 |  | -0.01 | 0.59 | 0.81 |  |
| Akkermansia | -0.03 | 0.10 | 0.23 |  | -0.02 | 0.27 | 0.47 |  | -0.02 | 0.30 | 0.56 |  | -0.01 | 0.41 | 0.63 |  | -0.01 | 0.50 | 0.69 |  | -0.02 | 0.31 | 0.60 |  |
| Alistipes | -0.01 | 0.12 | 0.25 |  | -0.01 | 0.21 | 0.41 |  | -0.01 | 0.31 | 0.56 |  | -0.01 | 0.25 | 0.51 |  | -0.01 | 0.29 | 0.57 |  | -0.01 | 0.21 | 0.51 |  |
| Allisonella | 0.01 | 0.33 | 0.53 |  | 0.003 | 0.70 | 0.82 |  | 0.002 | 0.84 | 0.92 |  | 0.001 | 0.93 | 0.97 |  | 0.0003 | 0.97 | 0.99 |  | -0.004 | 0.67 | 0.84 |  |
| Anaerofilum | -0.01 | 0.20 | 0.36 |  | -0.01 | 0.19 | 0.39 |  | -0.01 | 0.32 | 0.57 |  | -0.01 | 0.32 | 0.57 |  | -0.01 | 0.21 | 0.48 |  | -0.005 | 0.60 | 0.81 |  |
| Anaerostipes | -0.01 | 0.23 | 0.40 |  | -0.01 | 0.30 | 0.50 |  | -0.01 | 0.35 | 0.59 |  | -0.01 | 0.34 | 0.58 |  | -0.01 | 0.43 | 0.64 |  | 0.005 | 0.57 | 0.81 |  |
| Anaerotruncus | -0.01 | 0.12 | 0.25 |  | -0.01 | 0.12 | 0.28 |  | -0.01 | 0.26 | 0.51 |  | -0.01 | 0.21 | 0.45 |  | -0.01 | 0.18 | 0.42 |  | -0.02 | 0.02 | 0.20 |  |
| Bacteroides | 0.001 | 0.81 | 0.87 |  | 0.004 | 0.39 | 0.60 |  | 0.003 | 0.54 | 0.72 |  | 0.003 | 0.48 | 0.69 |  | 0.003 | 0.56 | 0.73 |  | 0.001 | 0.85 | 0.96 |  |
| Barnesiella | -0.04 | 0.01 | 0.06 |  | -0.04 | 0.01 | 0.07 |  | -0.04 | 0.02 | 0.12 |  | -0.04 | 0.02 | 0.10 |  | -0.04 | 0.02 | 0.15 |  | -0.03 | 0.22 | 0.51 |  |
| Bifidobacterium | -0.005 | 0.70 | 0.81 |  | -0.01 | 0.45 | 0.62 |  | -0.01 | 0.36 | 0.60 |  | -0.01 | 0.28 | 0.51 |  | -0.01 | 0.34 | 0.62 |  | 0.001 | 0.96 | 0.98 |  |
| Bilophila | 0.01 | 0.43 | 0.62 |  | 0.01 | 0.43 | 0.62 |  | 0.01 | 0.44 | 0.67 |  | 0.01 | 0.47 | 0.69 |  | 0.01 | 0.61 | 0.73 |  | 0.02 | 0.32 | 0.61 |  |
| Blautia | -0.002 | 0.56 | 0.71 |  | -0.0004 | 0.91 | 0.95 |  | -0.0001 | 0.98 | 0.98 |  | -0.001 | 0.87 | 0.95 |  | -0.002 | 0.61 | 0.73 |  | -0.002 | 0.67 | 0.84 |  |
| Butyricicoccus | -0.01 | 0.01 | 0.06 |  | -0.01 | 0.04 | 0.14 |  | -0.01 | 0.05 | 0.18 |  | -0.01 | 0.07 | 0.23 |  | -0.01 | 0.08 | 0.26 |  | -0.01 | 0.17 | 0.45 |  |
| Butyricimonas | 0.01 | 0.40 | 0.59 |  | 0.01 | 0.60 | 0.77 |  | 0.01 | 0.57 | 0.74 |  | 0.01 | 0.68 | 0.81 |  | 0.01 | 0.69 | 0.78 |  | -0.003 | 0.88 | 0.96 |  |
| CAG.56 | -0.01 | 0.23 | 0.40 |  | -0.01 | 0.29 | 0.49 |  | -0.01 | 0.33 | 0.57 |  | -0.01 | 0.27 | 0.51 |  | -0.02 | 0.23 | 0.50 |  | -0.02 | 0.28 | 0.59 |  |
| Candidatus_Soleaferrea | -0.02 | 0.002 | 0.03 |  | -0.02 | 0.002 | 0.03 |  | -0.02 | 0.002 | 0.06 |  | -0.02 | 0.002 | 0.07 |  | -0.02 | 0.003 | 0.08 |  | -0.02 | 0.003 | 0.10 |  |
| Caproiciproducens | -0.02 | 0.01 | 0.06 |  | -0.02 | 0.02 | 0.09 |  | -0.02 | 0.03 | 0.15 |  | -0.02 | 0.03 | 0.13 |  | -0.02 | 0.04 | 0.20 |  | -0.02 | 0.04 | 0.31 |  |
| Christensenellaceae_R.7_group | -0.04 | 0.01 | 0.06 |  | -0.04 | 0.005 | 0.05 |  | -0.03 | 0.02 | 0.12 |  | -0.03 | 0.02 | 0.10 |  | -0.03 | 0.04 | 0.20 |  | -0.03 | 0.09 | 0.37 |  |
| Clostridium_sensu_stricto_1 | -0.01 | 0.30 | 0.49 |  | -0.01 | 0.43 | 0.62 |  | -0.01 | 0.47 | 0.69 |  | -0.01 | 0.56 | 0.74 |  | -0.01 | 0.38 | 0.62 |  | 0.02 | 0.14 | 0.45 |  |
| Colidextribacter | -0.02 | 0.01 | 0.05 |  | -0.02 | 0.01 | 0.05 |  | -0.02 | 0.01 | 0.12 |  | -0.02 | 0.01 | 0.09 |  | -0.02 | 0.004 | 0.08 |  | -0.02 | 0.004 | 0.11 |  |
| Collinsella | -0.01 | 0.47 | 0.64 |  | -0.01 | 0.39 | 0.60 |  | -0.01 | 0.26 | 0.51 |  | -0.02 | 0.23 | 0.48 |  | -0.01 | 0.29 | 0.57 |  | -0.01 | 0.57 | 0.81 |  |
| Coprobacter | -0.02 | 0.07 | 0.18 |  | -0.02 | 0.11 | 0.27 |  | -0.02 | 0.19 | 0.42 |  | -0.01 | 0.25 | 0.51 |  | -0.01 | 0.36 | 0.62 |  | -0.01 | 0.37 | 0.64 |  |
| Coprococcus | -0.02 | 0.04 | 0.13 |  | -0.03 | 0.01 | 0.07 |  | -0.03 | 0.02 | 0.12 |  | -0.03 | 0.02 | 0.10 |  | -0.03 | 0.01 | 0.13 |  | -0.03 | 0.07 | 0.37 |  |
| Defluviitaleaceae_UCG.011 | -0.01 | 0.05 | 0.14 |  | -0.01 | 0.09 | 0.24 |  | -0.01 | 0.21 | 0.44 |  | -0.01 | 0.20 | 0.45 |  | -0.01 | 0.36 | 0.62 |  | -0.02 | 0.06 | 0.37 |  |
| Desulfovibrio | 0.01 | 0.47 | 0.64 |  | 0.003 | 0.82 | 0.92 |  | 0.01 | 0.52 | 0.70 |  | 0.01 | 0.63 | 0.80 |  | 0.01 | 0.41 | 0.62 |  | -0.01 | 0.46 | 0.76 |  |
| Dialister | -0.02 | 0.33 | 0.53 |  | -0.01 | 0.41 | 0.61 |  | -0.02 | 0.37 | 0.60 |  | -0.02 | 0.36 | 0.60 |  | -0.02 | 0.36 | 0.62 |  | -0.01 | 0.55 | 0.81 |  |
| DNF00809 | -0.02 | 0.005 | 0.05 |  | -0.02 | 0.01 | 0.05 |  | -0.02 | 0.02 | 0.12 |  | -0.02 | 0.01 | 0.10 |  | -0.02 | 0.03 | 0.15 |  | -0.02 | 0.01 | 0.18 |  |
| Dorea | 0.005 | 0.56 | 0.71 |  | 0.004 | 0.63 | 0.78 |  | 0.004 | 0.67 | 0.82 |  | 0.004 | 0.66 | 0.81 |  | 0.003 | 0.78 | 0.86 |  | 0.003 | 0.82 | 0.96 |  |
| DTU089 | -0.01 | 0.11 | 0.24 |  | -0.01 | 0.23 | 0.44 |  | -0.01 | 0.33 | 0.57 |  | -0.01 | 0.33 | 0.57 |  | -0.01 | 0.37 | 0.62 |  | -0.02 | 0.09 | 0.37 |  |
| Eggerthella | 0.001 | 0.88 | 0.89 |  | 0.003 | 0.71 | 0.82 |  | 0.004 | 0.62 | 0.80 |  | 0.005 | 0.59 | 0.77 |  | 0.001 | 0.87 | 0.92 |  | 0.002 | 0.83 | 0.96 |  |
| Eisenbergiella | -0.003 | 0.76 | 0.83 |  | -0.003 | 0.72 | 0.82 |  | 0.0001 | 0.99 | 0.99 |  | -0.003 | 0.74 | 0.86 |  | -0.005 | 0.57 | 0.73 |  | -0.01 | 0.54 | 0.81 |  |
| Erysipelatoclostridium | -0.01 | 0.19 | 0.36 |  | -0.01 | 0.27 | 0.47 |  | -0.01 | 0.36 | 0.60 |  | -0.01 | 0.44 | 0.66 |  | -0.01 | 0.41 | 0.62 |  | -0.01 | 0.48 | 0.78 |  |
| Erysipelotrichaceae_UCG.003 | -0.03 | 0.01 | 0.05 |  | -0.03 | 0.02 | 0.09 |  | -0.02 | 0.04 | 0.16 |  | -0.02 | 0.09 | 0.26 |  | -0.02 | 0.17 | 0.41 |  | -0.0003 | 0.98 | 0.99 |  |
| Escherichia.Shigella | 0.04 | <.001 | 0.01 |  | 0.04 | 0.001 | 0.03 |  | 0.04 | 0.002 | 0.06 |  | 0.03 | 0.01 | 0.09 |  | 0.03 | 0.02 | 0.15 |  | -0.01 | 0.65 | 0.84 |  |
| Faecalibacterium | -0.02 | 0.05 | 0.14 |  | -0.02 | 0.03 | 0.11 |  | -0.02 | 0.03 | 0.15 |  | -0.02 | 0.02 | 0.10 |  | -0.02 | 0.02 | 0.15 |  | 0.001 | 0.89 | 0.96 |  |
| Family_XIII_AD3011_group | -0.03 | 0.002 | 0.03 |  | -0.03 | 0.003 | 0.04 |  | -0.02 | 0.02 | 0.12 |  | -0.02 | 0.01 | 0.10 |  | -0.02 | 0.02 | 0.15 |  | -0.02 | 0.03 | 0.25 |  |
| Family_XIII_UCG.001 | -0.01 | 0.12 | 0.25 |  | -0.01 | 0.25 | 0.45 |  | -0.005 | 0.50 | 0.69 |  | -0.01 | 0.47 | 0.69 |  | -0.004 | 0.57 | 0.73 |  | -0.001 | 0.95 | 0.98 |  |
| FD2005 | -0.01 | 0.24 | 0.41 |  | -0.01 | 0.15 | 0.34 |  | -0.01 | 0.12 | 0.33 |  | -0.01 | 0.14 | 0.38 |  | -0.01 | 0.14 | 0.38 |  | -0.02 | 0.07 | 0.37 |  |
| Flavonifractor | -0.01 | 0.11 | 0.25 |  | -0.01 | 0.21 | 0.41 |  | -0.01 | 0.17 | 0.40 |  | -0.01 | 0.16 | 0.41 |  | -0.01 | 0.10 | 0.31 |  | -0.02 | 0.07 | 0.37 |  |
| Fournierella | -0.02 | 0.02 | 0.10 |  | -0.02 | 0.02 | 0.09 |  | -0.02 | 0.04 | 0.16 |  | -0.02 | 0.03 | 0.12 |  | -0.02 | 0.06 | 0.21 |  | -0.01 | 0.19 | 0.49 |  |
| Frisingicoccus | -0.01 | 0.28 | 0.46 |  | -0.01 | 0.49 | 0.66 |  | -0.004 | 0.70 | 0.84 |  | -0.01 | 0.49 | 0.69 |  | -0.01 | 0.40 | 0.62 |  | -0.02 | 0.09 | 0.37 |  |
| Fusicatenibacter | -0.01 | 0.20 | 0.36 |  | -0.01 | 0.20 | 0.40 |  | -0.02 | 0.15 | 0.37 |  | -0.01 | 0.21 | 0.45 |  | -0.01 | 0.27 | 0.56 |  | 0.005 | 0.73 | 0.90 |  |
| Fusobacterium | 0.02 | 0.01 | 0.06 |  | 0.02 | 0.01 | 0.07 |  | 0.02 | 0.01 | 0.12 |  | 0.02 | 0.01 | 0.10 |  | 0.02 | 0.02 | 0.15 |  | 0.01 | 0.13 | 0.45 |  |
| GCA.900066575 | -0.02 | 0.04 | 0.13 |  | -0.02 | 0.04 | 0.14 |  | -0.01 | 0.12 | 0.33 |  | -0.01 | 0.17 | 0.41 |  | -0.01 | 0.18 | 0.42 |  | -0.001 | 0.95 | 0.98 |  |
| Gordonibacter | -0.01 | 0.07 | 0.20 |  | -0.01 | 0.27 | 0.47 |  | -0.01 | 0.49 | 0.69 |  | -0.004 | 0.60 | 0.77 |  | -0.004 | 0.59 | 0.73 |  | -0.01 | 0.22 | 0.51 |  |
| Granulicatella | 0.01 | 0.09 | 0.22 |  | 0.01 | 0.18 | 0.38 |  | 0.01 | 0.18 | 0.42 |  | 0.01 | 0.33 | 0.57 |  | 0.01 | 0.38 | 0.62 |  | 0.01 | 0.12 | 0.45 |  |
| Haemophilus | 0.01 | 0.45 | 0.64 |  | 0.005 | 0.67 | 0.82 |  | 0.01 | 0.54 | 0.72 |  | 0.002 | 0.88 | 0.95 |  | 0.001 | 0.92 | 0.96 |  | 0.02 | 0.13 | 0.45 |  |
| Harryflintia | -0.01 | 0.14 | 0.27 |  | -0.01 | 0.15 | 0.34 |  | -0.01 | 0.19 | 0.42 |  | -0.01 | 0.17 | 0.41 |  | -0.01 | 0.16 | 0.41 |  | -0.02 | 0.02 | 0.23 |  |
| Holdemanella | 0.004 | 0.73 | 0.81 |  | -0.002 | 0.86 | 0.95 |  | -0.003 | 0.77 | 0.88 |  | -0.004 | 0.69 | 0.81 |  | -0.005 | 0.68 | 0.78 |  | -0.01 | 0.34 | 0.62 |  |
| Holdemania | -0.01 | 0.03 | 0.11 |  | -0.01 | 0.06 | 0.17 |  | -0.01 | 0.12 | 0.33 |  | -0.01 | 0.15 | 0.40 |  | -0.01 | 0.13 | 0.36 |  | -0.01 | 0.37 | 0.64 |  |
| Hungatella | 0.003 | 0.74 | 0.81 |  | 0.005 | 0.60 | 0.77 |  | 0.003 | 0.74 | 0.87 |  | 0.002 | 0.85 | 0.94 |  | -0.001 | 0.90 | 0.94 |  | -0.01 | 0.39 | 0.66 |  |
| Hydrogenoanaerobacterium | -0.02 | 0.01 | 0.06 |  | -0.02 | 0.02 | 0.09 |  | -0.02 | 0.05 | 0.18 |  | -0.02 | 0.05 | 0.21 |  | -0.02 | 0.05 | 0.20 |  | -0.02 | 0.06 | 0.37 |  |
| Incertae_Sedis | -0.004 | 0.47 | 0.64 |  | -0.002 | 0.68 | 0.82 |  | -0.002 | 0.77 | 0.88 |  | -0.002 | 0.66 | 0.81 |  | -0.003 | 0.59 | 0.73 |  | -0.01 | 0.31 | 0.60 |  |
| Intestinibacter | -0.02 | 0.04 | 0.13 |  | -0.01 | 0.10 | 0.26 |  | -0.02 | 0.05 | 0.18 |  | -0.02 | 0.05 | 0.21 |  | -0.02 | 0.06 | 0.21 |  | -0.01 | 0.42 | 0.71 |  |
| Intestinimonas | -0.02 | 0.04 | 0.13 |  | -0.02 | 0.04 | 0.14 |  | -0.02 | 0.11 | 0.31 |  | -0.01 | 0.13 | 0.38 |  | -0.01 | 0.15 | 0.38 |  | -0.03 | 0.01 | 0.15 |  |
| Lachnoclostridium | 0.01 | 0.05 | 0.14 |  | 0.01 | 0.05 | 0.15 |  | 0.01 | 0.13 | 0.33 |  | 0.01 | 0.15 | 0.40 |  | 0.01 | 0.18 | 0.42 |  | 0.003 | 0.57 | 0.81 |  |
| Lachnospira | -0.02 | 0.01 | 0.06 |  | -0.02 | 0.02 | 0.09 |  | -0.02 | 0.02 | 0.12 |  | -0.02 | 0.02 | 0.10 |  | -0.02 | 0.04 | 0.20 |  | -0.01 | 0.66 | 0.84 |  |
| Lachnospiraceae_FCS020_group | -0.01 | 0.13 | 0.27 |  | -0.01 | 0.11 | 0.27 |  | -0.01 | 0.18 | 0.42 |  | -0.01 | 0.18 | 0.43 |  | -0.01 | 0.23 | 0.50 |  | -0.01 | 0.60 | 0.81 |  |
| Lachnospiraceae_ND3007_group | -0.03 | 0.04 | 0.13 |  | -0.03 | 0.02 | 0.10 |  | -0.03 | 0.02 | 0.12 |  | -0.03 | 0.03 | 0.13 |  | -0.03 | 0.05 | 0.20 |  | -0.01 | 0.56 | 0.81 |  |
| Lachnospiraceae_NK4A136_group | -0.03 | 0.001 | 0.01 |  | -0.03 | <.001 | 0.03 |  | -0.03 | 0.001 | 0.06 |  | -0.03 | 0.002 | 0.07 |  | -0.03 | 0.003 | 0.08 |  | -0.02 | 0.05 | 0.37 |  |
| Lachnospiraceae_UCG.001 | -0.02 | 0.05 | 0.15 |  | -0.02 | 0.04 | 0.14 |  | -0.02 | 0.09 | 0.28 |  | -0.02 | 0.07 | 0.22 |  | -0.02 | 0.13 | 0.36 |  | -0.02 | 0.26 | 0.57 |  |
| Lachnospiraceae_UCG.004 | 0.01 | 0.48 | 0.64 |  | 0.01 | 0.50 | 0.67 |  | 0.01 | 0.49 | 0.69 |  | 0.01 | 0.50 | 0.69 |  | 0.01 | 0.52 | 0.71 |  | 0.02 | 0.18 | 0.48 |  |
| Lachnospiraceae_UCG.010 | -0.005 | 0.52 | 0.68 |  | -0.01 | 0.45 | 0.62 |  | -0.004 | 0.63 | 0.80 |  | -0.003 | 0.68 | 0.81 |  | -0.003 | 0.68 | 0.78 |  | 0.0001 | 0.99 | 0.99 |  |
| Lactobacillus | -0.002 | 0.82 | 0.87 |  | -0.01 | 0.48 | 0.66 |  | -0.01 | 0.23 | 0.47 |  | -0.02 | 0.07 | 0.23 |  | -0.02 | 0.05 | 0.20 |  | -0.02 | 0.16 | 0.45 |  |
| Lactococcus | -0.001 | 0.84 | 0.87 |  | 0.001 | 0.92 | 0.95 |  | 0.002 | 0.83 | 0.92 |  | -0.0003 | 0.96 | 0.98 |  | -0.001 | 0.94 | 0.96 |  | 0.002 | 0.80 | 0.96 |  |
| Marvinbryantia | -0.03 | 0.01 | 0.06 |  | -0.03 | 0.01 | 0.05 |  | -0.03 | 0.01 | 0.12 |  | -0.03 | 0.01 | 0.10 |  | -0.03 | 0.01 | 0.13 |  | -0.03 | 0.09 | 0.37 |  |
| Megasphaera | 0.03 | 0.02 | 0.08 |  | 0.03 | 0.05 | 0.16 |  | 0.02 | 0.09 | 0.28 |  | 0.02 | 0.14 | 0.38 |  | 0.02 | 0.24 | 0.52 |  | 0.02 | 0.31 | 0.60 |  |
| Monoglobus | -0.01 | 0.11 | 0.25 |  | -0.01 | 0.36 | 0.58 |  | -0.01 | 0.50 | 0.69 |  | -0.01 | 0.54 | 0.72 |  | -0.005 | 0.61 | 0.73 |  | -0.001 | 0.94 | 0.98 |  |
| Moryella | -0.01 | 0.16 | 0.30 |  | -0.01 | 0.12 | 0.30 |  | -0.01 | 0.19 | 0.42 |  | -0.01 | 0.27 | 0.51 |  | -0.01 | 0.28 | 0.57 |  | -0.01 | 0.34 | 0.62 |  |
| Negativibacillus | 0.002 | 0.84 | 0.87 |  | 0.004 | 0.71 | 0.82 |  | 0.004 | 0.72 | 0.85 |  | -0.001 | 0.93 | 0.97 |  | -0.002 | 0.85 | 0.91 |  | 0.001 | 0.95 | 0.98 |  |
| NK4A214_group | -0.02 | 0.08 | 0.20 |  | -0.03 | 0.04 | 0.14 |  | -0.02 | 0.10 | 0.31 |  | -0.03 | 0.06 | 0.22 |  | -0.03 | 0.06 | 0.21 |  | -0.02 | 0.27 | 0.58 |  |
| Odoribacter | -0.01 | 0.72 | 0.81 |  | -0.01 | 0.63 | 0.78 |  | -0.002 | 0.89 | 0.96 |  | -0.001 | 0.94 | 0.97 |  | -0.004 | 0.80 | 0.88 |  | -0.02 | 0.26 | 0.57 |  |
| Oscillibacter | -0.03 | <.001 | 0.01 |  | -0.03 | 0.001 | 0.03 |  | -0.02 | 0.003 | 0.07 |  | -0.02 | 0.003 | 0.07 |  | -0.03 | 0.002 | 0.08 |  | -0.03 | 0.003 | 0.10 |  |
| Oscillospira | -0.02 | 0.01 | 0.06 |  | -0.01 | 0.05 | 0.16 |  | -0.01 | 0.06 | 0.21 |  | -0.01 | 0.06 | 0.21 |  | -0.01 | 0.06 | 0.21 |  | -0.02 | 0.03 | 0.25 |  |
| Oxalobacter | 0.000 | 0.96 | 0.96 |  | -0.001 | 0.89 | 0.95 |  | 0.001 | 0.90 | 0.96 |  | 0.0002 | 0.98 | 0.99 |  | 0.01 | 0.49 | 0.69 |  | -0.02 | 0.11 | 0.42 |  |
| Paludicola | -0.03 | 0.001 | 0.02 |  | -0.02 | 0.003 | 0.04 |  | -0.02 | 0.02 | 0.12 |  | -0.02 | 0.01 | 0.10 |  | -0.02 | 0.02 | 0.15 |  | -0.03 | 0.001 | 0.10 |  |
| Parabacteroides | -0.001 | 0.96 | 0.96 |  | -0.001 | 0.92 | 0.95 |  | -0.003 | 0.79 | 0.89 |  | -0.005 | 0.67 | 0.81 |  | -0.005 | 0.69 | 0.78 |  | 0.001 | 0.94 | 0.98 |  |
| Paraprevotella | 0.03 | 0.08 | 0.21 |  | 0.02 | 0.18 | 0.38 |  | 0.02 | 0.16 | 0.38 |  | 0.02 | 0.21 | 0.45 |  | 0.02 | 0.31 | 0.58 |  | 0.04 | 0.06 | 0.37 |  |
| Parasutterella | 0.01 | 0.59 | 0.74 |  | 0.01 | 0.41 | 0.61 |  | 0.01 | 0.39 | 0.62 |  | 0.02 | 0.28 | 0.51 |  | 0.02 | 0.11 | 0.33 |  | 0.03 | 0.15 | 0.45 |  |
| Peptococcus | -0.02 | 0.01 | 0.06 |  | -0.02 | 0.03 | 0.12 |  | -0.01 | 0.06 | 0.21 |  | -0.01 | 0.06 | 0.22 |  | -0.01 | 0.07 | 0.25 |  | -0.01 | 0.15 | 0.45 |  |
| Phascolarctobacterium | 0.003 | 0.83 | 0.87 |  | 0.002 | 0.88 | 0.95 |  | 0.01 | 0.70 | 0.84 |  | 0.01 | 0.76 | 0.86 |  | 0.01 | 0.57 | 0.73 |  | 0.01 | 0.70 | 0.87 |  |
| Phocea | 0.002 | 0.69 | 0.81 |  | 0.004 | 0.55 | 0.73 |  | 0.003 | 0.67 | 0.82 |  | 0.003 | 0.64 | 0.80 |  | 0.002 | 0.75 | 0.84 |  | 0.01 | 0.22 | 0.51 |  |
| Prevotella | 0.02 | 0.22 | 0.39 |  | 0.01 | 0.44 | 0.62 |  | 0.01 | 0.46 | 0.68 |  | 0.01 | 0.53 | 0.71 |  | 0.01 | 0.41 | 0.62 |  | 0.01 | 0.77 | 0.94 |  |
| Romboutsia | -0.04 | <.001 | 0.01 |  | -0.03 | 0.001 | 0.03 |  | -0.03 | 0.001 | 0.06 |  | -0.03 | 0.002 | 0.07 |  | -0.03 | 0.004 | 0.08 |  | -0.002 | 0.85 | 0.96 |  |
| Roseburia | -0.02 | 0.03 | 0.11 |  | -0.02 | 0.06 | 0.17 |  | -0.02 | 0.05 | 0.18 |  | -0.02 | 0.05 | 0.21 |  | -0.02 | 0.05 | 0.20 |  | -0.01 | 0.55 | 0.81 |  |
| Ruminococcus | -0.04 | 0.001 | 0.02 |  | -0.04 | 0.002 | 0.03 |  | -0.04 | 0.004 | 0.07 |  | -0.04 | 0.004 | 0.09 |  | -0.03 | 0.01 | 0.13 |  | -0.02 | 0.13 | 0.45 |  |
| Sellimonas | -0.02 | 0.06 | 0.16 |  | -0.02 | 0.03 | 0.12 |  | -0.02 | 0.09 | 0.28 |  | -0.02 | 0.08 | 0.25 |  | -0.02 | 0.08 | 0.26 |  | -0.02 | 0.09 | 0.37 |  |
| Senegalimassilia | -0.02 | 0.09 | 0.22 |  | -0.02 | 0.02 | 0.09 |  | -0.02 | 0.02 | 0.12 |  | -0.02 | 0.02 | 0.10 |  | -0.02 | 0.02 | 0.15 |  | -0.03 | 0.02 | 0.20 |  |
| Shuttleworthia | -0.005 | 0.62 | 0.76 |  | 0.0002 | 0.99 | 0.99 |  | 0.004 | 0.67 | 0.82 |  | 0.003 | 0.77 | 0.86 |  | 0.0001 | 0.99 | 0.99 |  | 0.01 | 0.37 | 0.64 |  |
| Slackia | 0.01 | 0.52 | 0.68 |  | -0.001 | 0.94 | 0.95 |  | -0.001 | 0.90 | 0.96 |  | -0.001 | 0.91 | 0.97 |  | -0.0002 | 0.99 | 0.99 |  | -0.002 | 0.87 | 0.96 |  |
| Streptococcus | 0.003 | 0.72 | 0.81 |  | -0.0001 | 0.99 | 0.99 |  | 0.001 | 0.93 | 0.96 |  | -0.01 | 0.40 | 0.63 |  | -0.01 | 0.35 | 0.62 |  | 0.01 | 0.57 | 0.81 |  |
| Subdoligranulum | -0.01 | 0.14 | 0.27 |  | -0.01 | 0.16 | 0.34 |  | -0.01 | 0.27 | 0.51 |  | -0.01 | 0.26 | 0.51 |  | -0.01 | 0.29 | 0.57 |  | -0.02 | 0.18 | 0.48 |  |
| Sutterella | -0.01 | 0.72 | 0.81 |  | -0.02 | 0.31 | 0.51 |  | -0.02 | 0.34 | 0.58 |  | -0.01 | 0.48 | 0.69 |  | -0.01 | 0.46 | 0.66 |  | 0.004 | 0.84 | 0.96 |  |
| TM7x | -0.01 | 0.39 | 0.59 |  | -0.01 | 0.37 | 0.59 |  | -0.01 | 0.46 | 0.68 |  | -0.01 | 0.41 | 0.63 |  | -0.005 | 0.55 | 0.73 |  | -0.002 | 0.86 | 0.96 |  |
| Turicibacter | -0.01 | 0.47 | 0.64 |  | -0.004 | 0.57 | 0.74 |  | -0.005 | 0.51 | 0.69 |  | -0.005 | 0.51 | 0.69 |  | -0.01 | 0.46 | 0.66 |  | -0.002 | 0.81 | 0.96 |  |
| Tuzzerella | -0.003 | 0.80 | 0.86 |  | 0.001 | 0.91 | 0.95 |  | 0.001 | 0.93 | 0.96 |  | -0.0001 | 0.99 | 0.99 |  | 0.002 | 0.83 | 0.89 |  | -0.01 | 0.65 | 0.84 |  |
| UBA1819 | -0.01 | 0.45 | 0.64 |  | -0.003 | 0.63 | 0.78 |  | -0.001 | 0.92 | 0.96 |  | -0.001 | 0.90 | 0.96 |  | -0.004 | 0.61 | 0.73 |  | -0.01 | 0.30 | 0.60 |  |
| UCG.002 | -0.04 | 0.01 | 0.06 |  | -0.04 | 0.005 | 0.05 |  | -0.03 | 0.02 | 0.12 |  | -0.03 | 0.02 | 0.10 |  | -0.03 | 0.03 | 0.16 |  | -0.02 | 0.20 | 0.49 |  |
| UCG.003 | -0.01 | 0.36 | 0.55 |  | -0.02 | 0.16 | 0.34 |  | -0.02 | 0.23 | 0.47 |  | -0.01 | 0.28 | 0.51 |  | -0.01 | 0.30 | 0.57 |  | 0.01 | 0.55 | 0.81 |  |
| UCG.005 | -0.03 | 0.01 | 0.05 |  | -0.03 | 0.01 | 0.05 |  | -0.03 | 0.02 | 0.12 |  | -0.03 | 0.02 | 0.10 |  | -0.02 | 0.04 | 0.20 |  | -0.03 | 0.07 | 0.37 |  |
| UCG.009 | -0.02 | 0.01 | 0.05 |  | -0.02 | 0.01 | 0.05 |  | -0.02 | 0.01 | 0.12 |  | -0.02 | 0.01 | 0.10 |  | -0.02 | 0.01 | 0.13 |  | -0.02 | 0.01 | 0.17 |  |
| Veillonella | 0.005 | 0.66 | 0.80 |  | 0.001 | 0.91 | 0.95 |  | 0.0004 | 0.97 | 0.98 |  | -0.003 | 0.77 | 0.86 |  | -0.005 | 0.66 | 0.77 |  | 0.02 | 0.14 | 0.45 |  |
| Victivallis | -0.01 | 0.40 | 0.59 |  | -0.01 | 0.23 | 0.44 |  | -0.01 | 0.43 | 0.67 |  | -0.01 | 0.40 | 0.63 |  | -0.01 | 0.44 | 0.65 |  | -0.02 | 0.16 | 0.45 |  |
| Data source: The Coronary Artery Risk Development in Young Adults (CARDIA), 1985-2016  The false discovery rate (FDR) was adjusted using Benjamini-Hochberg method.  Model 1adjusted for sequencing run. Model 2 additionally adjusted for age, sex, race, field center, and education. Model 3 additionally adjusted for physical activity, smoking status, alcohol use, and diet quality score. In Model 4, medication use, such as proton pump inhibitor and lipid-lowering, was added. Model 5 included all covariates in Model 4 and added systolic blood pressure, diastolic blood pressure, and antihypertensive medication (Y/N). Model 6 included all covariates in Model 4 and further adjusted for diabetes medication use (Y/N). | | | | | | | | | | | | | | | | | | | | | | | | |

| Additional file 1: Table S2c. The genus-level association between stages of diabetes (normal vs. prediabetes) and specific taxa (n = 605, reference = normal) | | | | | | | | | | | | | | | | | | | | | | | | |
| --- | --- | --- | --- | --- | --- | --- | --- | --- | --- | --- | --- | --- | --- | --- | --- | --- | --- | --- | --- | --- | --- | --- | --- | --- |
|  | Model 1 | | |  | Model 2 | | |  | Model 3 | | |  | Model 4 | | |  | Model 5 | | |  | Model 6 | | | |
|  | β | p | FDR |  | β | p | FDR |  | β | p | FDR |  | β | p | FDR |  | β | p | FDR |  | β | p | FDR |  |
| Acetanaerobacterium | -0.04 | 0.43 | 0.69 |  | -0.01 | 0.82 | 0.93 |  | -0.002 | 0.97 | 1.00 |  | -0.0004 | 0.99 | 1.00 |  | -0.003 | 0.96 | 0.98 |  | -0.0004 | 0.99 | 1.00 |  |
| Acidaminococcus | 0.04 | 0.80 | 0.91 |  | -0.06 | 0.72 | 0.88 |  | -0.16 | 0.32 | 0.78 |  | -0.18 | 0.26 | 0.73 |  | -0.19 | 0.25 | 0.77 |  | -0.18 | 0.27 | 0.82 |  |
| Actinomyces | -0.04 | 0.48 | 0.73 |  | -0.08 | 0.20 | 0.56 |  | -0.10 | 0.10 | 0.57 |  | -0.12 | 0.04 | 0.40 |  | -0.13 | 0.03 | 0.34 |  | -0.12 | 0.04 | 0.59 |  |
| Adlercreutzia | -0.02 | 0.87 | 0.93 |  | 0.06 | 0.52 | 0.79 |  | 0.09 | 0.36 | 0.80 |  | 0.10 | 0.33 | 0.78 |  | 0.12 | 0.26 | 0.78 |  | 0.10 | 0.34 | 0.84 |  |
| Agathobacter | 0.03 | 0.67 | 0.85 |  | -0.01 | 0.89 | 0.95 |  | -0.04 | 0.59 | 0.85 |  | -0.03 | 0.66 | 0.89 |  | -0.04 | 0.60 | 0.88 |  | -0.03 | 0.67 | 0.97 |  |
| Akkermansia | -0.30 | 0.05 | 0.26 |  | -0.17 | 0.27 | 0.60 |  | -0.13 | 0.40 | 0.83 |  | -0.13 | 0.42 | 0.83 |  | -0.09 | 0.57 | 0.87 |  | -0.13 | 0.43 | 0.89 |  |
| Alistipes | -0.03 | 0.78 | 0.91 |  | 0.01 | 0.91 | 0.96 |  | 0.05 | 0.61 | 0.85 |  | 0.04 | 0.69 | 0.90 |  | 0.04 | 0.66 | 0.89 |  | 0.04 | 0.67 | 0.97 |  |
| Allisonella | 0.01 | 0.87 | 0.93 |  | -0.08 | 0.28 | 0.62 |  | -0.12 | 0.14 | 0.68 |  | -0.12 | 0.15 | 0.65 |  | -0.12 | 0.13 | 0.67 |  | -0.11 | 0.15 | 0.75 |  |
| Anaerofilum | -0.13 | 0.06 | 0.27 |  | -0.10 | 0.15 | 0.49 |  | -0.09 | 0.22 | 0.73 |  | -0.08 | 0.24 | 0.71 |  | -0.08 | 0.24 | 0.77 |  | -0.08 | 0.24 | 0.79 |  |
| Anaerostipes | -0.08 | 0.25 | 0.53 |  | -0.06 | 0.39 | 0.70 |  | -0.05 | 0.49 | 0.84 |  | -0.04 | 0.51 | 0.86 |  | -0.03 | 0.65 | 0.89 |  | -0.05 | 0.50 | 0.89 |  |
| Anaerotruncus | -0.20 | 0.01 | 0.09 |  | -0.14 | 0.06 | 0.35 |  | -0.10 | 0.20 | 0.73 |  | -0.10 | 0.22 | 0.71 |  | -0.08 | 0.28 | 0.79 |  | -0.09 | 0.23 | 0.78 |  |
| Bacteroides | 0.004 | 0.94 | 0.96 |  | 0.04 | 0.34 | 0.65 |  | 0.02 | 0.73 | 0.89 |  | 0.01 | 0.77 | 0.92 |  | 0.01 | 0.85 | 0.94 |  | 0.01 | 0.77 | 0.97 |  |
| Barnesiella | -0.21 | 0.20 | 0.48 |  | -0.21 | 0.21 | 0.56 |  | -0.20 | 0.23 | 0.73 |  | -0.22 | 0.20 | 0.70 |  | -0.21 | 0.23 | 0.77 |  | -0.21 | 0.21 | 0.77 |  |
| Bifidobacterium | 0.10 | 0.44 | 0.70 |  | 0.09 | 0.49 | 0.77 |  | 0.06 | 0.62 | 0.85 |  | 0.04 | 0.76 | 0.92 |  | 0.03 | 0.81 | 0.93 |  | 0.04 | 0.75 | 0.97 |  |
| Bilophila | -0.09 | 0.46 | 0.71 |  | -0.09 | 0.45 | 0.73 |  | -0.09 | 0.46 | 0.84 |  | -0.10 | 0.42 | 0.83 |  | -0.10 | 0.42 | 0.83 |  | -0.10 | 0.41 | 0.88 |  |
| Blautia | 0.01 | 0.74 | 0.89 |  | 0.03 | 0.40 | 0.70 |  | 0.02 | 0.49 | 0.84 |  | 0.02 | 0.50 | 0.85 |  | 0.03 | 0.37 | 0.83 |  | 0.02 | 0.50 | 0.89 |  |
| Butyricicoccus | -0.04 | 0.46 | 0.71 |  | -0.01 | 0.81 | 0.93 |  | -0.02 | 0.73 | 0.89 |  | -0.02 | 0.73 | 0.91 |  | -0.01 | 0.85 | 0.94 |  | -0.02 | 0.72 | 0.97 |  |
| Butyricimonas | 0.20 | 0.15 | 0.42 |  | 0.13 | 0.36 | 0.67 |  | 0.11 | 0.44 | 0.84 |  | 0.10 | 0.50 | 0.85 |  | 0.13 | 0.38 | 0.83 |  | 0.10 | 0.50 | 0.89 |  |
| CAG.56 | -0.08 | 0.51 | 0.75 |  | -0.07 | 0.56 | 0.82 |  | -0.06 | 0.65 | 0.87 |  | -0.07 | 0.59 | 0.87 |  | -0.07 | 0.57 | 0.87 |  | -0.07 | 0.60 | 0.94 |  |
| Candidatus_Soleaferrea | 0.01 | 0.89 | 0.94 |  | 0.05 | 0.45 | 0.74 |  | 0.04 | 0.50 | 0.84 |  | 0.04 | 0.48 | 0.85 |  | 0.05 | 0.44 | 0.83 |  | 0.05 | 0.45 | 0.89 |  |
| Caproiciproducens | -0.02 | 0.78 | 0.91 |  | 0.01 | 0.88 | 0.95 |  | 0.01 | 0.87 | 0.96 |  | 0.02 | 0.82 | 0.93 |  | 0.03 | 0.70 | 0.91 |  | 0.02 | 0.80 | 0.97 |  |
| Christensenellaceae_R.7_group | -0.26 | 0.06 | 0.28 |  | -0.26 | 0.07 | 0.38 |  | -0.15 | 0.29 | 0.77 |  | -0.15 | 0.31 | 0.77 |  | -0.12 | 0.43 | 0.83 |  | -0.14 | 0.33 | 0.84 |  |
| Clostridium_sensu_stricto_1 | 0.24 | 0.03 | 0.19 |  | 0.25 | 0.02 | 0.25 |  | 0.28 | 0.02 | 0.26 |  | 0.27 | 0.02 | 0.25 |  | 0.29 | 0.01 | 0.22 |  | 0.27 | 0.02 | 0.44 |  |
| Colidextribacter | -0.04 | 0.49 | 0.74 |  | -0.02 | 0.74 | 0.89 |  | -0.02 | 0.69 | 0.88 |  | -0.03 | 0.67 | 0.89 |  | -0.04 | 0.56 | 0.87 |  | -0.02 | 0.69 | 0.97 |  |
| Collinsella | 0.18 | 0.17 | 0.44 |  | 0.12 | 0.36 | 0.67 |  | 0.07 | 0.61 | 0.85 |  | 0.04 | 0.76 | 0.92 |  | 0.03 | 0.81 | 0.93 |  | 0.04 | 0.75 | 0.97 |  |
| Coprobacter | -0.09 | 0.43 | 0.70 |  | -0.09 | 0.44 | 0.72 |  | -0.05 | 0.71 | 0.88 |  | -0.03 | 0.79 | 0.92 |  | -0.02 | 0.86 | 0.95 |  | -0.03 | 0.79 | 0.97 |  |
| Coprococcus | -0.05 | 0.62 | 0.81 |  | -0.11 | 0.33 | 0.65 |  | -0.12 | 0.28 | 0.77 |  | -0.12 | 0.28 | 0.75 |  | -0.11 | 0.33 | 0.82 |  | -0.12 | 0.30 | 0.84 |  |
| Defluviitaleaceae_UCG.011 | -0.11 | 0.14 | 0.41 |  | -0.04 | 0.62 | 0.85 |  | 0.01 | 0.86 | 0.96 |  | 0.02 | 0.77 | 0.92 |  | 0.04 | 0.60 | 0.88 |  | 0.03 | 0.74 | 0.97 |  |
| Desulfovibrio | -0.13 | 0.32 | 0.60 |  | -0.24 | 0.07 | 0.38 |  | -0.16 | 0.24 | 0.73 |  | -0.17 | 0.22 | 0.71 |  | -0.14 | 0.30 | 0.79 |  | -0.16 | 0.22 | 0.77 |  |
| Dialister | -0.04 | 0.79 | 0.91 |  | -0.01 | 0.98 | 0.99 |  | -0.03 | 0.88 | 0.96 |  | -0.01 | 0.97 | 1.00 |  | -0.07 | 0.69 | 0.90 |  | -0.01 | 0.97 | 1.00 |  |
| DNF00809 | -0.25 | <.001 | 0.01 |  | -0.21 | 0.002 | 0.07 |  | -0.18 | 0.01 | 0.19 |  | -0.18 | 0.01 | 0.18 |  | -0.18 | 0.01 | 0.20 |  | -0.18 | 0.01 | 0.32 |  |
| Dorea | -0.11 | 0.21 | 0.48 |  | -0.15 | 0.08 | 0.39 |  | -0.17 | 0.05 | 0.45 |  | -0.17 | 0.06 | 0.44 |  | -0.17 | 0.06 | 0.44 |  | -0.17 | 0.06 | 0.63 |  |
| DTU089 | -0.09 | 0.25 | 0.53 |  | -0.04 | 0.63 | 0.85 |  | -0.02 | 0.77 | 0.91 |  | -0.02 | 0.80 | 0.92 |  | -0.03 | 0.77 | 0.93 |  | -0.02 | 0.81 | 0.97 |  |
| Eggerthella | -0.09 | 0.29 | 0.58 |  | -0.02 | 0.83 | 0.93 |  | 0.01 | 0.89 | 0.97 |  | 0.01 | 0.87 | 0.96 |  | 0.01 | 0.93 | 0.97 |  | 0.01 | 0.89 | 0.97 |  |
| Eisenbergiella | -0.12 | 0.16 | 0.44 |  | -0.05 | 0.52 | 0.79 |  | 0.02 | 0.84 | 0.95 |  | 0.01 | 0.92 | 0.98 |  | 0.01 | 0.88 | 0.95 |  | 0.01 | 0.91 | 0.97 |  |
| Erysipelatoclostridium | 0.09 | 0.27 | 0.55 |  | 0.14 | 0.08 | 0.39 |  | 0.15 | 0.07 | 0.49 |  | 0.16 | 0.05 | 0.42 |  | 0.15 | 0.06 | 0.44 |  | 0.16 | 0.05 | 0.59 |  |
| Erysipelotrichaceae_UCG.003 | 0.01 | 0.92 | 0.95 |  | 0.07 | 0.54 | 0.80 |  | 0.10 | 0.38 | 0.82 |  | 0.11 | 0.31 | 0.77 |  | 0.12 | 0.28 | 0.79 |  | 0.11 | 0.32 | 0.84 |  |
| Escherichia.Shigella | 0.10 | 0.37 | 0.65 |  | 0.12 | 0.30 | 0.63 |  | 0.09 | 0.46 | 0.84 |  | 0.08 | 0.53 | 0.86 |  | 0.06 | 0.62 | 0.88 |  | 0.08 | 0.52 | 0.90 |  |
| Faecalibacterium | 0.06 | 0.46 | 0.71 |  | 0.03 | 0.75 | 0.89 |  | 0.05 | 0.55 | 0.85 |  | 0.05 | 0.55 | 0.86 |  | 0.06 | 0.46 | 0.84 |  | 0.05 | 0.55 | 0.90 |  |
| Family_XIII_AD3011_group | -0.17 | 0.05 | 0.26 |  | -0.16 | 0.07 | 0.38 |  | -0.09 | 0.31 | 0.78 |  | -0.09 | 0.33 | 0.78 |  | -0.06 | 0.47 | 0.84 |  | -0.08 | 0.35 | 0.84 |  |
| Family_XIII_UCG.001 | -0.18 | 0.01 | 0.13 |  | -0.17 | 0.02 | 0.20 |  | -0.13 | 0.08 | 0.50 |  | -0.13 | 0.08 | 0.52 |  | -0.10 | 0.16 | 0.70 |  | -0.13 | 0.08 | 0.69 |  |
| FD2005 | 0.05 | 0.51 | 0.75 |  | 0.002 | 0.98 | 0.99 |  | -0.02 | 0.84 | 0.95 |  | 0.001 | 0.99 | 1.00 |  | -0.01 | 0.91 | 0.97 |  | 0.004 | 0.96 | 0.99 |  |
| Flavonifractor | 0.03 | 0.72 | 0.87 |  | 0.10 | 0.23 | 0.56 |  | 0.08 | 0.39 | 0.82 |  | 0.08 | 0.39 | 0.83 |  | 0.05 | 0.60 | 0.88 |  | 0.08 | 0.39 | 0.86 |  |
| Fournierella | -0.05 | 0.54 | 0.77 |  | -0.09 | 0.26 | 0.60 |  | -0.07 | 0.36 | 0.80 |  | -0.08 | 0.34 | 0.80 |  | -0.07 | 0.42 | 0.83 |  | -0.07 | 0.36 | 0.84 |  |
| Frisingicoccus | 0.05 | 0.60 | 0.80 |  | 0.13 | 0.20 | 0.56 |  | 0.20 | 0.06 | 0.49 |  | 0.20 | 0.05 | 0.42 |  | 0.23 | 0.03 | 0.35 |  | 0.21 | 0.05 | 0.59 |  |
| Fusicatenibacter | -0.17 | 0.12 | 0.38 |  | -0.19 | 0.07 | 0.38 |  | -0.22 | 0.04 | 0.43 |  | -0.21 | 0.06 | 0.44 |  | -0.21 | 0.06 | 0.44 |  | -0.21 | 0.06 | 0.63 |  |
| Fusobacterium | 0.12 | 0.10 | 0.34 |  | 0.11 | 0.15 | 0.50 |  | 0.10 | 0.18 | 0.73 |  | 0.10 | 0.19 | 0.70 |  | 0.09 | 0.25 | 0.77 |  | 0.10 | 0.20 | 0.77 |  |
| GCA.900066575 | 0.02 | 0.81 | 0.91 |  | 0.04 | 0.60 | 0.84 |  | 0.10 | 0.21 | 0.73 |  | 0.11 | 0.17 | 0.69 |  | 0.11 | 0.17 | 0.72 |  | 0.11 | 0.17 | 0.77 |  |
| Gordonibacter | -0.11 | 0.19 | 0.46 |  | -0.01 | 0.93 | 0.96 |  | 0.05 | 0.53 | 0.85 |  | 0.06 | 0.42 | 0.83 |  | 0.08 | 0.34 | 0.82 |  | 0.07 | 0.41 | 0.88 |  |
| Granulicatella | 0.11 | 0.08 | 0.32 |  | 0.10 | 0.09 | 0.39 |  | 0.10 | 0.12 | 0.62 |  | 0.08 | 0.20 | 0.70 |  | 0.07 | 0.22 | 0.77 |  | 0.08 | 0.21 | 0.77 |  |
| Haemophilus | -0.05 | 0.61 | 0.81 |  | -0.09 | 0.42 | 0.72 |  | -0.04 | 0.72 | 0.89 |  | -0.08 | 0.48 | 0.85 |  | -0.06 | 0.57 | 0.87 |  | -0.08 | 0.47 | 0.89 |  |
| Harryflintia | -0.002 | 0.98 | 0.98 |  | 0.02 | 0.81 | 0.93 |  | 0.03 | 0.69 | 0.88 |  | 0.03 | 0.65 | 0.89 |  | 0.03 | 0.66 | 0.89 |  | 0.04 | 0.62 | 0.96 |  |
| Holdemanella | -0.02 | 0.82 | 0.92 |  | -0.13 | 0.22 | 0.56 |  | -0.17 | 0.13 | 0.62 |  | -0.18 | 0.11 | 0.58 |  | -0.17 | 0.12 | 0.63 |  | -0.17 | 0.12 | 0.75 |  |
| Holdemania | -0.02 | 0.58 | 0.79 |  | 0.01 | 0.91 | 0.96 |  | 0.02 | 0.63 | 0.85 |  | 0.02 | 0.59 | 0.87 |  | 0.03 | 0.58 | 0.87 |  | 0.03 | 0.59 | 0.93 |  |
| Hungatella | -0.05 | 0.59 | 0.79 |  | 0.02 | 0.80 | 0.93 |  | 0.01 | 0.89 | 0.97 |  | 0.0003 | 1.00 | 1.00 |  | -0.002 | 0.98 | 0.99 |  | -0.0003 | 1.00 | 1.00 |  |
| Hydrogenoanaerobacterium | -0.18 | 0.02 | 0.16 |  | -0.14 | 0.08 | 0.39 |  | -0.08 | 0.30 | 0.78 |  | -0.09 | 0.25 | 0.71 |  | -0.07 | 0.38 | 0.83 |  | -0.09 | 0.25 | 0.81 |  |
| Incertae_Sedis | 0.01 | 0.79 | 0.91 |  | 0.04 | 0.50 | 0.77 |  | 0.05 | 0.35 | 0.80 |  | 0.05 | 0.36 | 0.81 |  | 0.04 | 0.43 | 0.83 |  | 0.05 | 0.36 | 0.84 |  |
| Intestinibacter | 0.07 | 0.45 | 0.71 |  | 0.09 | 0.34 | 0.65 |  | 0.03 | 0.75 | 0.91 |  | 0.03 | 0.76 | 0.92 |  | 0.03 | 0.76 | 0.93 |  | 0.03 | 0.75 | 0.97 |  |
| Intestinimonas | -0.13 | 0.17 | 0.44 |  | -0.05 | 0.61 | 0.84 |  | 0.03 | 0.75 | 0.90 |  | 0.03 | 0.74 | 0.92 |  | 0.04 | 0.70 | 0.91 |  | 0.04 | 0.72 | 0.97 |  |
| Lachnoclostridium | 0.08 | 0.07 | 0.28 |  | 0.08 | 0.09 | 0.39 |  | 0.04 | 0.40 | 0.83 |  | 0.04 | 0.42 | 0.83 |  | 0.02 | 0.60 | 0.88 |  | 0.04 | 0.43 | 0.89 |  |
| Lachnospira | 0.06 | 0.54 | 0.77 |  | 0.06 | 0.48 | 0.76 |  | 0.07 | 0.45 | 0.84 |  | 0.07 | 0.43 | 0.83 |  | 0.09 | 0.34 | 0.82 |  | 0.08 | 0.41 | 0.88 |  |
| Lachnospiraceae_FCS020_group | -0.18 | 0.04 | 0.21 |  | -0.20 | 0.02 | 0.21 |  | -0.18 | 0.04 | 0.42 |  | -0.18 | 0.05 | 0.42 |  | -0.17 | 0.06 | 0.44 |  | -0.18 | 0.05 | 0.59 |  |
| Lachnospiraceae_ND3007_group | -0.12 | 0.34 | 0.61 |  | -0.16 | 0.21 | 0.56 |  | -0.19 | 0.16 | 0.72 |  | -0.17 | 0.20 | 0.70 |  | -0.18 | 0.20 | 0.77 |  | -0.17 | 0.20 | 0.77 |  |
| Lachnospiraceae_NK4A136_group | -0.10 | 0.24 | 0.52 |  | -0.10 | 0.27 | 0.60 |  | -0.05 | 0.56 | 0.85 |  | -0.05 | 0.60 | 0.87 |  | -0.04 | 0.68 | 0.90 |  | -0.04 | 0.62 | 0.96 |  |
| Lachnospiraceae_UCG.001 | -0.12 | 0.31 | 0.59 |  | -0.11 | 0.35 | 0.66 |  | -0.06 | 0.63 | 0.85 |  | -0.07 | 0.56 | 0.86 |  | -0.08 | 0.54 | 0.87 |  | -0.07 | 0.58 | 0.93 |  |
| Lachnospiraceae_UCG.004 | 0.06 | 0.57 | 0.79 |  | 0.02 | 0.83 | 0.93 |  | 0.05 | 0.69 | 0.88 |  | 0.04 | 0.74 | 0.91 |  | 0.01 | 0.93 | 0.97 |  | 0.04 | 0.75 | 0.97 |  |
| Lachnospiraceae_UCG.010 | -0.01 | 0.92 | 0.95 |  | -0.03 | 0.71 | 0.88 |  | -0.01 | 0.93 | 0.99 |  | -0.01 | 0.93 | 0.98 |  | -0.02 | 0.78 | 0.93 |  | -0.01 | 0.93 | 0.98 |  |
| Lactobacillus | -0.03 | 0.75 | 0.89 |  | -0.05 | 0.59 | 0.84 |  | -0.10 | 0.29 | 0.77 |  | -0.14 | 0.13 | 0.62 |  | -0.13 | 0.16 | 0.70 |  | -0.14 | 0.14 | 0.75 |  |
| Lactococcus | 0.02 | 0.81 | 0.91 |  | 0.03 | 0.67 | 0.87 |  | 0.04 | 0.57 | 0.85 |  | 0.03 | 0.65 | 0.89 |  | 0.03 | 0.72 | 0.91 |  | 0.03 | 0.67 | 0.97 |  |
| Marvinbryantia | -0.20 | 0.07 | 0.30 |  | -0.22 | 0.06 | 0.36 |  | -0.22 | 0.06 | 0.49 |  | -0.22 | 0.06 | 0.44 |  | -0.22 | 0.07 | 0.46 |  | -0.22 | 0.06 | 0.63 |  |
| Megasphaera | -0.03 | 0.81 | 0.91 |  | -0.16 | 0.23 | 0.56 |  | -0.22 | 0.10 | 0.57 |  | -0.24 | 0.07 | 0.48 |  | -0.27 | 0.05 | 0.41 |  | -0.24 | 0.07 | 0.66 |  |
| Monoglobus | -0.06 | 0.52 | 0.75 |  | 0.001 | 0.99 | 1.00 |  | 0.05 | 0.55 | 0.85 |  | 0.05 | 0.55 | 0.86 |  | 0.07 | 0.46 | 0.84 |  | 0.06 | 0.53 | 0.90 |  |
| Moryella | -0.07 | 0.34 | 0.61 |  | -0.08 | 0.25 | 0.59 |  | -0.05 | 0.44 | 0.84 |  | -0.04 | 0.52 | 0.86 |  | -0.04 | 0.58 | 0.87 |  | -0.04 | 0.53 | 0.90 |  |
| Negativibacillus | 0.02 | 0.86 | 0.93 |  | 0.004 | 0.97 | 0.99 |  | -0.01 | 0.96 | 0.99 |  | -0.02 | 0.89 | 0.96 |  | -0.03 | 0.79 | 0.93 |  | -0.01 | 0.89 | 0.97 |  |
| NK4A214_group | -0.22 | 0.11 | 0.36 |  | -0.23 | 0.10 | 0.42 |  | -0.13 | 0.36 | 0.80 |  | -0.12 | 0.38 | 0.82 |  | -0.09 | 0.52 | 0.86 |  | -0.12 | 0.38 | 0.85 |  |
| Odoribacter | -0.13 | 0.35 | 0.62 |  | -0.19 | 0.19 | 0.55 |  | -0.12 | 0.40 | 0.83 |  | -0.11 | 0.44 | 0.84 |  | -0.10 | 0.50 | 0.86 |  | -0.11 | 0.45 | 0.89 |  |
| Oscillibacter | -0.05 | 0.50 | 0.75 |  | 0.01 | 0.86 | 0.94 |  | 0.06 | 0.49 | 0.84 |  | 0.06 | 0.47 | 0.85 |  | 0.06 | 0.46 | 0.84 |  | 0.06 | 0.45 | 0.89 |  |
| Oscillospira | 0.05 | 0.39 | 0.66 |  | 0.11 | 0.07 | 0.38 |  | 0.11 | 0.08 | 0.50 |  | 0.11 | 0.08 | 0.51 |  | 0.09 | 0.13 | 0.67 |  | 0.11 | 0.07 | 0.66 |  |
| Oxalobacter | -0.17 | 0.04 | 0.21 |  | -0.16 | 0.05 | 0.33 |  | -0.13 | 0.12 | 0.62 |  | -0.13 | 0.12 | 0.58 |  | -0.12 | 0.15 | 0.69 |  | -0.13 | 0.12 | 0.75 |  |
| Paludicola | -0.23 | 0.01 | 0.08 |  | -0.16 | 0.04 | 0.33 |  | -0.09 | 0.28 | 0.77 |  | -0.08 | 0.29 | 0.76 |  | -0.07 | 0.39 | 0.83 |  | -0.08 | 0.31 | 0.84 |  |
| Parabacteroides | 0.02 | 0.84 | 0.92 |  | 0.04 | 0.74 | 0.89 |  | -0.004 | 0.97 | 1.00 |  | -0.02 | 0.87 | 0.96 |  | -0.02 | 0.84 | 0.94 |  | -0.02 | 0.86 | 0.97 |  |
| Paraprevotella | 0.07 | 0.67 | 0.85 |  | -0.03 | 0.86 | 0.94 |  | 0.01 | 0.97 | 1.00 |  | 0.004 | 0.98 | 1.00 |  | -0.03 | 0.87 | 0.95 |  | -0.0002 | 1.00 | 1.00 |  |
| Parasutterella | -0.19 | 0.20 | 0.48 |  | -0.18 | 0.23 | 0.56 |  | -0.19 | 0.22 | 0.73 |  | -0.18 | 0.25 | 0.71 |  | -0.16 | 0.32 | 0.81 |  | -0.18 | 0.24 | 0.79 |  |
| Peptococcus | -0.06 | 0.40 | 0.67 |  | -0.08 | 0.31 | 0.63 |  | -0.04 | 0.63 | 0.85 |  | -0.03 | 0.66 | 0.89 |  | -0.03 | 0.72 | 0.91 |  | -0.03 | 0.68 | 0.97 |  |
| Phascolarctobacterium | -0.18 | 0.24 | 0.52 |  | -0.24 | 0.13 | 0.49 |  | -0.17 | 0.29 | 0.77 |  | -0.20 | 0.22 | 0.71 |  | -0.16 | 0.32 | 0.81 |  | -0.20 | 0.22 | 0.77 |  |
| Phocea | -0.01 | 0.89 | 0.94 |  | -0.02 | 0.79 | 0.92 |  | -0.06 | 0.32 | 0.78 |  | -0.07 | 0.31 | 0.77 |  | -0.08 | 0.24 | 0.77 |  | -0.07 | 0.30 | 0.84 |  |
| Prevotella | 0.02 | 0.91 | 0.95 |  | -0.13 | 0.44 | 0.72 |  | -0.12 | 0.48 | 0.84 |  | -0.12 | 0.50 | 0.85 |  | -0.12 | 0.51 | 0.86 |  | -0.12 | 0.50 | 0.89 |  |
| Romboutsia | -0.08 | 0.39 | 0.67 |  | -0.09 | 0.33 | 0.65 |  | -0.13 | 0.19 | 0.73 |  | -0.13 | 0.19 | 0.70 |  | -0.15 | 0.14 | 0.67 |  | -0.13 | 0.19 | 0.77 |  |
| Roseburia | 0.02 | 0.79 | 0.91 |  | 0.03 | 0.76 | 0.89 |  | 0.01 | 0.94 | 0.99 |  | 0.01 | 0.89 | 0.96 |  | 0.03 | 0.72 | 0.91 |  | 0.01 | 0.87 | 0.97 |  |
| Ruminococcus | -0.21 | 0.10 | 0.34 |  | -0.17 | 0.19 | 0.55 |  | -0.12 | 0.38 | 0.82 |  | -0.12 | 0.39 | 0.82 |  | -0.09 | 0.49 | 0.86 |  | -0.11 | 0.41 | 0.88 |  |
| Sellimonas | -0.16 | 0.07 | 0.30 |  | -0.15 | 0.09 | 0.41 |  | -0.10 | 0.28 | 0.77 |  | -0.10 | 0.30 | 0.76 |  | -0.11 | 0.25 | 0.77 |  | -0.10 | 0.31 | 0.84 |  |
| Senegalimassilia | -0.05 | 0.64 | 0.83 |  | -0.13 | 0.18 | 0.55 |  | -0.12 | 0.23 | 0.73 |  | -0.14 | 0.17 | 0.69 |  | -0.14 | 0.17 | 0.71 |  | -0.13 | 0.18 | 0.77 |  |
| Shuttleworthia | -0.12 | 0.18 | 0.46 |  | -0.03 | 0.74 | 0.89 |  | 0.03 | 0.76 | 0.91 |  | 0.03 | 0.73 | 0.91 |  | 0.04 | 0.64 | 0.88 |  | 0.03 | 0.74 | 0.97 |  |
| Slackia | -0.05 | 0.67 | 0.85 |  | -0.15 | 0.18 | 0.55 |  | -0.16 | 0.17 | 0.73 |  | -0.18 | 0.12 | 0.58 |  | -0.17 | 0.14 | 0.69 |  | -0.18 | 0.12 | 0.75 |  |
| Streptococcus | -0.004 | 0.96 | 0.97 |  | 0.01 | 0.91 | 0.96 |  | 0.02 | 0.79 | 0.92 |  | -0.02 | 0.78 | 0.92 |  | -0.02 | 0.84 | 0.94 |  | -0.02 | 0.76 | 0.97 |  |
| Subdoligranulum | -0.11 | 0.26 | 0.54 |  | -0.12 | 0.24 | 0.57 |  | -0.06 | 0.54 | 0.85 |  | -0.07 | 0.48 | 0.85 |  | -0.08 | 0.43 | 0.83 |  | -0.07 | 0.50 | 0.89 |  |
| Sutterella | -0.04 | 0.83 | 0.92 |  | -0.16 | 0.34 | 0.65 |  | -0.16 | 0.36 | 0.80 |  | -0.16 | 0.35 | 0.80 |  | -0.19 | 0.28 | 0.79 |  | -0.17 | 0.34 | 0.84 |  |
| TM7x | -0.04 | 0.56 | 0.78 |  | -0.03 | 0.67 | 0.87 |  | -0.04 | 0.60 | 0.85 |  | -0.05 | 0.54 | 0.86 |  | -0.04 | 0.55 | 0.87 |  | -0.05 | 0.54 | 0.90 |  |
| Turicibacter | 0.07 | 0.35 | 0.62 |  | 0.06 | 0.40 | 0.70 |  | 0.05 | 0.49 | 0.84 |  | 0.06 | 0.43 | 0.83 |  | 0.05 | 0.47 | 0.84 |  | 0.06 | 0.43 | 0.89 |  |
| Tuzzerella | 0.02 | 0.84 | 0.92 |  | 0.05 | 0.64 | 0.86 |  | 0.07 | 0.55 | 0.85 |  | 0.07 | 0.54 | 0.86 |  | 0.06 | 0.57 | 0.87 |  | 0.07 | 0.55 | 0.90 |  |
| UBA1819 | -0.08 | 0.23 | 0.51 |  | -0.02 | 0.76 | 0.89 |  | 0.02 | 0.80 | 0.92 |  | 0.02 | 0.80 | 0.92 |  | 0.01 | 0.85 | 0.94 |  | 0.02 | 0.79 | 0.97 |  |
| UCG.002 | -0.27 | 0.05 | 0.26 |  | -0.30 | 0.03 | 0.29 |  | -0.20 | 0.17 | 0.73 |  | -0.17 | 0.23 | 0.71 |  | -0.13 | 0.35 | 0.83 |  | -0.17 | 0.23 | 0.79 |  |
| UCG.003 | -0.11 | 0.38 | 0.65 |  | -0.18 | 0.14 | 0.49 |  | -0.15 | 0.24 | 0.73 |  | -0.14 | 0.27 | 0.73 |  | -0.14 | 0.30 | 0.79 |  | -0.14 | 0.26 | 0.82 |  |
| UCG.005 | -0.19 | 0.09 | 0.33 |  | -0.16 | 0.15 | 0.49 |  | -0.06 | 0.59 | 0.85 |  | -0.06 | 0.62 | 0.87 |  | -0.03 | 0.82 | 0.93 |  | -0.05 | 0.64 | 0.97 |  |
| UCG.009 | -0.01 | 0.90 | 0.94 |  | 0.01 | 0.92 | 0.96 |  | 0.04 | 0.58 | 0.85 |  | 0.05 | 0.55 | 0.86 |  | 0.05 | 0.50 | 0.86 |  | 0.05 | 0.52 | 0.90 |  |
| Veillonella | 0.002 | 0.98 | 0.98 |  | -0.04 | 0.71 | 0.88 |  | -0.03 | 0.77 | 0.91 |  | -0.06 | 0.56 | 0.86 |  | -0.06 | 0.58 | 0.87 |  | -0.06 | 0.55 | 0.90 |  |
| Victivallis | -0.09 | 0.40 | 0.67 |  | -0.13 | 0.19 | 0.55 |  | -0.07 | 0.50 | 0.84 |  | -0.07 | 0.49 | 0.85 |  | -0.07 | 0.49 | 0.85 |  | -0.07 | 0.50 | 0.89 |  |
| Data source: The Coronary Artery Risk Development in Young Adults (CARDIA), 1985-2016  The false discovery rate (FDR) was adjusted using Benjamini-Hochberg method.  Model 1adjusted for sequencing run. Model 2 additionally adjusted for age, sex, race, field center, and education. Model 3 additionally adjusted for physical activity, smoking status, alcohol use, and diet quality score. In Model 4, medication use, such as proton pump inhibitor and lipid-lowering, was added. Model 5 included all covariates in Model 4 and added systolic blood pressure, diastolic blood pressure, and antihypertensive medication (Y/N). Model 6 included all covariates in Model 4 and further adjusted for diabetes duration. | | | | | | | | | | | | | | | | | | | | | | | | |

| Additional file 1: Table S2d. The genus-level association between stages of diabetes (normal vs. diabetes without treatment) and specific taxa (n = 605, reference = normal) | | | | | | | | | | | | | | | | | | | | | | | | |
| --- | --- | --- | --- | --- | --- | --- | --- | --- | --- | --- | --- | --- | --- | --- | --- | --- | --- | --- | --- | --- | --- | --- | --- | --- |
|  | Model 1 | | |  | Model 2 | | |  | Model 3 | | |  | Model 4 | | |  | Model 5 | | |  | Model 6 | | | |
|  | β | p | FDR |  | β | p | FDR |  | β | p | FDR |  | β | p | FDR |  | β | p | FDR |  | β | p | FDR |  |
| Acetanaerobacterium | -0.09 | 0.31 | 0.59 |  | -0.07 | 0.41 | 0.70 |  | -0.06 | 0.52 | 0.85 |  | -0.06 | 0.53 | 0.86 |  | -0.06 | 0.50 | 0.86 |  | -0.05 | 0.64 | 0.97 |  |
| Acidaminococcus | 0.54 | 0.05 | 0.24 |  | 0.37 | 0.17 | 0.52 |  | 0.17 | 0.54 | 0.85 |  | 0.15 | 0.59 | 0.87 |  | 0.13 | 0.64 | 0.88 |  | 0.38 | 0.25 | 0.80 |  |
| Actinomyces | -0.07 | 0.48 | 0.73 |  | -0.12 | 0.24 | 0.57 |  | -0.16 | 0.12 | 0.62 |  | -0.18 | 0.07 | 0.48 |  | -0.19 | 0.06 | 0.44 |  | -0.17 | 0.16 | 0.75 |  |
| Adlercreutzia | -0.09 | 0.61 | 0.81 |  | 0.02 | 0.91 | 0.96 |  | 0.10 | 0.55 | 0.85 |  | 0.10 | 0.54 | 0.86 |  | 0.14 | 0.42 | 0.83 |  | -0.11 | 0.60 | 0.94 |  |
| Agathobacter | -0.01 | 0.95 | 0.97 |  | -0.05 | 0.69 | 0.87 |  | -0.04 | 0.76 | 0.91 |  | -0.03 | 0.80 | 0.92 |  | -0.04 | 0.75 | 0.93 |  | 0.02 | 0.91 | 0.97 |  |
| Akkermansia | -0.46 | 0.08 | 0.30 |  | -0.28 | 0.26 | 0.60 |  | -0.21 | 0.42 | 0.84 |  | -0.20 | 0.43 | 0.83 |  | -0.15 | 0.56 | 0.87 |  | -0.06 | 0.85 | 0.97 |  |
| Alistipes | -0.18 | 0.25 | 0.53 |  | -0.13 | 0.41 | 0.70 |  | -0.05 | 0.77 | 0.91 |  | -0.06 | 0.72 | 0.91 |  | -0.05 | 0.77 | 0.93 |  | 0.09 | 0.66 | 0.97 |  |
| Allisonella | 0.31 | 0.02 | 0.16 |  | 0.19 | 0.14 | 0.49 |  | 0.11 | 0.42 | 0.84 |  | 0.11 | 0.42 | 0.83 |  | 0.10 | 0.46 | 0.84 |  | 0.23 | 0.16 | 0.75 |  |
| Anaerofilum | -0.22 | 0.05 | 0.26 |  | -0.17 | 0.14 | 0.49 |  | -0.14 | 0.23 | 0.73 |  | -0.14 | 0.24 | 0.71 |  | -0.15 | 0.22 | 0.77 |  | -0.15 | 0.31 | 0.84 |  |
| Anaerostipes | -0.13 | 0.23 | 0.51 |  | -0.09 | 0.38 | 0.70 |  | -0.06 | 0.60 | 0.85 |  | -0.06 | 0.61 | 0.87 |  | -0.04 | 0.74 | 0.93 |  | -0.14 | 0.32 | 0.84 |  |
| Anaerotruncus | -0.30 | 0.02 | 0.15 |  | -0.23 | 0.06 | 0.38 |  | -0.17 | 0.18 | 0.73 |  | -0.17 | 0.18 | 0.70 |  | -0.16 | 0.21 | 0.77 |  | 0.02 | 0.88 | 0.97 |  |
| Bacteroides | -0.03 | 0.67 | 0.85 |  | 0.01 | 0.87 | 0.95 |  | -0.03 | 0.69 | 0.88 |  | -0.03 | 0.68 | 0.89 |  | -0.04 | 0.61 | 0.88 |  | -0.06 | 0.50 | 0.89 |  |
| Barnesiella | -0.42 | 0.12 | 0.38 |  | -0.38 | 0.16 | 0.51 |  | -0.36 | 0.20 | 0.73 |  | -0.37 | 0.19 | 0.70 |  | -0.35 | 0.21 | 0.77 |  | -0.23 | 0.50 | 0.89 |  |
| Bifidobacterium | 0.33 | 0.12 | 0.37 |  | 0.25 | 0.23 | 0.56 |  | 0.24 | 0.25 | 0.74 |  | 0.22 | 0.30 | 0.76 |  | 0.23 | 0.29 | 0.79 |  | 0.33 | 0.22 | 0.77 |  |
| Bilophila | 0.14 | 0.48 | 0.73 |  | 0.13 | 0.50 | 0.77 |  | 0.14 | 0.48 | 0.84 |  | 0.14 | 0.51 | 0.86 |  | 0.13 | 0.53 | 0.87 |  | 0.04 | 0.87 | 0.97 |  |
| Blautia | -0.08 | 0.17 | 0.44 |  | -0.06 | 0.30 | 0.63 |  | -0.06 | 0.32 | 0.78 |  | -0.06 | 0.31 | 0.77 |  | -0.06 | 0.34 | 0.82 |  | -0.07 | 0.32 | 0.84 |  |
| Butyricicoccus | -0.32 | <.001 | 0.01 |  | -0.27 | 0.001 | 0.05 |  | -0.26 | 0.002 | 0.10 |  | -0.26 | 0.002 | 0.13 |  | -0.25 | 0.004 | 0.20 |  | -0.29 | 0.01 | 0.32 |  |
| Butyricimonas | -0.25 | 0.29 | 0.57 |  | -0.31 | 0.19 | 0.55 |  | -0.27 | 0.25 | 0.74 |  | -0.29 | 0.23 | 0.71 |  | -0.25 | 0.30 | 0.79 |  | -0.42 | 0.15 | 0.75 |  |
| CAG.56 | -0.18 | 0.39 | 0.66 |  | -0.14 | 0.48 | 0.76 |  | -0.06 | 0.78 | 0.91 |  | -0.07 | 0.73 | 0.91 |  | -0.08 | 0.71 | 0.91 |  | 0.10 | 0.70 | 0.97 |  |
| Candidatus_Soleaferrea | -0.18 | 0.07 | 0.28 |  | -0.14 | 0.15 | 0.49 |  | -0.16 | 0.11 | 0.61 |  | -0.16 | 0.11 | 0.58 |  | -0.16 | 0.13 | 0.67 |  | 0.02 | 0.89 | 0.97 |  |
| Caproiciproducens | -0.28 | 0.02 | 0.14 |  | -0.24 | 0.04 | 0.32 |  | -0.24 | 0.05 | 0.43 |  | -0.24 | 0.05 | 0.42 |  | -0.22 | 0.07 | 0.48 |  | -0.14 | 0.35 | 0.84 |  |
| Christensenellaceae_R.7_group | -0.32 | 0.18 | 0.46 |  | -0.28 | 0.23 | 0.56 |  | -0.14 | 0.57 | 0.85 |  | -0.13 | 0.58 | 0.87 |  | -0.08 | 0.75 | 0.93 |  | 0.18 | 0.55 | 0.90 |  |
| Clostridium_sensu_stricto_1 | 0.39 | 0.04 | 0.21 |  | 0.42 | 0.02 | 0.24 |  | 0.49 | 0.01 | 0.20 |  | 0.48 | 0.01 | 0.20 |  | 0.48 | 0.01 | 0.22 |  | 0.46 | 0.05 | 0.61 |  |
| Colidextribacter | -0.27 | 0.005 | 0.07 |  | -0.23 | 0.01 | 0.17 |  | -0.26 | 0.01 | 0.19 |  | -0.26 | 0.01 | 0.18 |  | -0.28 | 0.01 | 0.20 |  | -0.15 | 0.22 | 0.77 |  |
| Collinsella | 0.23 | 0.28 | 0.56 |  | 0.13 | 0.54 | 0.80 |  | 0.07 | 0.76 | 0.91 |  | 0.04 | 0.85 | 0.94 |  | 0.05 | 0.84 | 0.94 |  | 0.17 | 0.52 | 0.90 |  |
| Coprobacter | -0.25 | 0.20 | 0.48 |  | -0.25 | 0.22 | 0.56 |  | -0.16 | 0.44 | 0.84 |  | -0.15 | 0.48 | 0.85 |  | -0.12 | 0.55 | 0.87 |  | -0.06 | 0.82 | 0.97 |  |
| Coprococcus | -0.05 | 0.80 | 0.91 |  | -0.09 | 0.63 | 0.85 |  | -0.09 | 0.63 | 0.85 |  | -0.09 | 0.63 | 0.89 |  | -0.09 | 0.64 | 0.88 |  | 0.18 | 0.43 | 0.89 |  |
| Defluviitaleaceae_UCG.011 | -0.16 | 0.19 | 0.47 |  | -0.08 | 0.50 | 0.77 |  | 0.01 | 0.94 | 0.99 |  | 0.02 | 0.89 | 0.96 |  | 0.05 | 0.68 | 0.90 |  | 0.24 | 0.13 | 0.75 |  |
| Desulfovibrio | -0.07 | 0.75 | 0.89 |  | -0.21 | 0.33 | 0.65 |  | -0.11 | 0.61 | 0.85 |  | -0.12 | 0.59 | 0.87 |  | -0.07 | 0.76 | 0.93 |  | -0.02 | 0.95 | 0.99 |  |
| Dialister | -0.25 | 0.37 | 0.65 |  | -0.23 | 0.41 | 0.71 |  | -0.16 | 0.57 | 0.85 |  | -0.15 | 0.61 | 0.87 |  | -0.22 | 0.44 | 0.83 |  | -0.07 | 0.84 | 0.97 |  |
| DNF00809 | -0.21 | 0.06 | 0.27 |  | -0.16 | 0.15 | 0.49 |  | -0.14 | 0.20 | 0.73 |  | -0.15 | 0.19 | 0.70 |  | -0.13 | 0.24 | 0.77 |  | 0.05 | 0.70 | 0.97 |  |
| Dorea | -0.06 | 0.68 | 0.85 |  | -0.10 | 0.46 | 0.75 |  | -0.12 | 0.43 | 0.84 |  | -0.11 | 0.45 | 0.84 |  | -0.12 | 0.42 | 0.83 |  | -0.18 | 0.32 | 0.84 |  |
| DTU089 | -0.24 | 0.08 | 0.30 |  | -0.18 | 0.18 | 0.55 |  | -0.17 | 0.23 | 0.73 |  | -0.17 | 0.23 | 0.71 |  | -0.17 | 0.23 | 0.77 |  | -0.04 | 0.81 | 0.97 |  |
| Eggerthella | -0.22 | 0.13 | 0.39 |  | -0.16 | 0.28 | 0.61 |  | -0.14 | 0.36 | 0.80 |  | -0.13 | 0.36 | 0.81 |  | -0.16 | 0.29 | 0.79 |  | -0.23 | 0.20 | 0.77 |  |
| Eisenbergiella | -0.13 | 0.35 | 0.62 |  | -0.06 | 0.66 | 0.86 |  | -0.001 | 0.99 | 1.00 |  | -0.01 | 0.93 | 0.98 |  | -0.02 | 0.91 | 0.97 |  | 0.06 | 0.74 | 0.97 |  |
| Erysipelatoclostridium | -0.09 | 0.48 | 0.73 |  | -0.04 | 0.77 | 0.90 |  | -0.07 | 0.62 | 0.85 |  | -0.06 | 0.68 | 0.89 |  | -0.07 | 0.62 | 0.88 |  | -0.02 | 0.89 | 0.97 |  |
| Erysipelotrichaceae_UCG.003 | -0.39 | 0.04 | 0.21 |  | -0.32 | 0.08 | 0.39 |  | -0.26 | 0.16 | 0.72 |  | -0.25 | 0.19 | 0.70 |  | -0.22 | 0.24 | 0.77 |  | -0.39 | 0.09 | 0.71 |  |
| Escherichia.Shigella | -0.01 | 0.94 | 0.96 |  | -0.02 | 0.90 | 0.95 |  | -0.08 | 0.70 | 0.88 |  | -0.09 | 0.65 | 0.89 |  | -0.13 | 0.52 | 0.86 |  | -0.07 | 0.78 | 0.97 |  |
| Faecalibacterium | -0.02 | 0.87 | 0.93 |  | -0.05 | 0.69 | 0.87 |  | 0.03 | 0.81 | 0.93 |  | 0.03 | 0.82 | 0.93 |  | 0.05 | 0.74 | 0.93 |  | 0.02 | 0.89 | 0.97 |  |
| Family_XIII_AD3011_group | -0.35 | 0.02 | 0.13 |  | -0.30 | 0.04 | 0.31 |  | -0.18 | 0.21 | 0.73 |  | -0.18 | 0.22 | 0.71 |  | -0.15 | 0.32 | 0.81 |  | 0.02 | 0.91 | 0.97 |  |
| Family_XIII_UCG.001 | -0.26 | 0.03 | 0.19 |  | -0.24 | 0.05 | 0.33 |  | -0.16 | 0.19 | 0.73 |  | -0.16 | 0.19 | 0.70 |  | -0.13 | 0.29 | 0.79 |  | -0.22 | 0.14 | 0.75 |  |
| FD2005 | -0.02 | 0.86 | 0.93 |  | -0.06 | 0.61 | 0.84 |  | -0.05 | 0.70 | 0.88 |  | -0.04 | 0.78 | 0.92 |  | -0.05 | 0.72 | 0.91 |  | 0.15 | 0.33 | 0.84 |  |
| Flavonifractor | -0.35 | 0.02 | 0.14 |  | -0.27 | 0.05 | 0.33 |  | -0.35 | 0.02 | 0.26 |  | -0.35 | 0.02 | 0.25 |  | -0.39 | 0.01 | 0.20 |  | -0.31 | 0.08 | 0.69 |  |
| Fournierella | -0.01 | 0.96 | 0.97 |  | -0.02 | 0.85 | 0.93 |  | 0.02 | 0.90 | 0.98 |  | 0.01 | 0.93 | 0.98 |  | 0.04 | 0.79 | 0.93 |  | 0.18 | 0.27 | 0.82 |  |
| Frisingicoccus | -0.22 | 0.21 | 0.48 |  | -0.11 | 0.52 | 0.78 |  | -0.003 | 0.99 | 1.00 |  | 0.002 | 0.99 | 1.00 |  | 0.02 | 0.92 | 0.97 |  | 0.24 | 0.27 | 0.82 |  |
| Fusicatenibacter | -0.07 | 0.71 | 0.87 |  | -0.08 | 0.67 | 0.87 |  | -0.06 | 0.73 | 0.89 |  | -0.05 | 0.79 | 0.92 |  | -0.04 | 0.81 | 0.93 |  | -0.10 | 0.65 | 0.97 |  |
| Fusobacterium | 0.12 | 0.32 | 0.60 |  | 0.10 | 0.41 | 0.71 |  | 0.06 | 0.65 | 0.87 |  | 0.06 | 0.66 | 0.89 |  | 0.03 | 0.79 | 0.93 |  | -0.10 | 0.53 | 0.90 |  |
| GCA.900066575 | -0.15 | 0.25 | 0.53 |  | -0.10 | 0.44 | 0.72 |  | 0.0003 | 1.00 | 1.00 |  | 0.01 | 0.94 | 0.99 |  | 0.01 | 0.95 | 0.98 |  | 0.01 | 0.96 | 0.99 |  |
| Gordonibacter | -0.26 | 0.05 | 0.26 |  | -0.15 | 0.24 | 0.57 |  | -0.08 | 0.54 | 0.85 |  | -0.07 | 0.61 | 0.87 |  | -0.06 | 0.68 | 0.90 |  | 0.03 | 0.85 | 0.97 |  |
| Granulicatella | 0.06 | 0.58 | 0.79 |  | 0.04 | 0.66 | 0.86 |  | 0.06 | 0.58 | 0.85 |  | 0.04 | 0.70 | 0.91 |  | 0.03 | 0.76 | 0.93 |  | -0.09 | 0.47 | 0.89 |  |
| Haemophilus | 0.21 | 0.25 | 0.53 |  | 0.18 | 0.31 | 0.63 |  | 0.33 | 0.07 | 0.50 |  | 0.29 | 0.11 | 0.58 |  | 0.30 | 0.09 | 0.54 |  | 0.21 | 0.34 | 0.84 |  |
| Harryflintia | -0.08 | 0.51 | 0.75 |  | -0.05 | 0.65 | 0.86 |  | -0.01 | 0.96 | 0.99 |  | -0.004 | 0.98 | 1.00 |  | -0.003 | 0.98 | 0.99 |  | 0.24 | 0.11 | 0.74 |  |
| Holdemanella | 0.25 | 0.18 | 0.46 |  | 0.13 | 0.47 | 0.76 |  | 0.07 | 0.69 | 0.88 |  | 0.07 | 0.71 | 0.91 |  | 0.07 | 0.69 | 0.90 |  | 0.28 | 0.21 | 0.77 |  |
| Holdemania | -0.10 | 0.20 | 0.48 |  | -0.07 | 0.36 | 0.67 |  | -0.05 | 0.54 | 0.85 |  | -0.05 | 0.56 | 0.86 |  | -0.05 | 0.56 | 0.87 |  | -0.01 | 0.91 | 0.97 |  |
| Hungatella | -0.27 | 0.06 | 0.28 |  | -0.22 | 0.13 | 0.49 |  | -0.28 | 0.06 | 0.49 |  | -0.29 | 0.05 | 0.42 |  | -0.30 | 0.04 | 0.40 |  | -0.33 | 0.07 | 0.66 |  |
| Hydrogenoanaerobacterium | -0.39 | 0.003 | 0.05 |  | -0.33 | 0.01 | 0.15 |  | -0.25 | 0.06 | 0.47 |  | -0.26 | 0.05 | 0.42 |  | -0.24 | 0.08 | 0.48 |  | -0.17 | 0.31 | 0.84 |  |
| Incertae_Sedis | -0.12 | 0.16 | 0.43 |  | -0.10 | 0.26 | 0.60 |  | -0.07 | 0.42 | 0.84 |  | -0.07 | 0.42 | 0.83 |  | -0.08 | 0.37 | 0.83 |  | -0.04 | 0.74 | 0.97 |  |
| Intestinibacter | -0.07 | 0.63 | 0.82 |  | -0.07 | 0.64 | 0.86 |  | -0.12 | 0.43 | 0.84 |  | -0.12 | 0.43 | 0.83 |  | -0.12 | 0.43 | 0.83 |  | -0.08 | 0.68 | 0.97 |  |
| Intestinimonas | -0.53 | 0.001 | 0.03 |  | -0.43 | 0.01 | 0.12 |  | -0.28 | 0.08 | 0.50 |  | -0.28 | 0.08 | 0.51 |  | -0.27 | 0.09 | 0.54 |  | -0.05 | 0.80 | 0.97 |  |
| Lachnoclostridium | -0.02 | 0.80 | 0.91 |  | -0.03 | 0.69 | 0.87 |  | -0.10 | 0.18 | 0.73 |  | -0.10 | 0.17 | 0.69 |  | -0.12 | 0.11 | 0.63 |  | -0.20 | 0.03 | 0.58 |  |
| Lachnospira | 0.16 | 0.30 | 0.58 |  | 0.22 | 0.14 | 0.49 |  | 0.30 | 0.05 | 0.44 |  | 0.30 | 0.05 | 0.42 |  | 0.34 | 0.03 | 0.32 |  | 0.51 | 0.01 | 0.32 |  |
| Lachnospiraceae_FCS020_group | -0.22 | 0.14 | 0.41 |  | -0.21 | 0.13 | 0.49 |  | -0.14 | 0.34 | 0.80 |  | -0.13 | 0.36 | 0.81 |  | -0.12 | 0.42 | 0.83 |  | -0.11 | 0.53 | 0.90 |  |
| Lachnospiraceae_ND3007_group | 0.02 | 0.92 | 0.95 |  | 0.03 | 0.90 | 0.95 |  | 0.04 | 0.87 | 0.96 |  | 0.05 | 0.83 | 0.93 |  | 0.06 | 0.79 | 0.93 |  | 0.21 | 0.44 | 0.89 |  |
| Lachnospiraceae_NK4A136_group | -0.18 | 0.23 | 0.51 |  | -0.14 | 0.34 | 0.65 |  | -0.03 | 0.87 | 0.96 |  | -0.02 | 0.89 | 0.96 |  | -0.003 | 0.98 | 0.99 |  | 0.23 | 0.20 | 0.77 |  |
| Lachnospiraceae_UCG.001 | -0.27 | 0.18 | 0.46 |  | -0.24 | 0.23 | 0.56 |  | -0.07 | 0.73 | 0.89 |  | -0.08 | 0.69 | 0.90 |  | -0.07 | 0.74 | 0.93 |  | 0.09 | 0.72 | 0.97 |  |
| Lachnospiraceae_UCG.004 | 0.12 | 0.52 | 0.75 |  | 0.10 | 0.60 | 0.84 |  | 0.18 | 0.35 | 0.80 |  | 0.17 | 0.37 | 0.81 |  | 0.14 | 0.47 | 0.84 |  | 0.03 | 0.89 | 0.97 |  |
| Lachnospiraceae_UCG.010 | -0.08 | 0.54 | 0.77 |  | -0.07 | 0.57 | 0.82 |  | -0.02 | 0.89 | 0.97 |  | -0.02 | 0.90 | 0.96 |  | -0.03 | 0.80 | 0.93 |  | -0.03 | 0.87 | 0.97 |  |
| Lactobacillus | 0.04 | 0.78 | 0.91 |  | -0.002 | 0.99 | 1.00 |  | -0.10 | 0.53 | 0.85 |  | -0.14 | 0.37 | 0.82 |  | -0.14 | 0.38 | 0.83 |  | 0.003 | 0.99 | 1.00 |  |
| Lactococcus | -0.13 | 0.27 | 0.55 |  | -0.11 | 0.35 | 0.66 |  | -0.07 | 0.55 | 0.85 |  | -0.08 | 0.50 | 0.86 |  | -0.09 | 0.47 | 0.84 |  | -0.16 | 0.30 | 0.84 |  |
| Marvinbryantia | -0.50 | 0.01 | 0.09 |  | -0.48 | 0.01 | 0.16 |  | -0.50 | 0.01 | 0.20 |  | -0.50 | 0.01 | 0.20 |  | -0.49 | 0.01 | 0.22 |  | -0.44 | 0.07 | 0.66 |  |
| Megasphaera | 0.34 | 0.15 | 0.42 |  | 0.15 | 0.49 | 0.76 |  | 0.06 | 0.78 | 0.91 |  | 0.04 | 0.84 | 0.94 |  | -0.01 | 0.97 | 0.99 |  | -0.10 | 0.71 | 0.97 |  |
| Monoglobus | 0.12 | 0.45 | 0.71 |  | 0.22 | 0.14 | 0.49 |  | 0.34 | 0.02 | 0.31 |  | 0.34 | 0.02 | 0.30 |  | 0.37 | 0.02 | 0.23 |  | 0.53 | 0.00 | 0.32 |  |
| Moryella | -0.16 | 0.15 | 0.42 |  | -0.15 | 0.18 | 0.55 |  | -0.08 | 0.51 | 0.84 |  | -0.07 | 0.56 | 0.86 |  | -0.06 | 0.60 | 0.88 |  | 0.00004 | 1.00 | 1.00 |  |
| Negativibacillus | 0.07 | 0.70 | 0.87 |  | 0.07 | 0.68 | 0.87 |  | 0.05 | 0.81 | 0.93 |  | 0.03 | 0.86 | 0.95 |  | 0.01 | 0.97 | 0.99 |  | 0.04 | 0.86 | 0.97 |  |
| NK4A214_group | -0.41 | 0.07 | 0.28 |  | -0.39 | 0.08 | 0.39 |  | -0.25 | 0.29 | 0.77 |  | -0.25 | 0.29 | 0.75 |  | -0.21 | 0.37 | 0.83 |  | -0.13 | 0.64 | 0.97 |  |
| Odoribacter | -0.18 | 0.45 | 0.71 |  | -0.21 | 0.37 | 0.68 |  | -0.10 | 0.69 | 0.88 |  | -0.09 | 0.72 | 0.91 |  | -0.08 | 0.75 | 0.93 |  | 0.12 | 0.68 | 0.97 |  |
| Oscillibacter | -0.45 | 0.001 | 0.02 |  | -0.37 | 0.01 | 0.11 |  | -0.27 | 0.05 | 0.43 |  | -0.27 | 0.05 | 0.42 |  | -0.27 | 0.05 | 0.44 |  | -0.05 | 0.75 | 0.97 |  |
| Oscillospira | -0.10 | 0.32 | 0.60 |  | -0.04 | 0.71 | 0.88 |  | -0.04 | 0.67 | 0.88 |  | -0.04 | 0.68 | 0.89 |  | -0.06 | 0.56 | 0.87 |  | 0.12 | 0.32 | 0.84 |  |
| Oxalobacter | -0.33 | 0.01 | 0.13 |  | -0.30 | 0.03 | 0.26 |  | -0.21 | 0.12 | 0.62 |  | -0.22 | 0.12 | 0.58 |  | -0.18 | 0.20 | 0.77 |  | -0.12 | 0.49 | 0.89 |  |
| Paludicola | -0.50 | <.001 | 0.01 |  | -0.40 | 0.002 | 0.08 |  | -0.29 | 0.03 | 0.34 |  | -0.29 | 0.03 | 0.33 |  | -0.27 | 0.04 | 0.41 |  | -0.05 | 0.76 | 0.97 |  |
| Parabacteroides | -0.09 | 0.63 | 0.82 |  | -0.09 | 0.63 | 0.85 |  | -0.18 | 0.34 | 0.80 |  | -0.20 | 0.30 | 0.77 |  | -0.20 | 0.30 | 0.79 |  | -0.32 | 0.19 | 0.77 |  |
| Paraprevotella | 0.44 | 0.08 | 0.32 |  | 0.34 | 0.19 | 0.55 |  | 0.34 | 0.19 | 0.73 |  | 0.34 | 0.20 | 0.70 |  | 0.28 | 0.28 | 0.79 |  | 0.08 | 0.81 | 0.97 |  |
| Parasutterella | 0.05 | 0.85 | 0.92 |  | 0.08 | 0.76 | 0.89 |  | 0.13 | 0.60 | 0.85 |  | 0.14 | 0.58 | 0.87 |  | 0.20 | 0.43 | 0.83 |  | -0.08 | 0.79 | 0.97 |  |
| Peptococcus | -0.18 | 0.15 | 0.42 |  | -0.18 | 0.16 | 0.51 |  | -0.09 | 0.48 | 0.84 |  | -0.09 | 0.49 | 0.85 |  | -0.08 | 0.54 | 0.87 |  | 0.03 | 0.84 | 0.97 |  |
| Phascolarctobacterium | 0.33 | 0.21 | 0.48 |  | 0.28 | 0.29 | 0.63 |  | 0.30 | 0.27 | 0.77 |  | 0.27 | 0.31 | 0.77 |  | 0.34 | 0.21 | 0.77 |  | 0.35 | 0.29 | 0.84 |  |
| Phocea | -0.01 | 0.89 | 0.94 |  | -0.02 | 0.88 | 0.95 |  | -0.06 | 0.56 | 0.85 |  | -0.06 | 0.56 | 0.86 |  | -0.08 | 0.45 | 0.84 |  | -0.20 | 0.12 | 0.75 |  |
| Prevotella | 0.49 | 0.09 | 0.33 |  | 0.30 | 0.29 | 0.63 |  | 0.26 | 0.38 | 0.82 |  | 0.26 | 0.38 | 0.82 |  | 0.28 | 0.35 | 0.83 |  | 0.34 | 0.35 | 0.84 |  |
| Romboutsia | -0.06 | 0.72 | 0.87 |  | -0.08 | 0.62 | 0.84 |  | -0.12 | 0.45 | 0.84 |  | -0.12 | 0.45 | 0.84 |  | -0.14 | 0.39 | 0.83 |  | -0.15 | 0.47 | 0.89 |  |
| Roseburia | 0.12 | 0.41 | 0.68 |  | 0.14 | 0.33 | 0.65 |  | 0.15 | 0.30 | 0.78 |  | 0.15 | 0.29 | 0.75 |  | 0.17 | 0.23 | 0.77 |  | 0.31 | 0.08 | 0.69 |  |
| Ruminococcus | -0.07 | 0.74 | 0.88 |  | 0.02 | 0.94 | 0.97 |  | 0.16 | 0.47 | 0.84 |  | 0.16 | 0.47 | 0.85 |  | 0.20 | 0.37 | 0.83 |  | 0.55 | 0.04 | 0.59 |  |
| Sellimonas | -0.29 | 0.05 | 0.26 |  | -0.27 | 0.07 | 0.38 |  | -0.19 | 0.23 | 0.73 |  | -0.19 | 0.24 | 0.71 |  | -0.20 | 0.20 | 0.77 |  | -0.04 | 0.84 | 0.97 |  |
| Senegalimassilia | -0.09 | 0.58 | 0.79 |  | -0.17 | 0.29 | 0.62 |  | -0.13 | 0.42 | 0.84 |  | -0.15 | 0.37 | 0.82 |  | -0.14 | 0.40 | 0.83 |  | 0.14 | 0.48 | 0.89 |  |
| Shuttleworthia | -0.19 | 0.22 | 0.50 |  | -0.05 | 0.75 | 0.89 |  | 0.07 | 0.64 | 0.86 |  | 0.07 | 0.63 | 0.89 |  | 0.07 | 0.63 | 0.88 |  | -0.01 | 0.94 | 0.98 |  |
| Slackia | 0.03 | 0.86 | 0.93 |  | -0.07 | 0.68 | 0.87 |  | -0.07 | 0.70 | 0.88 |  | -0.09 | 0.64 | 0.89 |  | -0.07 | 0.71 | 0.91 |  | -0.09 | 0.71 | 0.97 |  |
| Streptococcus | -0.05 | 0.70 | 0.87 |  | -0.05 | 0.71 | 0.88 |  | -0.01 | 0.92 | 0.98 |  | -0.06 | 0.65 | 0.89 |  | -0.06 | 0.66 | 0.89 |  | -0.14 | 0.34 | 0.84 |  |
| Subdoligranulum | -0.05 | 0.75 | 0.89 |  | -0.04 | 0.83 | 0.93 |  | 0.07 | 0.70 | 0.88 |  | 0.06 | 0.74 | 0.91 |  | 0.05 | 0.77 | 0.93 |  | 0.30 | 0.16 | 0.75 |  |
| Sutterella | 0.13 | 0.66 | 0.84 |  | -0.06 | 0.84 | 0.93 |  | -0.10 | 0.72 | 0.89 |  | -0.10 | 0.72 | 0.91 |  | -0.13 | 0.65 | 0.88 |  | -0.19 | 0.59 | 0.94 |  |
| TM7x | -0.12 | 0.32 | 0.60 |  | -0.10 | 0.43 | 0.72 |  | -0.07 | 0.57 | 0.85 |  | -0.08 | 0.54 | 0.86 |  | -0.07 | 0.57 | 0.87 |  | -0.09 | 0.55 | 0.90 |  |
| Turicibacter | 0.02 | 0.86 | 0.93 |  | 0.001 | 0.99 | 1.00 |  | -0.01 | 0.94 | 0.99 |  | -0.003 | 0.98 | 1.00 |  | -0.01 | 0.92 | 0.97 |  | 0.01 | 0.93 | 0.98 |  |
| Tuzzerella | -0.31 | 0.08 | 0.32 |  | -0.26 | 0.13 | 0.49 |  | -0.26 | 0.15 | 0.70 |  | -0.26 | 0.15 | 0.65 |  | -0.26 | 0.15 | 0.69 |  | -0.33 | 0.13 | 0.75 |  |
| UBA1819 | -0.13 | 0.26 | 0.54 |  | -0.06 | 0.59 | 0.84 |  | -0.01 | 0.93 | 0.99 |  | -0.01 | 0.93 | 0.98 |  | -0.03 | 0.82 | 0.93 |  | 0.09 | 0.54 | 0.90 |  |
| UCG.002 | -0.34 | 0.14 | 0.41 |  | -0.34 | 0.15 | 0.49 |  | -0.17 | 0.48 | 0.84 |  | -0.15 | 0.53 | 0.86 |  | -0.10 | 0.69 | 0.90 |  | 0.07 | 0.82 | 0.97 |  |
| UCG.003 | -0.02 | 0.93 | 0.95 |  | -0.07 | 0.74 | 0.89 |  | 0.002 | 0.99 | 1.00 |  | 0.01 | 0.96 | 1.00 |  | 0.02 | 0.92 | 0.97 |  | -0.08 | 0.76 | 0.97 |  |
| UCG.005 | -0.29 | 0.12 | 0.38 |  | -0.23 | 0.21 | 0.56 |  | -0.07 | 0.72 | 0.89 |  | -0.06 | 0.74 | 0.91 |  | -0.01 | 0.94 | 0.97 |  | 0.21 | 0.37 | 0.85 |  |
| UCG.009 | -0.19 | 0.13 | 0.38 |  | -0.15 | 0.23 | 0.56 |  | -0.08 | 0.50 | 0.84 |  | -0.08 | 0.52 | 0.86 |  | -0.07 | 0.58 | 0.87 |  | 0.16 | 0.31 | 0.84 |  |
| Veillonella | 0.27 | 0.12 | 0.38 |  | 0.23 | 0.19 | 0.55 |  | 0.27 | 0.12 | 0.62 |  | 0.24 | 0.17 | 0.69 |  | 0.23 | 0.18 | 0.75 |  | 0.15 | 0.49 | 0.89 |  |
| Victivallis | -0.29 | 0.10 | 0.34 |  | -0.33 | 0.05 | 0.33 |  | -0.21 | 0.23 | 0.73 |  | -0.21 | 0.22 | 0.71 |  | -0.21 | 0.24 | 0.77 |  | -0.10 | 0.64 | 0.97 |  |
| Data source: The Coronary Artery Risk Development in Young Adults (CARDIA), 1985-2016  The false discovery rate (FDR) was adjusted using Benjamini-Hochberg method.  Model 1adjusted for sequencing run. Model 2 additionally adjusted for age, sex, race, field center, and education. Model 3 additionally adjusted for physical activity, smoking status, alcohol use, and diet quality score. In Model 4, medication use, such as proton pump inhibitor and lipid-lowering, was added. Model 5 included all covariates in Model 4 and added systolic blood pressure, diastolic blood pressure, and antihypertensive medication (Y/N). Model 6 included all covariates in Model 4 and further adjusted for diabetes duration. | | | | | | | | | | | | | | | | | | | | | | | | |

| Additional file 1: Table S2e. The genus-level association between stages of diabetes (normal vs. diabetes with treatment) and specific taxa (n = 605, reference = normal) | | | | | | | | | | | | | | | | | | | | | | | | |
| --- | --- | --- | --- | --- | --- | --- | --- | --- | --- | --- | --- | --- | --- | --- | --- | --- | --- | --- | --- | --- | --- | --- | --- | --- |
|  | Model 1 | | |  | Model 2 | | |  | Model 3 | | |  | Model 4 | | |  | Model 5 | | |  | Model 6 | | | |
|  | β | p | FDR |  | β | p | FDR |  | β | p | FDR |  | β | p | FDR |  | β | p | FDR |  | β | p | FDR |  |
| Acetanaerobacterium | -0.05 | 0.43 | 0.69 |  | -0.07 | 0.34 | 0.65 |  | -0.05 | 0.45 | 0.84 |  | -0.06 | 0.39 | 0.83 |  | -0.06 | 0.41 | 0.83 |  | -0.06 | 0.58 | 0.93 |  |
| Acidaminococcus | 0.29 | 0.16 | 0.43 |  | 0.17 | 0.40 | 0.70 |  | 0.02 | 0.91 | 0.98 |  | 0.01 | 0.97 | 1.00 |  | -0.02 | 0.91 | 0.97 |  | 0.27 | 0.38 | 0.85 |  |
| Actinomyces | 0.12 | 0.12 | 0.37 |  | 0.03 | 0.72 | 0.88 |  | -0.01 | 0.90 | 0.97 |  | -0.05 | 0.49 | 0.85 |  | -0.06 | 0.48 | 0.84 |  | -0.05 | 0.67 | 0.97 |  |
| Adlercreutzia | -0.11 | 0.41 | 0.68 |  | 0.02 | 0.88 | 0.95 |  | 0.08 | 0.52 | 0.85 |  | 0.04 | 0.77 | 0.92 |  | 0.07 | 0.59 | 0.88 |  | -0.19 | 0.31 | 0.84 |  |
| Agathobacter | -0.27 | 0.01 | 0.08 |  | -0.30 | 0.003 | 0.08 |  | -0.34 | 0.001 | 0.08 |  | -0.36 | 0.001 | 0.05 |  | -0.36 | 0.001 | 0.06 |  | -0.31 | 0.04 | 0.59 |  |
| Akkermansia | -0.32 | 0.11 | 0.36 |  | -0.14 | 0.48 | 0.76 |  | -0.13 | 0.54 | 0.85 |  | -0.05 | 0.80 | 0.92 |  | -0.02 | 0.92 | 0.97 |  | 0.10 | 0.72 | 0.97 |  |
| Alistipes | -0.09 | 0.42 | 0.69 |  | -0.06 | 0.62 | 0.84 |  | -0.01 | 0.91 | 0.98 |  | -0.02 | 0.85 | 0.94 |  | -0.02 | 0.89 | 0.96 |  | 0.13 | 0.46 | 0.89 |  |
| Allisonella | 0.18 | 0.09 | 0.33 |  | 0.09 | 0.35 | 0.66 |  | 0.07 | 0.48 | 0.84 |  | 0.05 | 0.61 | 0.87 |  | 0.05 | 0.62 | 0.88 |  | 0.19 | 0.20 | 0.77 |  |
| Anaerofilum | -0.14 | 0.11 | 0.37 |  | -0.14 | 0.11 | 0.44 |  | -0.11 | 0.23 | 0.73 |  | -0.12 | 0.21 | 0.70 |  | -0.14 | 0.14 | 0.67 |  | -0.13 | 0.34 | 0.84 |  |
| Anaerostipes | -0.25 | 0.003 | 0.05 |  | -0.21 | 0.01 | 0.17 |  | -0.20 | 0.02 | 0.28 |  | -0.22 | 0.01 | 0.25 |  | -0.21 | 0.02 | 0.28 |  | -0.31 | 0.01 | 0.44 |  |
| Anaerotruncus | 0.06 | 0.56 | 0.79 |  | 0.04 | 0.70 | 0.88 |  | 0.09 | 0.36 | 0.80 |  | 0.07 | 0.49 | 0.85 |  | 0.07 | 0.50 | 0.86 |  | 0.28 | 0.05 | 0.59 |  |
| Bacteroides | 0.02 | 0.78 | 0.91 |  | 0.06 | 0.31 | 0.63 |  | 0.04 | 0.51 | 0.84 |  | 0.05 | 0.36 | 0.81 |  | 0.05 | 0.42 | 0.83 |  | 0.02 | 0.80 | 0.97 |  |
| Barnesiella | -0.67 | 0.001 | 0.03 |  | -0.59 | 0.01 | 0.11 |  | -0.56 | 0.01 | 0.20 |  | -0.60 | 0.01 | 0.18 |  | -0.60 | 0.01 | 0.20 |  | -0.45 | 0.15 | 0.75 |  |
| Bifidobacterium | -0.13 | 0.42 | 0.68 |  | -0.21 | 0.19 | 0.55 |  | -0.26 | 0.12 | 0.62 |  | -0.28 | 0.10 | 0.56 |  | -0.28 | 0.10 | 0.58 |  | -0.17 | 0.48 | 0.89 |  |
| Bilophila | -0.03 | 0.85 | 0.92 |  | -0.03 | 0.87 | 0.94 |  | -0.03 | 0.86 | 0.96 |  | -0.04 | 0.83 | 0.93 |  | -0.07 | 0.68 | 0.90 |  | -0.14 | 0.55 | 0.90 |  |
| Blautia | -0.01 | 0.91 | 0.95 |  | 0.02 | 0.62 | 0.84 |  | 0.02 | 0.59 | 0.85 |  | 0.02 | 0.75 | 0.92 |  | 0.003 | 0.95 | 0.98 |  | 0.001 | 0.98 | 1.00 |  |
| Butyricicoccus | -0.17 | 0.01 | 0.09 |  | -0.12 | 0.07 | 0.38 |  | -0.12 | 0.08 | 0.50 |  | -0.10 | 0.13 | 0.62 |  | -0.10 | 0.15 | 0.69 |  | -0.14 | 0.15 | 0.75 |  |
| Butyricimonas | 0.21 | 0.24 | 0.52 |  | 0.18 | 0.33 | 0.65 |  | 0.19 | 0.30 | 0.78 |  | 0.17 | 0.39 | 0.82 |  | 0.16 | 0.40 | 0.83 |  | 0.02 | 0.94 | 0.98 |  |
| CAG.56 | -0.15 | 0.33 | 0.61 |  | -0.09 | 0.59 | 0.84 |  | -0.07 | 0.67 | 0.88 |  | -0.09 | 0.60 | 0.87 |  | -0.10 | 0.54 | 0.87 |  | 0.10 | 0.67 | 0.97 |  |
| Candidatus_Soleaferrea | -0.11 | 0.14 | 0.41 |  | -0.10 | 0.22 | 0.56 |  | -0.09 | 0.25 | 0.74 |  | -0.10 | 0.25 | 0.71 |  | -0.09 | 0.29 | 0.79 |  | 0.10 | 0.38 | 0.85 |  |
| Caproiciproducens | -0.10 | 0.26 | 0.54 |  | -0.09 | 0.33 | 0.65 |  | -0.08 | 0.40 | 0.83 |  | -0.10 | 0.33 | 0.78 |  | -0.08 | 0.41 | 0.83 |  | 0.01 | 0.94 | 0.98 |  |
| Christensenellaceae_R.7_group | -0.46 | 0.01 | 0.11 |  | -0.46 | 0.01 | 0.16 |  | -0.33 | 0.08 | 0.50 |  | -0.36 | 0.06 | 0.44 |  | -0.32 | 0.10 | 0.58 |  | -0.02 | 0.94 | 0.98 |  |
| Clostridium_sensu_stricto_1 | -0.49 | 0.001 | 0.02 |  | -0.38 | 0.01 | 0.14 |  | -0.36 | 0.02 | 0.26 |  | -0.33 | 0.03 | 0.34 |  | -0.37 | 0.02 | 0.23 |  | -0.36 | 0.09 | 0.71 |  |
| Colidextribacter | -0.10 | 0.18 | 0.46 |  | -0.07 | 0.32 | 0.65 |  | -0.07 | 0.38 | 0.82 |  | -0.09 | 0.26 | 0.73 |  | -0.10 | 0.22 | 0.77 |  | 0.04 | 0.74 | 0.97 |  |
| Collinsella | -0.11 | 0.50 | 0.75 |  | -0.14 | 0.40 | 0.70 |  | -0.21 | 0.23 | 0.73 |  | -0.20 | 0.26 | 0.73 |  | -0.19 | 0.29 | 0.79 |  | -0.06 | 0.82 | 0.97 |  |
| Coprobacter | -0.31 | 0.04 | 0.22 |  | -0.22 | 0.15 | 0.49 |  | -0.15 | 0.33 | 0.80 |  | -0.14 | 0.41 | 0.83 |  | -0.10 | 0.54 | 0.87 |  | -0.04 | 0.87 | 0.97 |  |
| Coprococcus | -0.24 | 0.09 | 0.33 |  | -0.25 | 0.07 | 0.38 |  | -0.25 | 0.08 | 0.50 |  | -0.25 | 0.09 | 0.56 |  | -0.29 | 0.05 | 0.44 |  | 0.04 | 0.83 | 0.97 |  |
| Defluviitaleaceae_UCG.011 | -0.06 | 0.53 | 0.76 |  | -0.004 | 0.97 | 0.99 |  | 0.06 | 0.56 | 0.85 |  | 0.04 | 0.66 | 0.89 |  | 0.08 | 0.43 | 0.83 |  | 0.29 | 0.05 | 0.59 |  |
| Desulfovibrio | 0.28 | 0.10 | 0.34 |  | 0.19 | 0.27 | 0.60 |  | 0.28 | 0.10 | 0.59 |  | 0.26 | 0.15 | 0.65 |  | 0.31 | 0.08 | 0.50 |  | 0.37 | 0.14 | 0.75 |  |
| Dialister | -0.11 | 0.59 | 0.79 |  | -0.08 | 0.72 | 0.88 |  | -0.13 | 0.56 | 0.85 |  | -0.18 | 0.44 | 0.84 |  | -0.18 | 0.43 | 0.83 |  | -0.10 | 0.77 | 0.97 |  |
| DNF00809 | -0.18 | 0.04 | 0.21 |  | -0.15 | 0.09 | 0.39 |  | -0.12 | 0.18 | 0.73 |  | -0.13 | 0.15 | 0.65 |  | -0.11 | 0.22 | 0.77 |  | 0.09 | 0.49 | 0.89 |  |
| Dorea | 0.02 | 0.84 | 0.92 |  | 0.03 | 0.79 | 0.91 |  | 0.004 | 0.97 | 1.00 |  | -0.003 | 0.98 | 1.00 |  | -0.02 | 0.86 | 0.95 |  | -0.08 | 0.64 | 0.97 |  |
| DTU089 | -0.04 | 0.72 | 0.87 |  | 0.02 | 0.84 | 0.93 |  | 0.05 | 0.66 | 0.87 |  | 0.05 | 0.67 | 0.89 |  | 0.06 | 0.61 | 0.88 |  | 0.18 | 0.24 | 0.79 |  |
| Eggerthella | 0.05 | 0.65 | 0.84 |  | 0.04 | 0.72 | 0.88 |  | 0.05 | 0.64 | 0.86 |  | 0.06 | 0.60 | 0.87 |  | 0.03 | 0.81 | 0.93 |  | -0.05 | 0.77 | 0.97 |  |
| Eisenbergiella | 0.04 | 0.70 | 0.87 |  | 0.02 | 0.88 | 0.95 |  | 0.08 | 0.45 | 0.84 |  | 0.03 | 0.79 | 0.92 |  | 0.01 | 0.91 | 0.97 |  | 0.11 | 0.50 | 0.89 |  |
| Erysipelatoclostridium | -0.07 | 0.52 | 0.75 |  | -0.04 | 0.68 | 0.87 |  | -0.01 | 0.92 | 0.98 |  | -0.0003 | 1.00 | 1.00 |  | 0.0002 | 1.00 | 1.00 |  | 0.04 | 0.81 | 0.97 |  |
| Erysipelotrichaceae_UCG.003 | -0.65 | <.001 | <.001 |  | -0.51 | <.001 | 0.03 |  | -0.46 | 0.002 | 0.09 |  | -0.40 | 0.01 | 0.18 |  | -0.37 | 0.01 | 0.22 |  | -0.56 | 0.01 | 0.32 |  |
| Escherichia.Shigella | 0.95 | <.001 | <.001 |  | 0.91 | <.001 | <.001 |  | 0.85 | <.001 | <.001 |  | 0.83 | <.001 | <.001 |  | 0.78 | <.001 | <.001 |  | 0.85 | 0.00 | 0.03 |  |
| Faecalibacterium | -0.35 | 0.001 | 0.02 |  | -0.39 | <.001 | 0.03 |  | -0.36 | 0.001 | 0.08 |  | -0.42 | <.001 | 0.01 |  | -0.43 | <.001 | 0.01 |  | -0.43 | 0.01 | 0.32 |  |
| Family_XIII_AD3011_group | -0.26 | 0.02 | 0.14 |  | -0.23 | 0.04 | 0.31 |  | -0.15 | 0.20 | 0.73 |  | -0.18 | 0.14 | 0.63 |  | -0.15 | 0.20 | 0.77 |  | 0.05 | 0.78 | 0.97 |  |
| Family_XIII_UCG.001 | -0.26 | 0.005 | 0.07 |  | -0.19 | 0.04 | 0.32 |  | -0.13 | 0.16 | 0.72 |  | -0.15 | 0.12 | 0.58 |  | -0.14 | 0.16 | 0.70 |  | -0.22 | 0.11 | 0.74 |  |
| FD2005 | 0.03 | 0.72 | 0.87 |  | 0.02 | 0.84 | 0.93 |  | 0.01 | 0.96 | 0.99 |  | -0.01 | 0.96 | 1.00 |  | -0.004 | 0.97 | 0.99 |  | 0.20 | 0.16 | 0.75 |  |
| Flavonifractor | 0.02 | 0.84 | 0.92 |  | 0.02 | 0.84 | 0.93 |  | -0.001 | 0.99 | 1.00 |  | -0.001 | 0.99 | 1.00 |  | -0.03 | 0.83 | 0.94 |  | 0.04 | 0.81 | 0.97 |  |
| Fournierella | -0.22 | 0.02 | 0.16 |  | -0.22 | 0.03 | 0.26 |  | -0.19 | 0.07 | 0.49 |  | -0.22 | 0.04 | 0.37 |  | -0.19 | 0.07 | 0.48 |  | -0.04 | 0.80 | 0.97 |  |
| Frisingicoccus | 0.14 | 0.30 | 0.58 |  | 0.20 | 0.14 | 0.49 |  | 0.27 | 0.04 | 0.43 |  | 0.20 | 0.15 | 0.65 |  | 0.20 | 0.16 | 0.70 |  | 0.46 | 0.02 | 0.44 |  |
| Fusicatenibacter | -0.40 | 0.004 | 0.07 |  | -0.38 | 0.01 | 0.12 |  | -0.42 | 0.003 | 0.13 |  | -0.43 | 0.004 | 0.16 |  | -0.42 | 0.01 | 0.20 |  | -0.49 | 0.02 | 0.44 |  |
| Fusobacterium | 0.24 | 0.01 | 0.10 |  | 0.23 | 0.02 | 0.21 |  | 0.23 | 0.02 | 0.28 |  | 0.24 | 0.02 | 0.25 |  | 0.23 | 0.03 | 0.30 |  | 0.07 | 0.61 | 0.96 |  |
| GCA.900066575 | -0.33 | 0.001 | 0.03 |  | -0.29 | 0.005 | 0.10 |  | -0.21 | 0.05 | 0.43 |  | -0.20 | 0.06 | 0.44 |  | -0.20 | 0.07 | 0.46 |  | -0.20 | 0.18 | 0.77 |  |
| Gordonibacter | -0.08 | 0.41 | 0.68 |  | 0.002 | 0.98 | 0.99 |  | 0.07 | 0.47 | 0.84 |  | 0.09 | 0.41 | 0.83 |  | 0.09 | 0.39 | 0.83 |  | 0.19 | 0.19 | 0.77 |  |
| Granulicatella | 0.11 | 0.15 | 0.42 |  | 0.02 | 0.76 | 0.89 |  | 0.02 | 0.83 | 0.94 |  | -0.01 | 0.90 | 0.96 |  | -0.02 | 0.84 | 0.94 |  | -0.15 | 0.18 | 0.77 |  |
| Haemophilus | -0.14 | 0.32 | 0.60 |  | -0.21 | 0.14 | 0.49 |  | -0.17 | 0.24 | 0.73 |  | -0.24 | 0.09 | 0.54 |  | -0.25 | 0.08 | 0.50 |  | -0.33 | 0.10 | 0.72 |  |
| Harryflintia | 0.08 | 0.36 | 0.64 |  | 0.08 | 0.40 | 0.70 |  | 0.09 | 0.33 | 0.80 |  | 0.08 | 0.40 | 0.83 |  | 0.08 | 0.41 | 0.83 |  | 0.34 | 0.01 | 0.39 |  |
| Holdemanella | 0.18 | 0.20 | 0.48 |  | 0.09 | 0.53 | 0.79 |  | 0.06 | 0.67 | 0.88 |  | 0.05 | 0.74 | 0.91 |  | 0.04 | 0.78 | 0.93 |  | 0.29 | 0.16 | 0.77 |  |
| Holdemania | -0.13 | 0.02 | 0.16 |  | -0.10 | 0.09 | 0.39 |  | -0.07 | 0.22 | 0.73 |  | -0.07 | 0.27 | 0.73 |  | -0.07 | 0.24 | 0.77 |  | -0.03 | 0.72 | 0.97 |  |
| Hungatella | 0.19 | 0.09 | 0.33 |  | 0.18 | 0.10 | 0.43 |  | 0.15 | 0.19 | 0.73 |  | 0.14 | 0.22 | 0.71 |  | 0.12 | 0.32 | 0.81 |  | 0.10 | 0.54 | 0.90 |  |
| Hydrogenoanaerobacterium | -0.22 | 0.03 | 0.18 |  | -0.20 | 0.05 | 0.33 |  | -0.13 | 0.20 | 0.73 |  | -0.12 | 0.25 | 0.71 |  | -0.12 | 0.27 | 0.79 |  | -0.02 | 0.90 | 0.97 |  |
| Incertae_Sedis | 0.04 | 0.55 | 0.78 |  | 0.05 | 0.43 | 0.72 |  | 0.06 | 0.36 | 0.80 |  | 0.05 | 0.47 | 0.85 |  | 0.04 | 0.54 | 0.87 |  | 0.09 | 0.37 | 0.85 |  |
| Intestinibacter | -0.27 | 0.02 | 0.14 |  | -0.19 | 0.10 | 0.42 |  | -0.26 | 0.03 | 0.34 |  | -0.26 | 0.04 | 0.37 |  | -0.25 | 0.04 | 0.40 |  | -0.21 | 0.22 | 0.77 |  |
| Intestinimonas | -0.03 | 0.78 | 0.91 |  | 0.0001 | 1.00 | 1.00 |  | 0.09 | 0.49 | 0.84 |  | 0.12 | 0.36 | 0.81 |  | 0.13 | 0.32 | 0.81 |  | 0.37 | 0.04 | 0.59 |  |
| Lachnoclostridium | 0.15 | 0.01 | 0.09 |  | 0.14 | 0.01 | 0.17 |  | 0.10 | 0.09 | 0.52 |  | 0.10 | 0.10 | 0.56 |  | 0.09 | 0.13 | 0.67 |  | -0.01 | 0.93 | 0.98 |  |
| Lachnospira | -0.41 | 0.001 | 0.02 |  | -0.35 | 0.003 | 0.08 |  | -0.35 | 0.004 | 0.13 |  | -0.37 | 0.003 | 0.13 |  | -0.33 | 0.01 | 0.20 |  | -0.14 | 0.42 | 0.89 |  |
| Lachnospiraceae_FCS020_group | -0.24 | 0.03 | 0.19 |  | -0.24 | 0.04 | 0.29 |  | -0.21 | 0.07 | 0.49 |  | -0.23 | 0.05 | 0.42 |  | -0.22 | 0.06 | 0.44 |  | -0.21 | 0.21 | 0.77 |  |
| Lachnospiraceae_ND3007_group | -0.51 | 0.003 | 0.05 |  | -0.51 | 0.003 | 0.08 |  | -0.55 | 0.002 | 0.09 |  | -0.53 | 0.003 | 0.13 |  | -0.51 | 0.005 | 0.20 |  | -0.35 | 0.16 | 0.75 |  |
| Lachnospiraceae_NK4A136_group | -0.43 | <.001 | 0.01 |  | -0.39 | 0.001 | 0.05 |  | -0.33 | 0.005 | 0.15 |  | -0.31 | 0.01 | 0.18 |  | -0.31 | 0.01 | 0.22 |  | -0.04 | 0.83 | 0.97 |  |
| Lachnospiraceae_UCG.001 | -0.32 | 0.04 | 0.23 |  | -0.31 | 0.05 | 0.33 |  | -0.24 | 0.13 | 0.65 |  | -0.27 | 0.10 | 0.56 |  | -0.24 | 0.15 | 0.69 |  | -0.08 | 0.72 | 0.97 |  |
| Lachnospiraceae_UCG.004 | -0.08 | 0.57 | 0.79 |  | -0.08 | 0.57 | 0.82 |  | -0.07 | 0.62 | 0.85 |  | -0.07 | 0.64 | 0.89 |  | -0.08 | 0.60 | 0.88 |  | -0.22 | 0.30 | 0.84 |  |
| Lachnospiraceae_UCG.010 | -0.09 | 0.34 | 0.62 |  | -0.13 | 0.21 | 0.56 |  | -0.08 | 0.41 | 0.83 |  | -0.08 | 0.47 | 0.85 |  | -0.08 | 0.46 | 0.84 |  | -0.09 | 0.56 | 0.91 |  |
| Lactobacillus | 0.10 | 0.43 | 0.69 |  | -0.01 | 0.93 | 0.96 |  | -0.11 | 0.38 | 0.82 |  | -0.18 | 0.15 | 0.65 |  | -0.19 | 0.12 | 0.64 |  | -0.02 | 0.89 | 0.97 |  |
| Lactococcus | -0.04 | 0.69 | 0.86 |  | -0.02 | 0.85 | 0.93 |  | -0.003 | 0.98 | 1.00 |  | -0.04 | 0.70 | 0.91 |  | -0.05 | 0.64 | 0.88 |  | -0.12 | 0.38 | 0.85 |  |
| Marvinbryantia | -0.40 | 0.01 | 0.08 |  | -0.41 | 0.01 | 0.11 |  | -0.40 | 0.01 | 0.19 |  | -0.40 | 0.01 | 0.20 |  | -0.42 | 0.01 | 0.20 |  | -0.33 | 0.13 | 0.75 |  |
| Megasphaera | 0.47 | 0.01 | 0.09 |  | 0.28 | 0.11 | 0.44 |  | 0.18 | 0.31 | 0.78 |  | 0.13 | 0.45 | 0.84 |  | 0.09 | 0.62 | 0.88 |  | -0.03 | 0.91 | 0.97 |  |
| Monoglobus | -0.24 | 0.04 | 0.21 |  | -0.13 | 0.26 | 0.60 |  | -0.08 | 0.52 | 0.85 |  | -0.07 | 0.57 | 0.87 |  | -0.06 | 0.64 | 0.88 |  | 0.14 | 0.42 | 0.89 |  |
| Moryella | -0.13 | 0.15 | 0.42 |  | -0.11 | 0.20 | 0.56 |  | -0.08 | 0.34 | 0.80 |  | -0.07 | 0.48 | 0.85 |  | -0.07 | 0.47 | 0.84 |  | 0.01 | 0.95 | 0.98 |  |
| Negativibacillus | 0.03 | 0.81 | 0.91 |  | 0.07 | 0.64 | 0.86 |  | 0.07 | 0.63 | 0.85 |  | -0.03 | 0.81 | 0.93 |  | -0.04 | 0.77 | 0.93 |  | -0.03 | 0.90 | 0.97 |  |
| NK4A214_group | -0.33 | 0.06 | 0.27 |  | -0.35 | 0.05 | 0.33 |  | -0.25 | 0.17 | 0.73 |  | -0.35 | 0.06 | 0.44 |  | -0.35 | 0.06 | 0.44 |  | -0.23 | 0.38 | 0.85 |  |
| Odoribacter | 0.09 | 0.62 | 0.81 |  | 0.11 | 0.57 | 0.82 |  | 0.20 | 0.29 | 0.77 |  | 0.22 | 0.25 | 0.71 |  | 0.20 | 0.32 | 0.81 |  | 0.46 | 0.10 | 0.71 |  |
| Oscillibacter | -0.20 | 0.06 | 0.27 |  | -0.16 | 0.13 | 0.49 |  | -0.10 | 0.36 | 0.80 |  | -0.12 | 0.29 | 0.75 |  | -0.13 | 0.25 | 0.77 |  | 0.12 | 0.44 | 0.89 |  |
| Oscillospira | -0.07 | 0.35 | 0.62 |  | 0.004 | 0.96 | 0.99 |  | 0.01 | 0.86 | 0.96 |  | 0.01 | 0.92 | 0.98 |  | 0.01 | 0.88 | 0.95 |  | 0.19 | 0.10 | 0.71 |  |
| Oxalobacter | 0.17 | 0.11 | 0.37 |  | 0.16 | 0.14 | 0.49 |  | 0.19 | 0.08 | 0.50 |  | 0.18 | 0.10 | 0.56 |  | 0.24 | 0.03 | 0.32 |  | 0.29 | 0.06 | 0.63 |  |
| Paludicola | -0.19 | 0.07 | 0.30 |  | -0.09 | 0.38 | 0.70 |  | 0.002 | 0.99 | 1.00 |  | -0.03 | 0.80 | 0.92 |  | -0.004 | 0.97 | 0.99 |  | 0.24 | 0.11 | 0.74 |  |
| Parabacteroides | -0.08 | 0.57 | 0.79 |  | -0.08 | 0.57 | 0.82 |  | -0.11 | 0.45 | 0.84 |  | -0.14 | 0.35 | 0.81 |  | -0.15 | 0.34 | 0.83 |  | -0.27 | 0.21 | 0.77 |  |
| Paraprevotella | 0.04 | 0.83 | 0.92 |  | -0.05 | 0.82 | 0.93 |  | 0.01 | 0.96 | 0.99 |  | -0.06 | 0.79 | 0.92 |  | -0.10 | 0.64 | 0.88 |  | -0.34 | 0.25 | 0.80 |  |
| Parasutterella | -0.14 | 0.45 | 0.71 |  | -0.11 | 0.56 | 0.82 |  | -0.11 | 0.57 | 0.85 |  | -0.06 | 0.79 | 0.92 |  | 0.03 | 0.89 | 0.96 |  | -0.30 | 0.29 | 0.84 |  |
| Peptococcus | -0.22 | 0.02 | 0.16 |  | -0.17 | 0.08 | 0.39 |  | -0.13 | 0.20 | 0.73 |  | -0.14 | 0.19 | 0.70 |  | -0.13 | 0.21 | 0.77 |  | -0.002 | 0.99 | 1.00 |  |
| Phascolarctobacterium | -0.11 | 0.58 | 0.79 |  | -0.13 | 0.54 | 0.80 |  | -0.04 | 0.85 | 0.96 |  | -0.03 | 0.90 | 0.96 |  | 0.02 | 0.93 | 0.97 |  | 0.06 | 0.85 | 0.97 |  |
| Phocea | -0.05 | 0.51 | 0.75 |  | -0.06 | 0.47 | 0.75 |  | -0.09 | 0.26 | 0.76 |  | -0.09 | 0.28 | 0.75 |  | -0.11 | 0.22 | 0.77 |  | -0.25 | 0.04 | 0.59 |  |
| Prevotella | 0.28 | 0.21 | 0.48 |  | 0.17 | 0.44 | 0.72 |  | 0.19 | 0.41 | 0.83 |  | 0.14 | 0.54 | 0.86 |  | 0.18 | 0.44 | 0.83 |  | 0.23 | 0.49 | 0.89 |  |
| Romboutsia | -0.74 | <.001 | <.001 |  | -0.65 | <.001 | <.001 |  | -0.68 | <.001 | <.001 |  | -0.67 | <.001 | <.001 |  | -0.66 | <.001 | <.001 |  | -0.69 | 0.00 | 0.03 |  |
| Roseburia | -0.31 | 0.004 | 0.07 |  | -0.23 | 0.03 | 0.29 |  | -0.25 | 0.03 | 0.34 |  | -0.26 | 0.03 | 0.32 |  | -0.27 | 0.02 | 0.29 |  | -0.09 | 0.59 | 0.93 |  |
| Ruminococcus | -0.60 | <.001 | 0.01 |  | -0.54 | 0.001 | 0.07 |  | -0.48 | 0.01 | 0.15 |  | -0.49 | 0.01 | 0.18 |  | -0.45 | 0.01 | 0.22 |  | -0.06 | 0.80 | 0.97 |  |
| Sellimonas | -0.14 | 0.21 | 0.48 |  | -0.17 | 0.16 | 0.51 |  | -0.10 | 0.40 | 0.83 |  | -0.12 | 0.33 | 0.78 |  | -0.12 | 0.33 | 0.82 |  | 0.04 | 0.83 | 0.97 |  |
| Senegalimassilia | -0.05 | 0.70 | 0.87 |  | -0.14 | 0.28 | 0.61 |  | -0.14 | 0.28 | 0.77 |  | -0.15 | 0.24 | 0.71 |  | -0.14 | 0.29 | 0.79 |  | 0.16 | 0.38 | 0.85 |  |
| Shuttleworthia | -0.20 | 0.09 | 0.33 |  | -0.10 | 0.39 | 0.70 |  | -0.03 | 0.81 | 0.93 |  | -0.07 | 0.57 | 0.86 |  | -0.09 | 0.44 | 0.83 |  | -0.16 | 0.34 | 0.84 |  |
| Slackia | 0.08 | 0.58 | 0.79 |  | -0.06 | 0.67 | 0.87 |  | -0.08 | 0.60 | 0.85 |  | -0.05 | 0.75 | 0.92 |  | -0.04 | 0.79 | 0.93 |  | -0.04 | 0.83 | 0.97 |  |
| Streptococcus | -0.003 | 0.98 | 0.98 |  | -0.11 | 0.27 | 0.60 |  | -0.11 | 0.29 | 0.77 |  | -0.22 | 0.03 | 0.34 |  | -0.22 | 0.02 | 0.30 |  | -0.31 | 0.02 | 0.51 |  |
| Subdoligranulum | -0.12 | 0.33 | 0.61 |  | -0.10 | 0.44 | 0.72 |  | -0.04 | 0.76 | 0.91 |  | -0.03 | 0.83 | 0.93 |  | -0.02 | 0.88 | 0.95 |  | 0.24 | 0.22 | 0.77 |  |
| Sutterella | -0.35 | 0.11 | 0.36 |  | -0.47 | 0.03 | 0.27 |  | -0.47 | 0.04 | 0.41 |  | -0.37 | 0.11 | 0.58 |  | -0.40 | 0.09 | 0.53 |  | -0.47 | 0.15 | 0.75 |  |
| TM7x | -0.10 | 0.26 | 0.54 |  | -0.11 | 0.23 | 0.56 |  | -0.12 | 0.23 | 0.73 |  | -0.13 | 0.20 | 0.70 |  | -0.11 | 0.28 | 0.79 |  | -0.14 | 0.31 | 0.84 |  |
| Turicibacter | -0.09 | 0.32 | 0.60 |  | -0.03 | 0.73 | 0.89 |  | -0.05 | 0.62 | 0.85 |  | -0.06 | 0.54 | 0.86 |  | -0.06 | 0.51 | 0.86 |  | -0.04 | 0.76 | 0.97 |  |
| Tuzzerella | 0.01 | 0.95 | 0.97 |  | 0.09 | 0.49 | 0.76 |  | 0.10 | 0.47 | 0.84 |  | 0.08 | 0.58 | 0.87 |  | 0.11 | 0.44 | 0.83 |  | 0.0002 | 1.00 | 1.00 |  |
| UBA1819 | 0.05 | 0.59 | 0.80 |  | 0.06 | 0.50 | 0.77 |  | 0.11 | 0.24 | 0.73 |  | 0.11 | 0.24 | 0.71 |  | 0.08 | 0.39 | 0.83 |  | 0.22 | 0.10 | 0.71 |  |
| UCG.002 | -0.49 | 0.01 | 0.08 |  | -0.53 | 0.004 | 0.09 |  | -0.40 | 0.03 | 0.38 |  | -0.46 | 0.02 | 0.25 |  | -0.44 | 0.02 | 0.29 |  | -0.22 | 0.41 | 0.88 |  |
| UCG.003 | -0.39 | 0.02 | 0.13 |  | -0.51 | 0.002 | 0.07 |  | -0.47 | 0.01 | 0.15 |  | -0.47 | 0.01 | 0.18 |  | -0.47 | 0.01 | 0.20 |  | -0.56 | 0.02 | 0.44 |  |
| UCG.005 | -0.37 | 0.01 | 0.10 |  | -0.32 | 0.03 | 0.26 |  | -0.21 | 0.16 | 0.72 |  | -0.24 | 0.12 | 0.58 |  | -0.21 | 0.18 | 0.74 |  | 0.06 | 0.78 | 0.97 |  |
| UCG.009 | -0.10 | 0.28 | 0.56 |  | -0.10 | 0.29 | 0.62 |  | -0.07 | 0.47 | 0.84 |  | -0.08 | 0.43 | 0.83 |  | -0.08 | 0.45 | 0.84 |  | 0.18 | 0.20 | 0.77 |  |
| Veillonella | -0.17 | 0.19 | 0.47 |  | -0.27 | 0.05 | 0.33 |  | -0.28 | 0.05 | 0.43 |  | -0.33 | 0.02 | 0.25 |  | -0.35 | 0.01 | 0.22 |  | -0.43 | 0.03 | 0.56 |  |
| Victivallis | -0.06 | 0.66 | 0.84 |  | -0.04 | 0.75 | 0.89 |  | 0.03 | 0.81 | 0.93 |  | 0.03 | 0.85 | 0.95 |  | 0.03 | 0.82 | 0.93 |  | 0.15 | 0.45 | 0.89 |  |
| Data source: The Coronary Artery Risk Development in Young Adults (CARDIA), 1985-2016  The false discovery rate (FDR) was adjusted using Benjamini-Hochberg method.  Model 1adjusted for sequencing run. Model 2 additionally adjusted for age, sex, race, field center, and education. Model 3 additionally adjusted for physical activity, smoking status, alcohol use, and diet quality score. In Model 4, medication use, such as proton pump inhibitor and lipid-lowering, was added. Model 5 included all covariates in Model 4 and added systolic blood pressure, diastolic blood pressure, and antihypertensive medication (Y/N). Model 6 included all covariates in Model 4 and further adjusted for diabetes duration. | | | | | | | | | | | | | | | | | | | | | | | | |

| Additional file 1: Table S3. Associations of gut microbial beta-diversity with insulin resistance, diabetes duration, and stages of diabetes^a,b^ | | | | | | | | | | | | | | | | | |
| --- | --- | --- | --- | --- | --- | --- | --- | --- | --- | --- | --- | --- | --- | --- | --- | --- | --- |
|  | **HOMA-IR** | | |  | **Diabetes duration** | | | | |  | **Stages of diabetes** | | | | | | |
|  | ≤ median |  | > median |  | Normal/ preDM |  | Newly diagnosed DM |  | Established DM |  | Normal |  | PreDM |  | Diabetes without treatment |  | Diabetes with treatment |
|  | (n=302) |  | (n=302) |  | (n=518) |  | (n=17) |  | (n=70) |  | (n=417) |  | (n=101) |  | (n=31) |  | (n=56) |
|  | p-value | | |  | p-value | | | | |  | p-value | | | | | | |
| **Overall** | 0.001 | | |  | 0.001 | | | | |  | 0.001 | | | | | | |
| **Pair comparison**^c^ |  |  |  |  |  |  |  |  |  |  |  |  |  |  |  |  |  |
| Diabetes duration |  |  |  |  |  |  |  |  |  |  |  |  |  |  |  |  |  |
| Normal/preDM | N/A | | |  | N/A |  | 0.08 |  | 0.001 |  | N/A | | | | | | |
| Newly diagnosed DM |  |  |  |  | 0.08 |  | N/A |  | 0.27 |  |  |  |  |  |  |  |  |
| Established DM |  |  |  |  | 0.001 |  | 0.27 |  | N/A |  |  |  |  |  |  |  |  |
| Stages of diabetes |  |  |  |  |  |  |  |  |  |  |  |  |  |  |  |  |  |
| Normal | N/A | | |  | N/A | | | | |  | N/A |  | 0.003 |  | 0.002 |  | 0.001 |
| Prediabetes |  |  |  |  |  |  |  |  |  |  | 0.003 |  | N/A |  | 0.145 |  | 0.002 |
| Diabetes without treatment |  |  |  |  |  |  |  |  |  |  | 0.002 |  | 0.145 |  | N/A |  | 0.004 |
| Diabetes with treatment |  |  |  |  |  |  |  |  |  |  | 0.001 |  | 0.002 |  | 0.004 |  | N/A |
| Data source: The Coronary Artery Risk Development in Young Adults (CARDIA), 1985-2016  HOMA-IR = Homeostatic Model Assessment for Insulin Resistance; DM = diabetes mellitus; N/A = not applicable.  a: Results were based on the fully adjusted model, including sequencing run, age, sex, race, field center, education, physical activity, smoking status, alcohol use, diet quality score, body mass index, and medication use, such as proton pump inhibitor and lipid-lowering.  b: Based on permutation multivariate analysis of variance (PERMANOVA) with 1,000 permutations. Only p-value was reported. HOMA-IR was dichotomized into two groups by the median (2.19). Diabetes duration was categorized to three groups: normal/prediabetes, newly diagnosed diabetes (duration < 5 years), and established diabetes (duration ≥ 5 years).  c: Based on PERMANOVA tests for each pair within diabetes duration and stages. | | | | | | | | | | | | | | | | | |

| Additional file 1: Table S4. Multivariable-adjusted associations of α diversity measures with insulin resistance, diabetes duration, and stages of diabetes | | | | | | | | | | | | | | |  |
| --- | --- | --- | --- | --- | --- | --- | --- | --- | --- | --- | --- | --- | --- | --- | --- |
|  | **HOMA-IR** | |  | **Diabetes duration** | |  | **Stages of diabetes**  (reference: normal, n=417) | | | | | | | | |
|  |  |  |  |  |  |  | **Prediabetes** | |  | **Diabetes without treatment** | |  | **Diabetes with treatment** | | |
|  | (n=604) | |  | (n=605) | |  | (n=101) | |  | (n=31) | |  | (n=56) | | |
|  | β | 95% CI |  | β | 95% CI |  | β | 95% CI |  | β | 95% CI |  | β | 95% CI | |
| **Shannon index** |  |  |  |  |  |  |  |  |  |  |  |  |  |  | |
| Model 5^a^ | -0.0002 | -0.04, 0.04 |  | -0.03* | -0.05, -0.004 |  | -0.13 | -0.35, 0.10 |  | -0.03 | -0.41, 0.34 |  | -0.29 | -0.59, 0.01 | |
| Model 6^b^ | 0.01 | -0.04, 0.05 |  | -0.02 | -0.05, 0.004 |  | -0.15 | -0.37, 0.08 |  | 0.20 | -0.26, 0.66 |  | -0.02 | -0.43, 0.40 | |
| **Richness** |  |  |  |  |  |  |  |  |  |  |  |  |  |  | |
| Model 5^a^ | -0.01 | -0.05, 0.04 |  | -0.04* | -0.06, -0.02 |  | -0.21 | -0.43, 0.02 |  | -0.25 | -0.62, 0.12 |  | -0.48* | -0.78, -0.19 | |
| Model 6^b^ | 0.01 | -0.03, 0.05 |  | -0.03* | -0.06, -0.01 |  | -0.21 | -0.43, 0.01 |  | 0.01 | -0.44, 0.45 |  | -0.20 | -0.61, 0.21 | |
| Data source: The Coronary Artery Risk Development in Young Adults (CARDIA), 1985-2016  HOMA-IR = Homeostatic Model Assessment for Insulin Resistance.  *: Significance based on p-value < .05.  a: Model 5 adjusted for sequencing run, sociodemographics (i.e., age, sex, race, field center, education), behavioral factors (physical activity, smoking status, alcohol use, and diet quality score), medication use (proton pump inhibitor and lipid-lowering), body mass index, systolic blood pressure, diastolic blood pressure and antihypertensive medication use.  b: For insulin resistance and diabetes duration, Model 6 adjusted for sociodemographics, behavioral factors, medication use (proton pump inhibitor and lipid-lowering), body mass index, and diabetes medication use; while for diabetes stages, Model 6 adjusted for sociodemographics, behavioral factors, medication use (proton pump inhibitor and lipid-lowering), body mass index, and diabetes duration. | | | | | | | | | | | | | | |  |
